# Multi-omics analysis of MRPL-13 as a tumor-promoting marker from pan-cancer to lung adenocarcinoma

**DOI:** 10.18632/aging.205104

**Published:** 2023-10-12

**Authors:** Xugang Zhong, Zeju He, Yong Fan, Li Yin, Zheping Hong, Yu Tong, Qing Bi, Senbo Zhu

**Affiliations:** 1 Center for Rehabilitation Medicine, Cancer Center, Department of Orthopedics, Zhejiang Provincial People’s Hospital affiliated to Qingdao University, Qingdao, Shandong, China; 2 Center for Rehabilitation Medicine, Department of Orthopedics, Zhejiang Provincial People’s Hospital (Affiliated People’s Hospital), Hangzhou Medical College, Hangzhou, Zhejiang, China

**Keywords:** MRPL13, LUAD, immune infiltrates, cuproptosis, M6A

## Abstract

Background: As a member of the mitochondrial ribosomal protein family, mitochondrial ribosomal protein L13 (MRPL13) is responsible for synthesizing mitochondrial proteins in cells. Several studies have indicated that MRPL13 is associated with the proliferation cycle, migration ability, apoptosis and autophagy of cancer cells. However, a thorough examination of MRPL13 across cancers remains uncertain. Therefore, we tried to clarify the relationship between MRPL13 and pan-cancer, and verified it in lung adenocarcinoma by various methods. Finally, our research is expected to reveal new targets for pan-cancer treatment and improve the prognosis of cancer patients.

Methods: Using bioinformatics tools, we quantified the differential expression of MRPL13 between cancer tissues and corresponding or noncorresponding normal tissues across cancers. We also analyzed the relationships between MRPL13 expression levels and several factors, including diagnosis, prognosis, mutation, functional signaling pathways, immune infiltration, RNA modification, and the relationship with cuproptosis-related genes. Furthermore, we studied the relationship between the expression level of MRPL13 across cancers and the change in cancer functional status through single-cell data. In addition, quantitative experiments (PCR and Western blot) proved that the expression of MRPL13 was significantly different between LUAD and control samples. Finally, the effect of knocking out MRPL13 on cancer cells was compared by gene silencing experiments. In summary, we used a combination of bioinformatics and experimental applications to study the potential roles of MRPL13 in cancer.

Results: After conducting a multidimensional analysis, we found that the application of MRPL13 multigroup analysis can effectively improve the diagnostic efficiency of various cancers and predict the prognosis of cancer. Moreover, MRPL13 in pan-cancer is related to the cancer immune infiltration pattern, methylation level and cuproptosis-related genes. Furthermore, single-cell data analysis showed that the modules of metastasis, EMT, cell cycle, DNA repair, invasion, DNA damage and proliferation were positively correlated with the expression of MRPL13 in LUAD (Lung adenocarcinoma), while the modules of hypoxia and inflammation were negatively correlated. Moreover, through quantitative experiments, we observed higher expression of MRPL13 in cancer tissues at the RNA or protein level. Knockdown of MRPL13 in LUAD led to decreased cancer cell survival, delayed tumor division and migration, reduced invasion, and increased cancer cell apoptosis.

Conclusions: Our study demonstrates the potential of using MRPL13 as a molecular biomarker for diagnosing and suggesting the prognosis of certain malignant tumors. Furthermore, our research shows that MRPL13 may be an effective therapeutic target for lung adenocarcinoma.

## INTRODUCTION

Cancer is widely recognized as the most lethal disease, causing immense physical and emotional distress to millions of patients, their families, and society. Lung cancer is known for its aggressive nature and is considered one of the deadliest forms of cancer worldwide. Non-small cell lung cancer (NSCLC) is the most common type of lung cancer, accounting for 80-85% of all cases, with the predominant histological subtype being lung adenocarcinoma. Thanks to the progress and popularization of imaging technology, testing methods and treatment methods, remarkable progress has been made in the screening, diagnosis and clinical treatment of lung adenocarcinoma. However, the 5-year survival rate remains low. Consequently, there is an urgent need to conduct in-depth research on the pathogenesis of lung adenocarcinoma, elucidate the relevant pathological principles and molecular markers, and establish a theoretical foundation to improve the screening rate, diagnostic accuracy and treatment level of the disease.

Mitochondrial ribosomal proteins (MRPs) are a group of proteins responsible for translating the mitochondrial code. They are located within the mitochondria of eukaryotic cells and have been associated with various tumors. MRPL13, a member of this family, is believed to drive tumor initiation and progression. On the theoretical basis of previous research, MRPL13 plays a part in polarization-related metabolic reprogramming of M2 macrophages, promoting the translation of PI3K and the STAT3 signal pathway activator Ric8b in a codon-dependent manner. This ultimately leads to the promotion of Wnt-driven intestinal tumor initiation [1]. Furthermore, MRPL13 is closely related to the cancer cell cycle, RNA processing (degradation/splicing) and the M-TORC1 pathway. *In vitro*, MRPL13 silencing significantly inhibits the proliferation of breast cancer cells and changes the expression pattern of EMT-related genes by eliminating the positive contribution of AKT and mTOR phosphorylation [2]. Furthermore, the dual fusion of MRPL13-ALK and PPP1-CB-ALK is related to the acquired drug resistance of high-grade first-line chemotherapy drugs for lung neuroendocrine tumors with EGFR mutation [3]. MRPL13 is involved in regulating cancer through potential pathways such as the MYC target, oxidative phosphorylation, PI3K/AKT/mTOR signal transduction, and the G2/M checkpoint. This promotes the transformation of macrophages to the M1 state and reduces the infiltration of antitumor T cells into the tumor microenvironment. MRPL13 knockdown inhibits the proliferation of lung adenocarcinoma cells [4]. In summary, MRPL13 plays a role as a tumor promoter in some cancers, promoting tumor growth, invasion, and drug resistance by affecting the conduction of carcinogenic signaling pathways, the change in the immune microenvironment, and the proliferation of the vascular matrix. However, there is still a lack of systematic research to explain the significance of MRPL13 in pan-cancer and lung adenocarcinoma. Our research aims to reveal the function of MRPL13 in multiple cancers in a groundbreaking way through biological information analysis and experimental verification, suggesting the possibility of MRPL13 as a new target to treat cancer.

After the rapid development of biosequencing tools, biosequencing data have been widely used to elucidate cancer mechanisms and screen molecular markers. In this study, we utilized TCGA, UCSC XENA, and GEO databases to extract multiple datasets, which were subsequently subjected to a variety of cutting-edge information analysis applications. For example, the UCSC dataset is used to detect the differential expression of MRPL13 between normal tissues and pan-cancerous tissues. The DESeq2 package was used to analyze the differentially expressed genes between patients with high and low expression of MRPL13, and based on this, the genetic differences between patients with high and low expression of MRPL13 were studied [5]. The interacting proteins of MRPL13 were screened in the IMEX database, and Cytoscape was used to visualize the protein-protein interaction (PPI) network [6, 7]. Based on our analysis, we identified MRPL13 as a potential regulator of tumor progression in various cancers. Further experimental validation showed that MRPL13 in lung adenocarcinoma tissue is higher than that in corresponding normal tissue in many aspects (RNA or protein). Knockdown of MRPL13 in lung adenocarcinoma inhibited cancer cell survival, delayed tumor division, reduced metastasis, and increased cancer cell apoptosis. Therefore, we believe that targeting MRPL13 is an important part of anti-lung adenocarcinoma pharmacological therapy.

## MATERIALS AND METHODS

### Comparative analysis of MRPL13 expression across cancers

We utilized the UCSC dataset (https://xenabrowser.net/datapages/) to examine the comparative expression of MRPL13 in 33 cancers and normal tissues. This dataset is primarily designed for the analysis of specific gene expression in tumors and normal tissues and provides visualization of contrast data as needed. Specifically, initially, the homogenized and standardized pan-cancer dataset was downloaded from TCGA. Subsequently, the expression data for the ENSG00000172172 (MRPL13) gene were extracted from each sample. Additionally, we screened the sample sources and transformation on each expression value. Finally, we eliminated any cancer type with fewer than 3 samples, resulting in a dataset of 33 cancer type expression data.

### Survival prognosis analysis

In addition to extracting the pan-cancer dataset and MRPL13 gene expression data from UCSC, we also extracted and transformed the data about the prognosis of pan-cancer from a prognosis study published in the journal Cell [8]. Subsequently, we obtained and transformed the data of TARGET’s follow-up time of more than 30 days from UCSC as a supplement. The exclusion criteria were cancers with fewer than 10 samples in total. Finally, according to the consensus, the relationship between gene expression and prognosis in each tumor was analyzed by constructing a Cox proportional hazard regression model, and the significance of prognosis was obtained by using the log-rank test. The overall survival and progression-free interval data of the top ten cancer types with differential MRPL13 expression were selected for visualization.

After dividing the patients equally according to the expression level, a Kaplan-Meier curve was used to draw the prognosis (OS, PFI) of cancer patients who expressed MRPL13 to different degrees. Finally, we utilized the GEO database (GSE81538/GSE68465/GSE65858/GSE66229) to verify the influence of MRPL13 expression level on prognosis in four types of cancer patients (BRCA/LUAD/HNSC/STAD).

### Diagnostic value of MRPL13 across cancers

We obtained the receiver operating characteristic (ROC) curve through the R package pROC to evaluate the diagnostic value of MRPL13 in 13 cancers with different expression levels [9]. The area under the ROC curve is directly proportional to the diagnostic accuracy. In detail, by setting multiple critical values with differences for genetic variables and then calculating the evaluation index corresponding to each critical value in real time, the most efficient variable is selected, and then a curve with sensitivity as the ordinate and 1-specificity as the abscissa is drawn.

### Protein level analysis

We obtained the protein expression data of MRPL13 in BRCA, LUAD, HNSC and their corresponding normal tissues from the CPTAC database [10]. Since the CPTAC database does not provide data on gastric adenocarcinoma and its corresponding normal tissue, we utilized samples with high and low microsatellite instability as substitutes. Additionally, we selected representative immunohistochemistry (IHC) images of BRCA, LUAD, HNSC, and STAD and representative immunohistochemical images of corresponding normal tissues in the HPA database. This database contains a vast number of IHC images of tumors and normal tissues.

### Coexpression gene network and enrichment analysis

The correlation between MRPL13 and other genes was analyzed through the “STAT” package [version 4.2.2] (basic package) in R language. The top 30 positively and negatively related genes were selected for thermal map display. Then, the genes coexpressed in two or more cancers were enriched and analyzed, mainly including KEGG (http://www.genome.jp/kegg/) and Gene Ontology (http://pantherdb.org/). The Cluster Profiler package in R language was used for enrichment analysis, and the visualization of the results was completed by the ggplot2 package.

### Genomic alterations of MRPL13 in pan-cancer

The protein structure of MRPL13 was visualized by PYMOL (https://pymol.org/2/). Genetic variation of MRPL13 in different tumor types was analyzed by cBioProtal (cBioPortal for Cancer Genomics). The mutation landscape of MRPL13 was shown by the processed pan-cancer SNV data. The MRPL13 protein structure diagram and the mutation’s precise location were analyzed by the “mutations” module. In addition, according to the mutation grouping, the prognosis of patients with four candidate cancers (BRCA, LUAD, HANC, and STAD) was analyzed. In addition, we also compared the somatic gene mutation rates of LUAD patients with high and low MRPL13 expression, and 426 (83.0%) of 513 mutation samples were used to draw graphs. Finally, the chi-square test was used to evaluate the difference in gene mutation frequencies in each group of samples. Furthermore, by downloading and transforming the tumor dryness score calculated by methylation characteristics of each tumor in previous studies and the expression of MRPL13 in pan-cancer, we drew a Lollipop Chart of their correlation [11]. Finally, we analyzed the pan-cancer correlation of MRPL13 in TBM, MSI, HRD, and PLO to find out which patients are the most sensitive and beneficial to immunotherapy.

### Differential gene screening and gene enrichment analysis

LUAD patients were divided into two groups according to the median overall expression as the critical point. (Supplementary Table 1). Then, the differentially expressed genes of the two groups of patients were extracted and visualized by using the R language DEseq2 and GGPLOT2 volcanic map. (Log FC > 1, P < 0.05) (Supplementary Material 1) [5]. Then, the differential genes were subjected to GSEA enrichment analysis (Supplementary Material 2).

### Immune infiltration analysis and immune checkpoints

In this study, we utilized three algorithms from various databases, TIMER, QUANTISEQ, and XCELL, to investigate the relationship between MRPL13 and immune cell infiltration patterns in different types of cancer. Moreover, we utilized the ssGSEA algorithm embedded in the GSVA package in R language to analyze the immune cell infiltration model based on the high and low expression of MRPL13 in BRCA, LUAD, HNSC, and STAD in TCGA. The data on immune cells were obtained from a published article [12]. Additionally, we used TISIDB analysis to identify five immune subtypes in BRCA, LUAD, STAD, and HNSC.

Chemokine genes, chemokine receptor genes, MHC-related immune pathway marker genes, immunosuppressant genes, and immunostimulant genes were collected from a standardized ubiquitin gene expression dataset. The coexpression relationship between MRPL13 and immune genes in pan-cancer was inferred using the R package “limma” and determined using the Pearson statistical method.

By downloading and transforming the uniform and standardized pan-cancer dataset and screening the sample sources, the expression profile of MRPL13 in each tumor was extracted, and then the expression profile was mapped to Gene-Symbol. Furthermore, the stromal, immune and ESTIMATE scores of each patient were counted, transformed and charted [13].

### Protein-protein interaction network and enrichment analysis

We screened for interacting proteins of MRPL13 in the IMEX database and visualized the protein-protein interaction (PPI) network using Cytoscape. Moreover, we performed KEGG/GO enrichment analysis on the binding proteins associated with MRPL13. We used the Cluster Profiler package (version 3.14.3) and the ggplot2 package (version 3.3.3) for enrichment analysis and visualization, respectively.

### Drug sensitivity analysis of MRPL13

We downloaded drug activity data and RNA-seq expression profiles from CellMiner™ to analyze the sensitivity of MRPL13 to drugs across cancers [14]. Moreover, to further investigate the possibility of MRPL13 as a new target for tumor treatment, we selected a group of genes related to MRPL13 and analyzed their relationship with drug sensitivity. For our analysis, we only considered FDA-approved drugs or drugs currently undergoing clinical trials.

### RNA modification analysis

We screened, extracted and homogenized the expression data of the ENSG00000172172 (MRPL13) gene and three RNA modification marker genes in each sample from the UCSC database. Then, we calculated the correlation between ENSG00000172172 (MRPL13) and five immune pathway marker genes by the Pearson correlation algorithm. The correlation heatmap is drawn. The three RNA methylation patterns analyzed in this work include m1A, m5C, and m6A. The three types of genes related to modification are writers, readers, and erasers.

### Correlation between MRPL13 and cuproptosis-related genes in LUAD

The standardized pan cancer dataset was downloaded from the TCGA database, and the expression data of MRPL13 and 13 cuproptosis-related genes expressed in BRCA, CHOL, ESCA, HNSC, LUAD, PPAD, and STAD were extracted. Moreover, the expression data of MRPL13-related genes and 13 cuproptosis-related genes expressed in LUAD were extracted. The Pearson correlation was calculated by log2(x+1) transformation of each expression value.

### Effects of MRPL13 on 14 functional states in cancer

The single-cell sequence data from the Cancer-SEA website were extracted [15], and the functional status changes in 14 cancers caused by MRPL13 were analyzed in pan-cancer by the Xiantao academic online tool. The effects of MRPL13 expression on tumor metastasis, EMT, the cell cycle, DNA repair, invasion, DNA damage, proliferation, hypoxia, and inflammation were extracted and shown.

### The result of querying candidate causal perturbations of MRPL13

A creative database of the human perturbation database (GPSAdb, https://www.gpsadb.com/) was used for genetic perturbation similarity analysis (GPSA) [16]. GPSA compared the DGE of 3048 disturbed datasets from GPSAdb to determine which genes lead to a similar trend of MRPL13 expression changes after knockout. To put it more succinctly, through the GPSAdb database, we found a gene that leads to changes in the expression of MRPL13 after knockout. To clarify the possible upstream regulatory factors of MRPL13.

### Specimen collection

We collected twenty fresh LUAD samples and twenty fresh normal lung tissues adjacent to the tumor from Zhejiang Provincial People’s Hospital (Hangzhou, China). The extracted sample was first placed at the liquid nitrogen lock temperature. This was followed by cell extraction, fragmentation, RNA and protein extraction, and other related steps. We employed qRT-PCR assays and Western blot analysis to analyze the samples. In addition, we obtained 50 IHC samples from the Pathology Department of Zhejiang Provincial People’s Hospital (Supplementary Table 2). All human samples were solely used for research purposes and were approved by the Ethics Committee of Zhejiang Provincial People’s Hospital (QT2023121).

### *In vitro* experiment

We used a qRT-PCR assay, immunohistochemistry (IHC) staining, and WB analysis to investigate the expression of MRPL13 in LUAD samples versus normal samples. Furthermore, we used CCK-8 reagent to evaluate the cell proliferation characteristics and cytotoxicity of each group and flow cytometry to evaluate apoptosis and cell cycle progression in the control and experimental groups. Finally, the distant metastasis ability of tumor cells was determined by the number difference of the same cells passing through the Transwell chamber at the same time. Please refer to Supplementary Material 3 for technical details.

### Statistical methods

Bioinformatics analysis was performed using R software and bioinformatics tools, including the Cancer-SEA database (CancerSEA - Database Commons (https://www.cncb.ac.cn/)) and GPSAdb database (https://www.gpsadb.com/). Statistical analysis was performed with SPSS software version 25.0 (IBM Corp., USA) and GraphPad Prism version 7.0 (GraphPad Software Inc., USA). The differential expression of MRPL13 among different samples and groups was determined by t tests. The “survival” and “forest-plot” packages were used to determine the HRs of MRPL13 across cancers. Kaplan-Meier curve reveals the relationship between MRPL13 expression and prognosis of patients with pan-cancer. The correlation coefficient is quantified by Pearson or Spearman. The Wilcox test was used to analyze immune infiltration genes. P-values less than 0.05 (*p < 0.05) were considered significant.

### Availability of data and material

The datasets generated during and/or analyzed during the current study are available from the corresponding author on reasonable request.

## RESULTS

### Differential expression of MRPL13 between tumor and normal tissue samples

**Figure 1:**
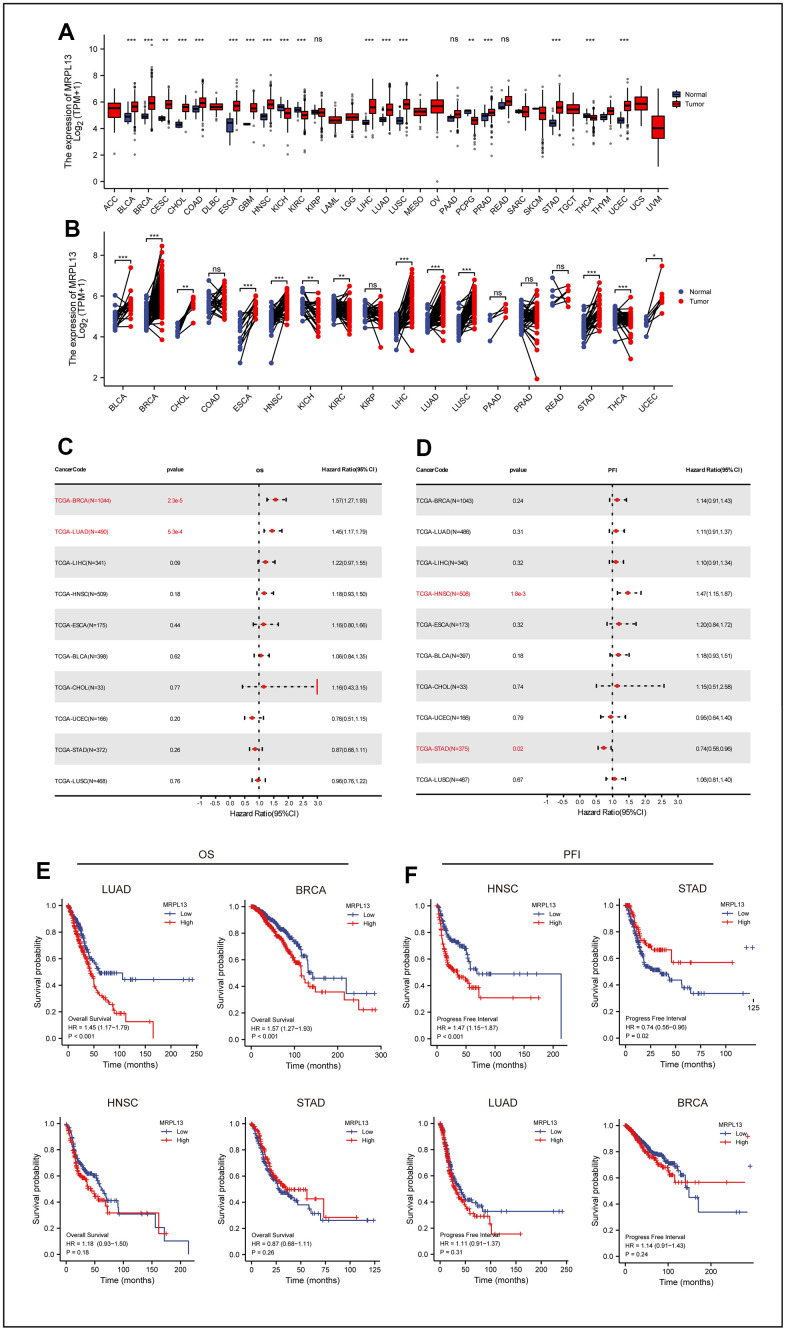
**Differential expression of MRPL13 in pan-cancer and the relationship between MRPL13 expression and prognosis of pan-cancer.** (**A**) Comparison of MRPL13 expression between tumor and normal samples. (**B**) Comparison of MRPL13 expression between tumor and paired normal samples. (**C**) Forest plot of OS associations in 33 types of tumors. (**D**) Forest plot of PFI associations in 33 types of tumors. (**E**, **F**) Kaplan–Meier analysis of the association between MRPL13 expression and OS/PFI. *p < 0.05, **p < 0.01, ***p < 0.001. ns, not statistically significant.

By comparing cancer and normal tissue data from TCGA, we identified differential expression of MRPL13 in 18 types of cancer, except for cancers lacking comparative data or differences. Compared with normal tissues, the expression of MRPL13 in BLCA, BRCA, CESC, CHOL, COAD, ESCA, GBM, HNSC, LIHC, LUAD, LUSC, PRAD, STAD, and UCEC is completely opposite to that in KICH, KIRC, PCPG, and THCA. The former was higher than that in normal tissue, while the latter was lower than that in normal tissue (Figure 1A). Paired samples showed similar results to unmatched samples in BLCA, BRCA, CHOL, ESCA, HNSC, KICH, KIRC, LIHC, LUAD, LUSC, STAD, UCEC, and THCA (Figure 1B). Additionally, to verify these findings at the protein expression level, we analyzed relevant immunohistochemical images from HPA data (Supplementary Figure 1A–1H).

### Prognostic significance of MRPL13 in cancer

To explore the influence of MRPL13 expression level on the prognosis of patients with pan-cancer from the macro level and to narrow the scope of our investigation, we analyzed the influence of MRPL13 as a single factor on the prognosis of each cancer by two indicators: overall survival (OS) and progression-free interval (PFI). The Cox proportional hazard model analysis revealed that the increased expression level of MRPL13 leads to poor prognosis in patients with BRCA and LUAD, and the result was statistically significant (P = 2.3e-5) (p=5.3e-4). MRPL13 was identified as a higher risk factor (Figure 1C). This result is consistent with the Kaplan-Meier survival analysis (Figure 1E).

The relationship between MRPL13 and PFI revealed that an increase in MRPL13 expression leads to an increase in adverse events in HNSC patients. (p = 1.8e-3), while it acted as a protective factor for STAD (p = 0.02) (Figure 1D). Kaplan-Meier survival analysis confirmed this trend (Figure 1F). Finally, using Kaplan-Meier survival analysis of the validated dataset in GEO, we confirmed that MRPL13 is a reliable factor for predicting the prognosis of cancer (Supplementary Figure 1I).

### Diagnostic value of MRPL13 across cancers

**Figure 2:**
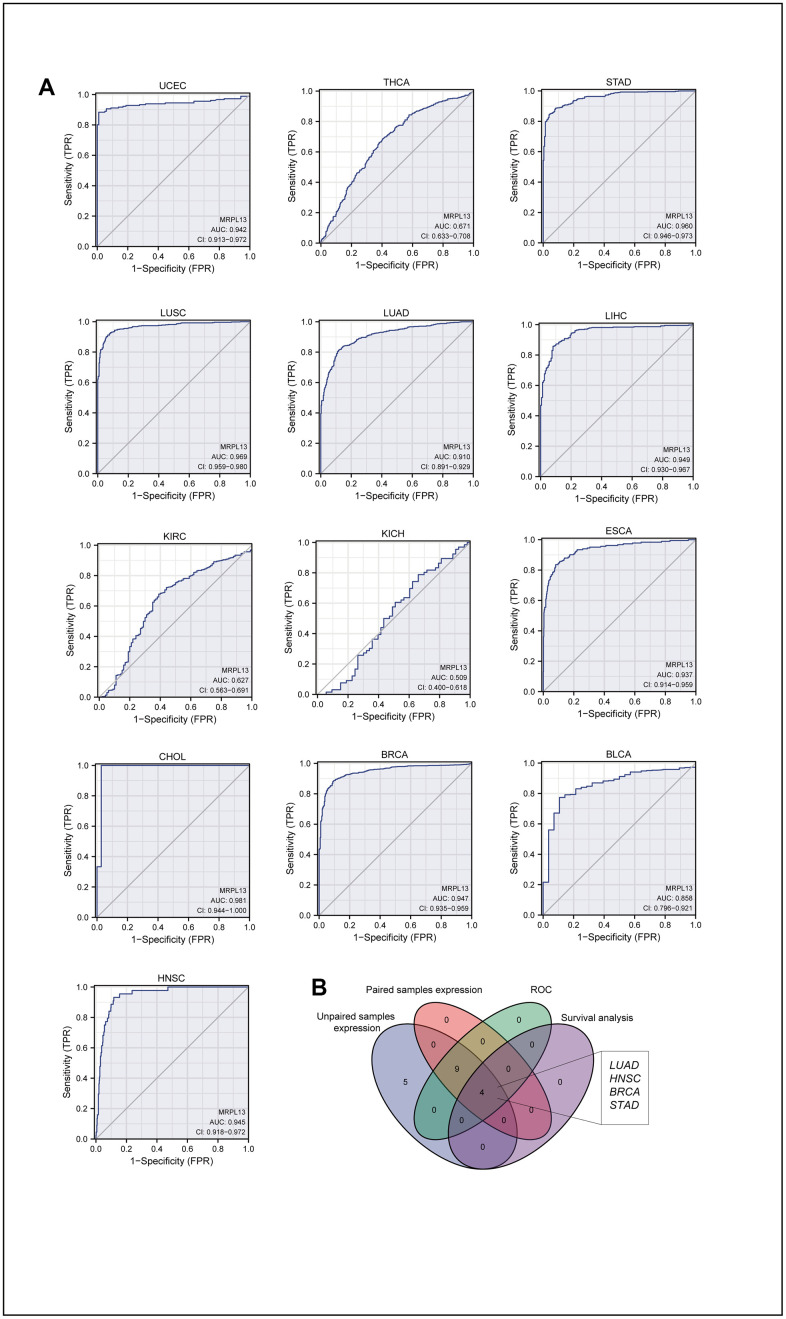
**AUC of ROC curves verified the diagnosis performance of MRPL13 in the TCGA cohort.** (**A**) ROC curve shows the value of MRPL13 in the diagnosis of patients with Pan-cancer, including UCEC, THCA, STAD, LUSC, LUAD, LIHC, KIRC, KICH, ESCA, CHOL, BRCA, BLCA, HNSC. (**B**) Venn diagram showed the intersection of cancer in pan-cancer, which was significant in expression, diagnostic performance and prognosis analysis.

Evaluation of the receiver operating characteristic (ROC) curve confirmed the diagnostic value of MRPL13 across cancers. The AUC value, ranging from 0.5 to 1, measures the accuracy of the diagnostic effect. An AUC of 1 indicates a better diagnostic effect. Analyzing the ROC curve of 13 cancers with differential MRPL13 expression obtained from the TCGA analysis of matched and unmatched samples, we found that the diagnostic accuracy of the AUC analyzed by this model was high in 9 cancers, relatively high in 1 cancer, and low in 2 cancers (Figure 2A). Finally, we constructed a Venn diagram to compare the cancer model with differences among unmatched sample comparison, matched sample comparison, ROC diagnosis model, and survival analysis, which identified BRCA, LUAD, STAD, and HNSC as significant cancers (Figure 2B).

### Coexpression network analysis of MRPL13 across cancers and coexpression network enrichment analysis

**Figure 3:**
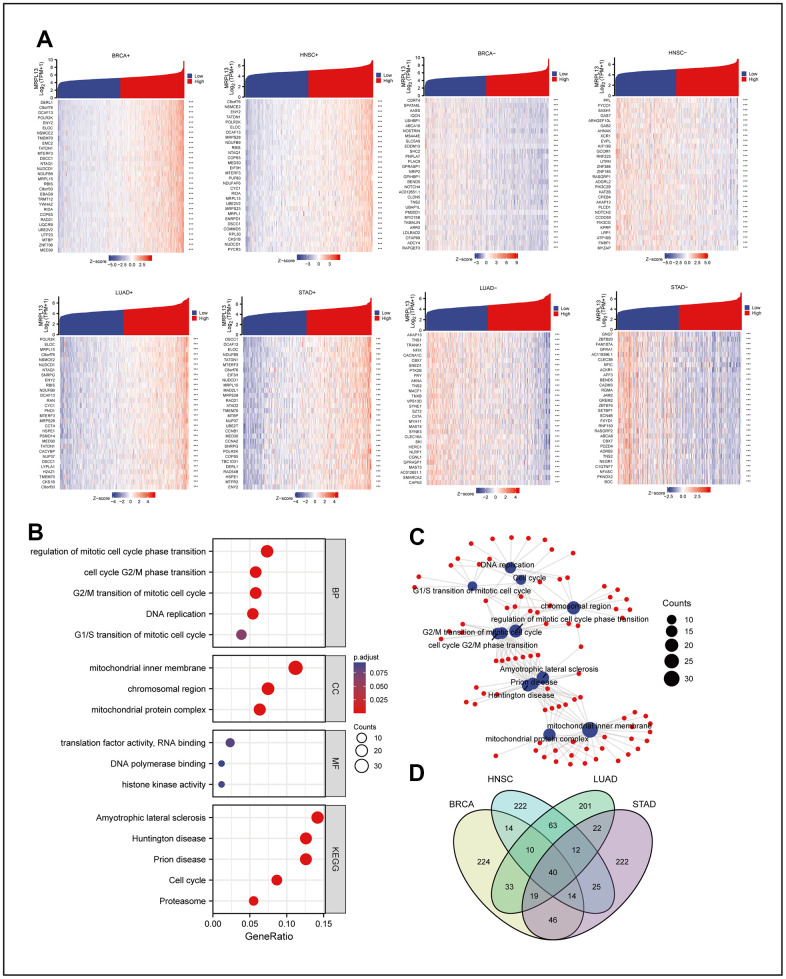
**Co-expression network analysis of MRPL13 in pan-cancer and co-expression network enrichment analysis.** (**A**) The top 30 positive and negative genes related to the expression of MRPL13 in BRCA, LUAD, HNSC and STAD. (**B**) GO and KEGG analyses for genes related to the expression of MRPL13 in at least two cancers (**C**) the result of GO and KEGG analyses visualize with R language pack ggplot2 (red: molecular: blue: enrichment results). (**D**) Veen map shows the intersection of genes related to the expression of MRPL13 in BRCA, LUAD, HNSC and STAD.

We analyzed the sequencing data of BRCA, LUAD, HNSC, and STAD in TCGA using R language, and the genes related to the expression of MRPL13 in each cancer were identified. We then generated a heatmap based on the log value of the top 30 genes (Figure 3A). Further analysis of the intersection of the genes that co-occur in at least two cancers was performed using the Cluster Profiler package in R, with GO terminology and KEGG pathway analysis. The results showed that genes were mainly related to the regulation of mitotic cell cycle phase transition, mitochondrial inner membrane, translation factor activity, and RNA binding in GO enrichment analysis. In KEGG enrichment analysis, the genes were mainly enriched in amyotrophic lateral sclerosis, Huntington disease, and prion disease (Figure 3B), which was visualized with the ggplot2 R language pack (Figure 3C). Crossing the genes related to MRPL13 in each cancer resulted in a gene set of 40 genes (Figure 3D) and (Supplementary Material 4). Finally, we performed a variety of enrichment analyses of these 40 genes in Metascape, and the results showed that in GO analysis, the top three were neddylation, ribonucleoprotein complex biogenesis and gastric cancer network 2. The cell cycle ranked fifth, which is consistent with the fact that MRPL13 may promote the growth of cancer cells by affecting the cell cycle. In addition, in the summary of enrichment analysis in cell type signatures, TRAVAGLINI LUNG PROLIFERATING NK T CELL ranks second. In the summary of enrichment analysis in DisGeNET, Monosomy is the closest. In the final summary of enrichment analysis in Transcription Factor Targets, the top three are PPARGC1A TARGET GENES, ZNF581 TARGET GENES and HSF2 TARGET GENES. Enrichment analysis revealed the common biological function of these 40 genes, which provides useful value for expounding the role of MRPL13 (Supplementary Figure 1J–1M).

### The genetic alterations of MRPL13

**Figure 4:**
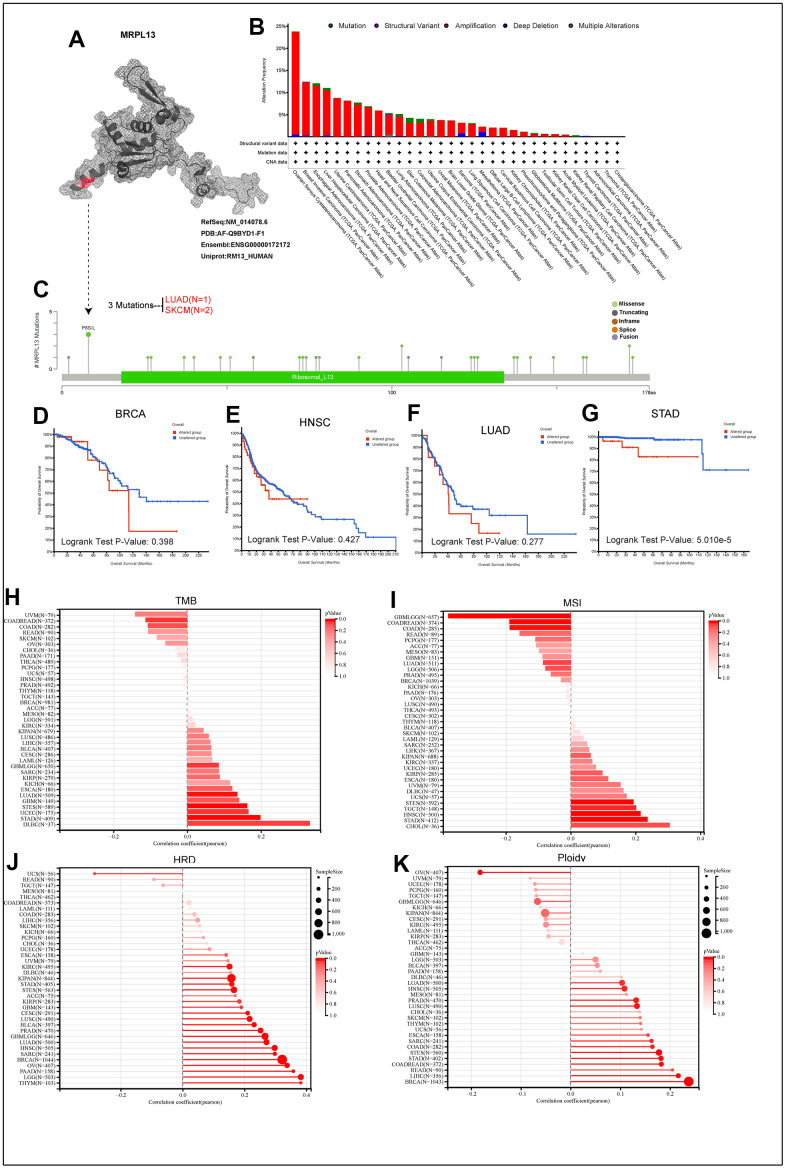
**The genetic alterations of MRPL13.** (**A**) The protein structure of MRPL13. (**B**) Analysis of MRPL13 alteration frequency in different tumor types according to cBioPortal dataset. (**C**) The mutation types, number, and sites of MRPL13 in pan-cancer analysis according to cBioPortal dataset. (**D**–**G**) Relationship between MRPL13 mutation status and overall survival in BRCA, LUAD, HNSC and STAD. (**H**–**K**) The association of TMB, MSI, HRD, PLOIDY with MRPL13 in pan-cancer.

Initially, the protein structure of MRPL13 was visualized using the PYMOL website (Figure 4A). The cBioPortal database revealed that the highest incidence of gene alterations in MRPL13 is in ovarian cancer, while it is also amplified in the majority of pan-cancer patients, including those with breast cancer, esophageal cancer, liver cancer, uterine cancer, pancreatic cancer, gastric adenocarcinoma, prostate cancer, head and neck squamous cell carcinoma, bladder cancer, and lung adenocarcinoma. Amplification, deletion mutation, and deep deletion are the most common types of genetic variation observed in MRPL13 (Figure 4B). The types, sites, and cases of MRPL13 gene modification are presented, with MRPL13 missense mutation being the most common mutation type and P8 mutation detected in 2 cases of SKCM and 1 case of LUAD (Figure 4C). Subsequently, we conducted an analysis of the patient prognosis across four types of cancer based on mutation groups, revealing a poor prognosis for patients in the MRPL13 mutation group in STAD (Figure 4D–4G). Moreover, to further explore the gene mutation caused by MRPL13 in patients with LUAD, we divided the patients into two groups and compared the mutation frequency differences between groups. The results showed that the top three gene mutation rates of cells with high and low expression of MRPL13 were TP53 (54.0%), TTN (51.3%) and CSMD3 (41.7%) (Supplementary Figure 2A) and (Supplementary Material 5).

We compared the correlation of TMB, MSI, HRD, and PLOIDY with MRPL13 across various cancer types. TMB was positively correlated with MRPL13 in six cancers, GBM, LUAD, STES, UCEC, STAD, and DLBC, but negatively correlated with MRPL13 in UVM, COADREAD, COAD, and READ (Figure 4H). MSI analysis showed that MRPL13 had a significant positive correlation with eight types of cancers. UVM, DLBCM UCS, STES, TGCT, HNSC, STAD, and CHOL were negatively correlated in six cancers, namely, GBMLGG, COADREAD, COAD, READ, PCPG, and ACC (Figure 4I). In addition, we observed that MRPL13 was significantly associated with HRD in 23 cancers, with a positive correlation in ESCA, UVM, KIRC, DLBC, KIPAN, STAD, STES, ACC, KIRP, GBM, CESC, LUSC, BLCA, PRAD, GBMLGG, LUAD, HNSC, SARC, BRCA, OV, PAAD, LGG, and THYM and a negative correlation with UCS (Figure 4J). Moreover, polyploidy, an indicator of cancer, was positively correlated with 19 cancers, including DLBC, LUAD, HNSC, MESO, PRAD, LUSC, CHOL, SKCM, THYM, UCS, ESCA, SARC, COAD, STES, STAD, COADREAD, READ, LIHC, and BRCA, but negatively correlated with seven cancers, including OV, UVM, UCEC, PCPG, TGCT, GBMLGG, and KICH (Figure 4K). Furthermore, the correlation between MRPL13 and the tumor dryness score in pan-cancer showed that MRPL13 was positively correlated with the tumor dryness score in most cancers (Supplementary Figure 2B–2G).

Finally, to obtain the alterations of MRPL13 and its expression data in pan-cancer, the expression difference of MRPL13 in different samples in each tumor was calculated by R software (version 4.1.3). The copy number data and gene expression data of each tumor sample were integrated, and the difference between two samples was analyzed by using unpaired Wilcoxon rank sum and signed rank tests. The difference between multiple groups of samples was tested by the Kruskal-Wallis test. Significant differences were observed in 23 tumors, including BRCA, LUAD, STAD and HNSC. MRPL13 mutation and gene expression significantly affect many cancer types (Supplementary Figure 3A). The mutation of MRPL13 in individual tumors analyzed by Sanger-Box is shown in Supplementary Figure 3B.

In general, MRPL13 acts as a potential marker of genomic stability in a variety of cancer species, including LUAD, and affects patient prognosis and therapeutic responses.

### GSEA enrichment analysis of differentially expressed genes in patients with different degrees of MRPL13 expression

**Figure 5:**
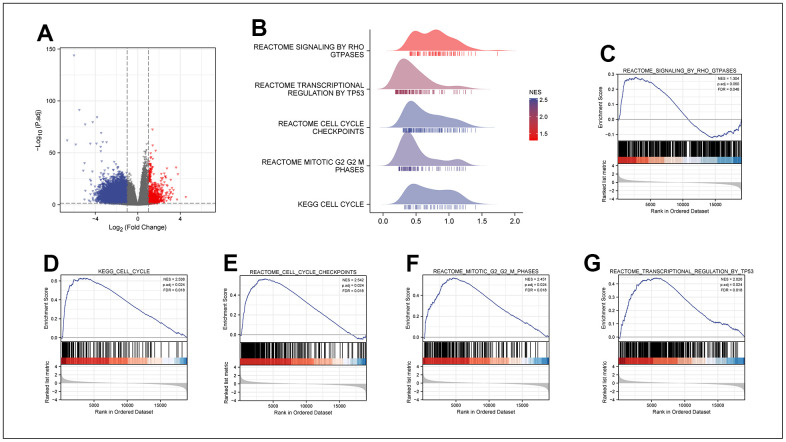
**DEGs between MRPL13 high and low expression groups in LUAD.** (**A**) The volcano map of DEGs (red: upregulation; blue: downregulation). (**B**) GSEA enrichment analysis results of high and low MRPL13 differential genes visualized in Ridgeline plot. (**C**–**G**) GO and KEGG analyses for samples with high and low MAZ expression in LUAD.

After analyzing the MPRL13 expression count of LUAD patients in the TCGA database. All LUAD patients were divided into high and low expression groups with the median MRPL13 expression as the critical point. By using R language DEseq2, the differentially expressed genes of the two groups were extracted (Log FC > 1, P < 0.05) and visualized by using a GGPLOT2 volcanic map (Figure 5A). Second, the differentially expressed genes were subjected to GSEA. The differentially expressed genes between the two groups were significantly enriched in several biological pathways, including REACTOME SIGNALING by RHO GTPASES, REACTOME TRANSCRIPTIONAL REGULATION by TP53, REACTOME CELL CYCLE CHECKPOINTS, REACTOME MITOTIC G2/G2 M PHASES, and KEGG CELL CYCLE (Figure 5B–5G). Based on the above results, we speculate that MRPL13 may participate in the regulation of tumor occurrence and development by affecting the cell cycle.

### Relationship of MRPL13 expression with immune infiltration analysis

**Figure 6:**
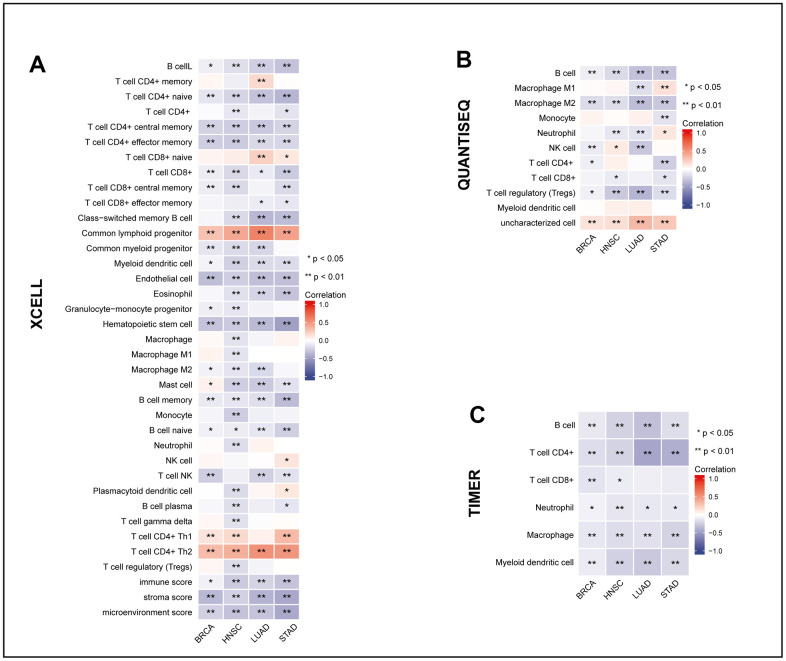
**The relationship between MRPL13 and tumor immune infiltrating cells in BRCA, LUAD, HNSC and STAD.** (**A**–**C**) The correlation between MRPL13 expression and immune cell infiltration was analyzed by XCELL, QUANTISEQ and TIMER algorithm. *p < 0.05, **p < 0.01, ns, not statistically significant.

To reveal the change in the immune cell infiltration pattern caused by MRPL13 in patients, we analyzed immune cell infiltration data extracted from various databases and algorithms. Using TIMER, QUANTISEQ, and XCELL, we mapped the MRPL13 gene expression of pan-cancer patients in the TCGA dataset to the corresponding immune cell infiltration and constructed a correlation heatmap. Infiltration of immune cells, including B cells and T cells from lymphocytes, monocytes, macrophages and dendritic cells from the myeloid system, appeared in four cancers (BRCA, LUAD, HNSC, and STAD). The results indicate a negative correlation between MRPL13 expression and the infiltration of various immune cells, such as CD8^+^ T cells, macrophages, and B cells (Figure 6). In addition, in a more detailed comparison, there were significant differences in the composition of immune cell infiltration, including CD8 T cells and NK cells, and the group with low expression of MRPL13 had a better antitumor immune cell infiltration microenvironment. (Supplementary Figure 4A–4D). These findings suggest that MRPL13 may affect tumor prognosis by modulating the host immune response.

**Figure 7:**
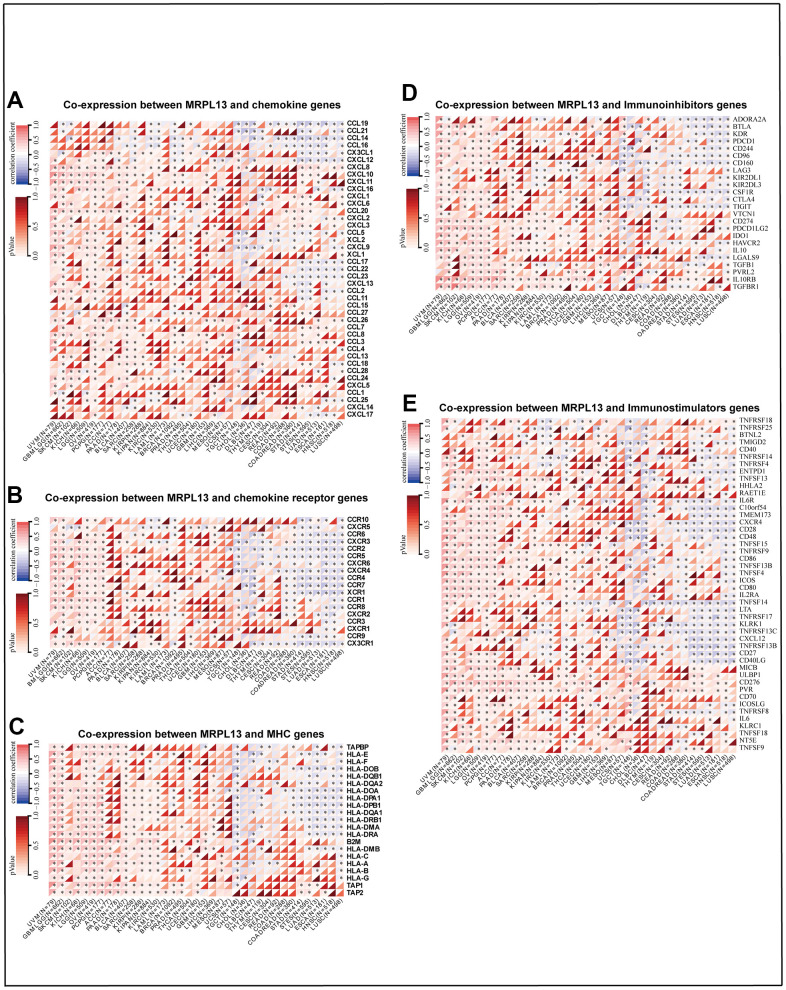
**Heatmaps indicating the co-expression of MRPL13 with immune-relevant genes in pan-cancer.** (**A**) Co-expression between MRPL13 and chemokine genes. (**B**) Co-expression between MRPL13 and chemokine receptor genes, (**C**) Co-expression between MRPL13 and MHC genes. (**D**) Co-expression between MRPL13 and Immunoinhibitors genes. (**E**) Co-expression between MRPL13 and Immunostimulators gene. *p-value < 0.05, **p-value < 0.01, ***p-value < 0.001, and ****p-value < 0.0001.

We also analyzed the relationship between MRPL13 and immune infiltration-related genes by a coexpression algorithm. The results indicated that MRPL13 and most immune-related genes had the same change trend in specific cancer types. such as UVM, GBML GG, SKCM, KIHC, LGG, OV, ACC, PAAD, and BLCA. However, in BRCA, STAD, STES, LUAD, ESCA, HNSC, and LUSC, it was negatively correlated. In particular, chemokines, including CXCL8, CXCL10, CXCL11, CXCL16, CCL26, and CCL7, as well as chemokine receptors, including CXCR3, CCR2, CCR5, and CCR1, were positively correlated with MRPL13 expression in various cancer types. (Figure 7A, 7B). MHC genes and MRPL13 were coexpressed in almost all tumor types, except TGCT, STAD, STES, LUAD, ESCA, HNSC, and LUSC (Figure 7C). Furthermore, immune-stimulating factors and immunosuppressive factors were closely associated with the expression of MRPL13 across cancers. Almost all cancers showed a significant relationship between MRPL13 and immune checkpoint suppressor genes, such as VEGFA, CD274 (PD-L1), and CTLA4 (Figure 7D, 7E). Finally, TISIDB analysis demonstrated that MRPL13 expression was considerably enriched in six immune subtypes, including wound healing, IFN dominance, inflammation, lymphocyte depletion, immune calm, and TGFβ dominance, in BRCA, LUAD, HNSC, and STAD (Supplementary Figure 4E–4H).

Furthermore, MRPL13 was negatively correlated with the infiltration of various stromal immune cells in the immune microenvironment. Specific B cells, CD4+ T cells, macrophages, and neutrophils are shown in Supplementary Figure 5A.

Finally, we extracted and mapped the expression profile of MRPL13 in patients with LUAD according to the coordination relationship between MRPL13 and immune gene expression. The results were counted and converted into related scores by using the R language package. It was concluded that the expression of MRPL13 in patients with LUAD was inversely proportional to these three scores (Supplementary Figure 5B–5D). Overall, the above results show that MRPL13 is closely associated with the biological functions of several immune-related cells and genes.

### PPI network and enrichment analysis of MRPL13-binding proteins

**Figure 8:**
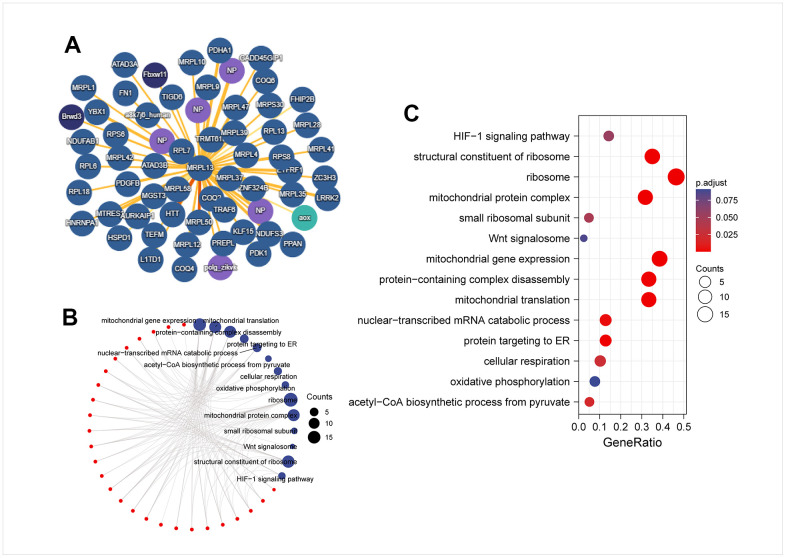
**PPI network and enrichment analysis of MRPL13-binding proteins.** (**A**) PPI network; (**B**) visual network of GO analysis (red: molecular; blue: enrichment results). (**C**) KEGG/GO analysis.

We used the IMEX database to identify the targeted binding protein of MRPL13 (Figure 8A). Subsequently, we analyzed the KEGG/GO enrichment of these targeted binding proteins and found that they mainly participate in several biological processes for instance mitochondrial gene expression, protein complex disassembly, and mitochondrial translation. The molecular function analysis revealed involvement in nuclear-transcribed mRNA catabolic process and protein targeting to the endoplasmic reticulum. The cellular component analysis indicated an association with ribosomes and mitochondrial protein complexes. Moreover, KEGG analysis showed that MRPL13 binding proteins were mainly involved in cellular respiration and the acetyl-CoA biosynthetic process from pyruvate (Figure 8B, 8C).

### Drug sensitivity analysis of MRPL13

**Figure 9:**
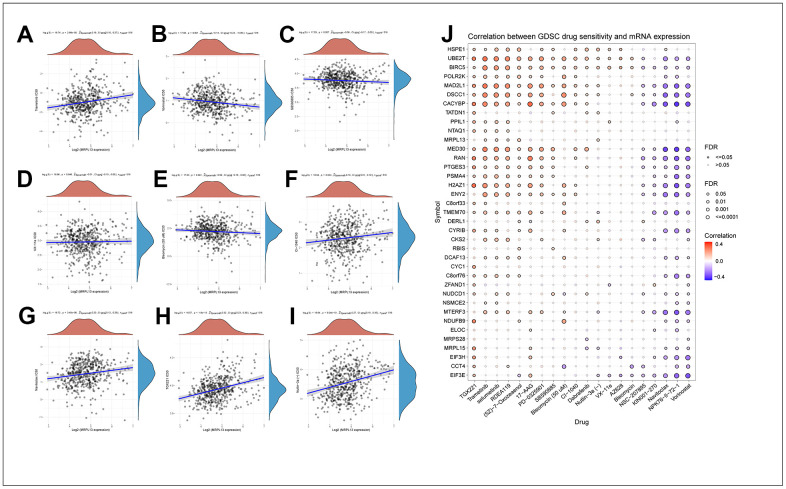
**Drug sensitivity analysis of PNPO and the genes that MRPL13-related.** (**A**) Trametinib. (**B**) Vorinostat. (**C**) SB590885. (**D**) VX−11e. (**E**) Bleomycin. (**F**) CI-1040. (**G**) Navitoclax. (**H**) TGX221. (**I**) Nutlin-3A. (**J**) Correlation between GDSC drug sensitivity and the genes that MRPL13-related.

Using the Cell Miner database, we conducted further screening for therapeutic drugs related to MRPL13 and analyzed the effect of MRPL13 expression on the sensitivity of patients to molecular drugs. Our results showed that the expression of the MRPL13 gene in patients with LUAD was positively correlated with patients’ sensitivity to tramadol, CI-1040, navitoclax, TGX221 and nutlin-3A. Conversely, the expression of MRPL13 was negatively correlated with sensitivity to vorinostat, SB590885, VX-11e and bleomycin. (Figure 9A–9I). These findings suggest that high MRPL13 expression may significantly impact the effectiveness of drug treatment for cancer patients, including immunotherapy.

Furthermore, by drawing a correlation heatmap between MRPL13-related genes and the sensitivity to 20 common drugs, we found a direct proportion between MRPL13 and most related gene expression levels and the sensitivity to TGX221, trametinib, and selumetinib and an inverse proportion to navitoclax, NPK76-II-72-1, vorinostat, and other drugs (Figure 9J). These findings demonstrate the crucial role that MRPL13 and its related genes play in influencing drug treatment sensitivity, which suggests potential targeted therapies for cancer through MRPL13 and related genes.

### MRPL13 and RNA methylation modification markers

The expression data (UCSC database) of MRPL13 and 44 marker genes of three RNA modification genes in each sample of the pan cancer dataset were extracted and converted by formula. Then, we drew a heatmap between MRPL13 and the marker genes of five immune pathways through the results of the Pearson correlation algorithm. The results demonstrate a strong positive correlation between MRPL13 and most genes involved in modifying RNA methylation in some types of cancer, including ACC, KICH, BRCA, LUAD, and STAD. However, there is no obvious correlation between MRPL13 and methylation genes in other types of cancer, for instance UCS, DLBC, LIHC, GBM, and CHOL. These results provide direct evidence that MRPL13 is involved in the regulatory network and complexity of multiple cancers (Supplementary Figure 6). The alteration of RNA methylation is highly significant in cancer development and treatment response.

### Correlation between MRPL13 and cuproptosis-related genes in LUAD

**Figure 10:**
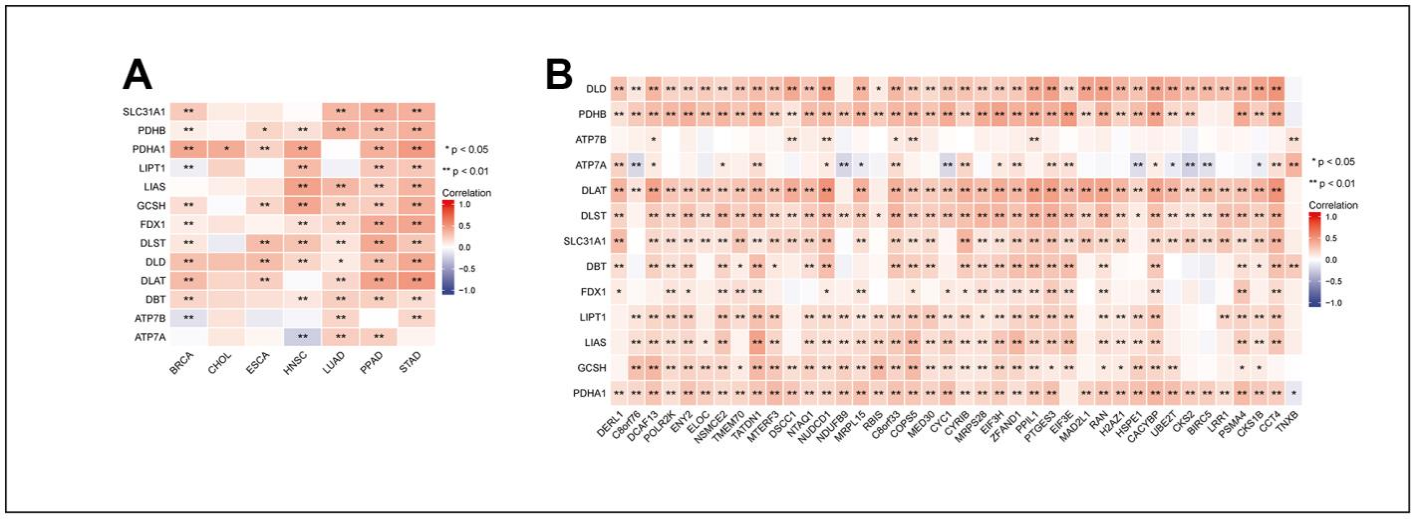
**Correlation between MRPL13 and the genes that MRPL13-related with cuproptosis related genes in pan-cancer.** (**A**) Correlation between the expression levels of MRPL13 and cuproptosis related genes in pan-cancer. (**B**) Correlation between the expression levels of the genes that MRPL13-related and cuproptosis related genes. * p < 0.05, ** p < 0.01.

We used the expression data of MRPL13 and cuproptosis-related genes in the TCGA database to study the relationship between them through a correlation algorithm. Our analysis revealed that in CHOL, there was no correlation between MRPL13 and most cuproptosis-related genes. However, in BRCA, HNSC, LUAD, PPAD, and STAD, MRPL13 showed a significant correlation with GCSH, FDX1, DLST, and DLD (Figure 10A). To further discuss the relationship between MRPL13 and cuproptosis in detail, we also drew a correlation heatmap between MRPL13-related genes and cuproptosis-related genes. Our findings indicate that except for ATP7A and ATP7B, most cuproptosis-related genes are directly proportional to MRPL13-related genes (Figure 10B). These results indicate that MRPL13 regulates cancer cell status in some types of cancer through cuproptosis-related mechanisms.

### Effects of MRPL13 on 14 functional states in cancer

**Figure 11:**
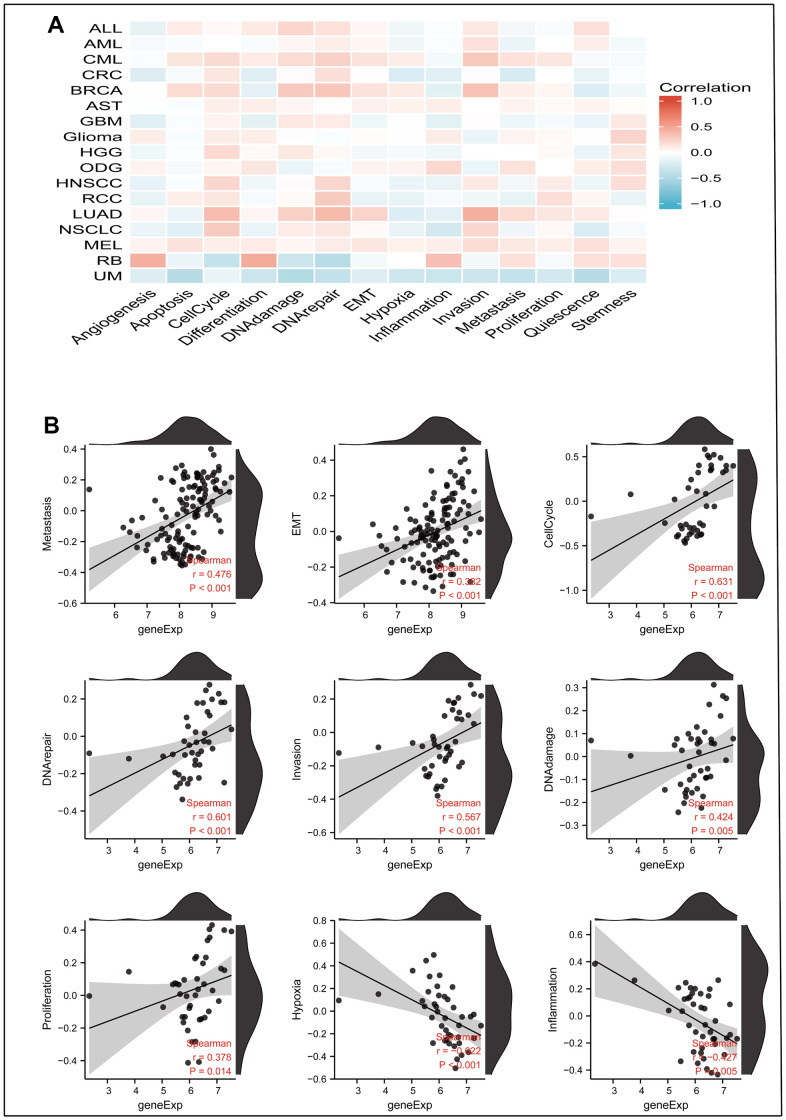
**The correlation between MRPL13 expression and 14 cancer functional states using single-cell sequence data from the Cancer-SEA database.** (**A**) The correlation between MRPL13 expression and 14 cancer functional states in pan-cancer. (**B**) The expression of MRPL13 is positively correlated to Metastasis, Cell-Cycle, DNA-repair, Invasion, DNA-damage, Proliferation of LUAD. The expression of CENPL is negatively correlated to the Hypoxia, Inflammation of LUAD.

By downloading the single-cell sequencing data from the CancerSEA database and using Xiantao Academic’s online mapping service, we explained the influence of MRPL13 on the functional status of 14 kinds of cancers. In most cancers, MRPL13 is directly proportional to most cell cycle-related marker genes. Among CML and LUAD, MRPL13 is most closely related to all fourteen cancer functional states. Interestingly, MRPL13 was negatively associated with hypoxia in most tumors (Figure 11A). In LUAD, the expression of MRPL13 was directly proportional to metastasis (r=0.476), EMT (r=0.382), the cell cycle (r=0.631), DNA repair (r=0.601), invasion (r=0.576), and DNA damage (r=0.424). However, it was inversely proportional to hypoxia (r=-0.622) and inflammation (r=-0.427) (Figure 11B).

### The result of querying candidate causal perturbations of MRPL13

In GPSAdb, we collected 3048 RNA-seq datasets of gene knockdown/knockout and analyzed them for differences. Using the query tool in GPSAdb, we identified the genes regulating MRPL13 from the 3048 RNAseq datasets with genetic perturbations. We found that FOXM1 knockout caused changes in MRPL13 expression in LUAD. Subsequently, we analyzed the correlation between FOXM1 and MRPL13 across various cancers and created a correlation map. The results showed that MRPL13 was directly proportional to FOXM1 in BLCA (r=0.468), BRCA (r=0.406), LGG (r=0.318), LUAD (r=0.349), OV (r=0.487), PAAD (r=0.567), PRAD (r=0.28), STAD (r=0.5), and TGCT (r=0.694) (Supplementary Figure 7).

### MRPL13 expression and functional analysis in LUAD

The above analysis results show that the expression level of MRPL13 is not only different between various tumor samples and normal tissues but also leads to the occurrence and development of many cancers. Given the comprehensive nature of these findings and the replication of previous studies, we deemed it necessary to conduct additional experiments to explore its differential expression in lung adenocarcinoma and normal tissues, as well as its general function in lung adenocarcinoma.

**Figure 12:**
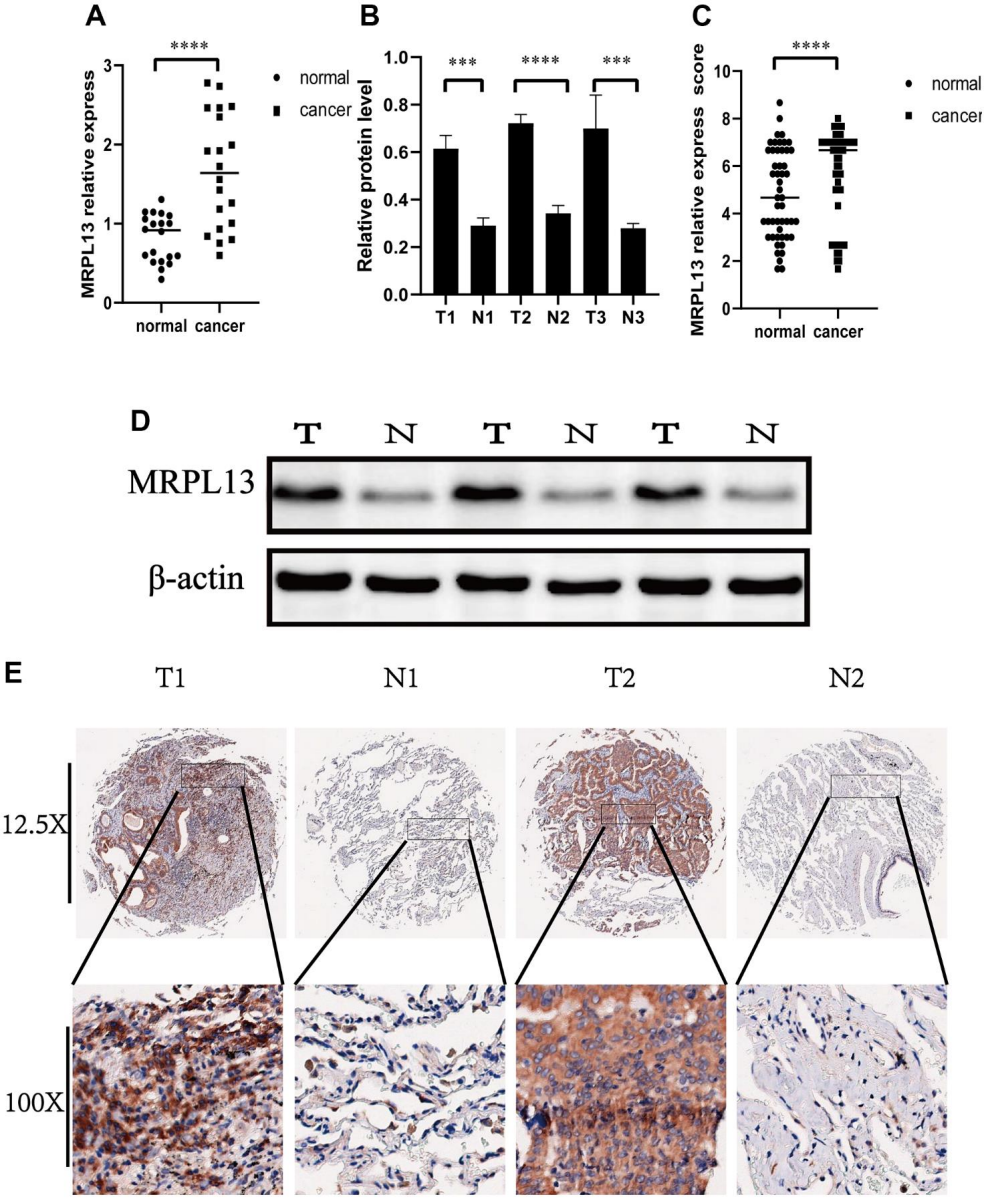
**Expression of MRPL13 in cancer and normal tissues.** (**A**) Fluorescence real-time quantitative PCR was used to detect the expression of MRPL13 in tumor tissues and normal tissues adjacent to cancer. (**B**) Western blot to verify the expression of MRPL13 in cancer and normal tissues adjacent to cancer (the experiment was repeated thrice). (**C**) Immunohistochemistry score to verify the expression of MRPL13 in cancer and normal tissues adjacent to cancer. (**D**) Western blot representative picture. (**E**) Immunohistochemical representative pictures of cancer patients and normal patients (panorama above, detail below). *p-value < 0.05, **p-value < 0.01, ***p-value < 0.001, and ****p-value < 0.0001.

We began by investigating whether MRPL13 functions as a tumor-promoting factor in lung adenocarcinoma. Real-time fluorescence quantitative PCR was employed to examine the expression of 20 clinical samples of lung adenocarcinoma, confirming once more that compared with the normal tissue group, the expression of MRPL13 in the LUAD group was higher (Figure 12A). To confirm these results at the protein level, we employed western blotting to assess three representative specimens and immunohistochemistry to assess 50 pathological sections. The comprehensive evaluation score of MRPL13 protein was significantly different between the cancer tissue group and adjacent normal tissue group (Supplementary Material 6). The former is obviously higher than the latter (Figure 12B–12E). The expression score was independently assessed by three experienced pathologists and confirmed through independent inspection and analysis.

**Figure 13:**
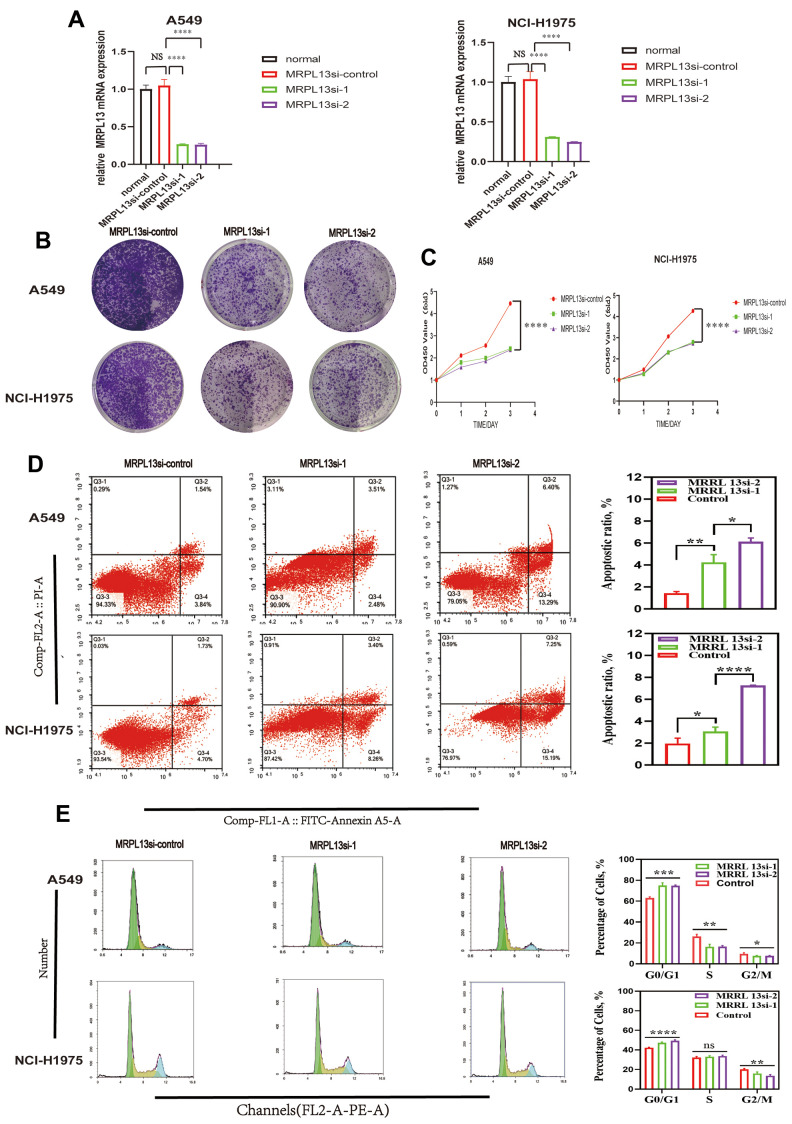
**Effect of MRPL13 down-regulation on the proliferation, cell cycle, apoptosis of LUAD.** (**A**) MRPL13 knockdown validation in A549 and NCI-H1975. (**B**, **C**) CCK8 and Colony formation detection of the effect of down-regulation of MRPL13 expression on the proliferation of LUAD cells. (**D**) The percentage of apoptosis in control group and MRPL13 down-regulated group was analyzed by flow cytometry. (**E**) The effect of low expression of MRPL13 on cell cycle distribution was detected by flow cytometry. *p-value < 0.05, **p-value < 0.01, ***p-value < 0.001, and ****p-value < 0.0001.

We chose A549 and NCI-H1975 lung cancer cell lines for further experiments based on their expression of MRPL13 and experimental feasibility. We successfully knocked down MRPL13 expression in these cell lines using siRNA, as confirmed by RT-PCR analysis (Figure 13A).

CCK8 assays and colony-forming tests showed that MRPL13 knockdown significantly reduced the proliferation of both A549 and NCI-H1975 cells compared to the control groups (Figure 13B, 13C).

Additionally, MRPL13 knockdown led to a significantly higher percentage of apoptotic cells in both cell lines (Figure 13D). Flow cytometry analysis revealed that MRPL13 knockdown altered the cell cycle distribution in both A549 and NCI-H1975 cells. The percentage of cells in G1 phase in the MRPL13 knockout group increased, while the percentage of cells in S and G2 phases decreased. (Figure 13E). These results revealed that MRPL13 can promote the growth of tumor tissue by regulating the proliferation and apoptosis of lung adenocarcinoma cells.

**Figure 14:**
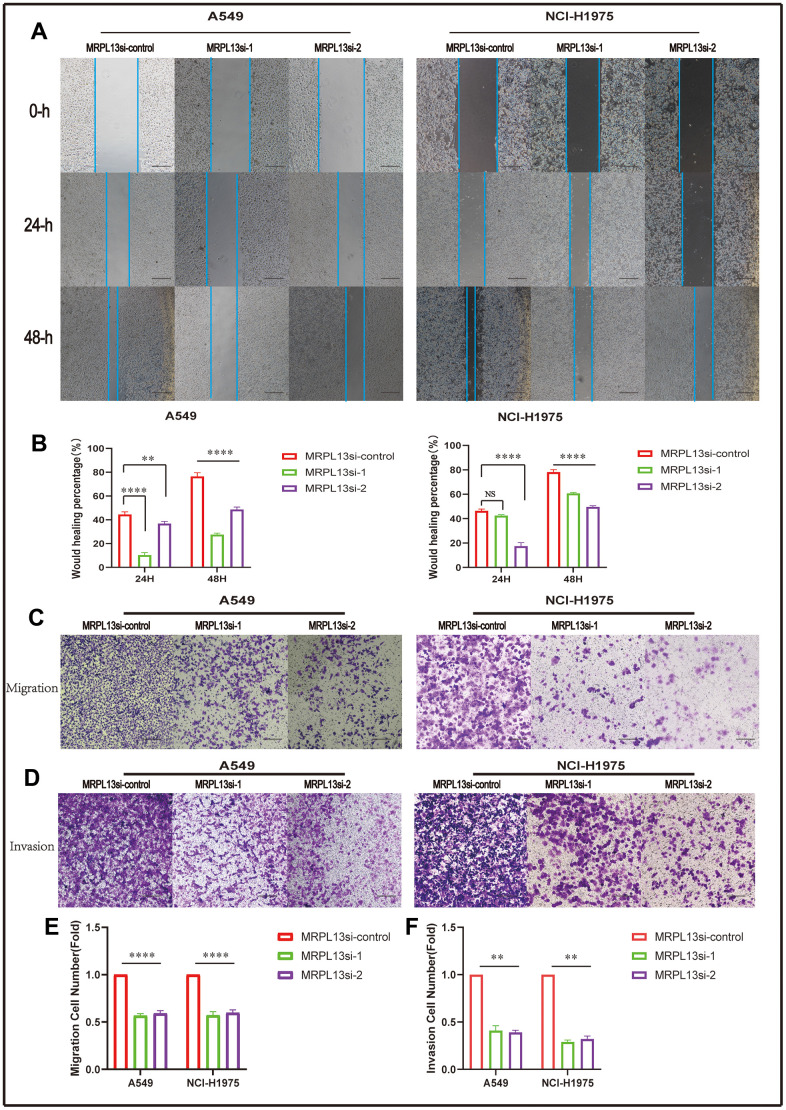
**Effect of MRPL 13 down-regulation on the migration of LUAD.** (**A**, **B**) The cell migration ability of MRPL13 knockout group and control group was compared by wound healing test (scale bar = 200 μm). (**C**–**E**) The cell migration ability of MRPL13 knockout group and control group was compared by Transwell test (scale bar = 200 μm). (**D**–**F**) The cell invasion ability of MRPL 13 knockout group and control group was compared by Transwell test (scale bar = 200 μm). **p-value < 0.01 and ****p-value < 0.0001.

The migration and invasion abilities of lung adenocarcinoma cells were evaluated by comparing the number of cells passing through the Transwell chamber, which demonstrated that MRPL13 knockout significantly reduced the ability of A549 and NCI-H1975 cells to migrate and invade. This finding was further confirmed by the scratch wound healing assay (Figure 14 and Supplementary Materials 7–10).

Our findings demonstrate that MRPL13 knockout negatively impacted the cell activity, proliferation, periodic differentiation, apoptosis, migration, and invasion of lung adenocarcinoma cells. Thus, MRPL13 represents a potential therapeutic target for lung adenocarcinoma and other cancers.

## DISCUSSION

As one of the most detrimental diseases in modern society, cancer imposes an extremely high burden on patients, families, and society. Despite the improvement of the tertiary prevention system, there is still a lack of diversified and individualized plans for tumor treatment. Pan-cancer analysis can aid in identifying similarities and differences in the pathogenesis of cancers and provide a theoretical basis for personalized cancer treatment. Additionally, it facilitates the efficient screening of molecular markers and identification of gene targets for cancer prevention and treatment.

Lung adenocarcinoma is one of the most common tumors, and research on individualized diagnosis and treatment is increasingly abundant. MRPL13, a member of the MRPL family, has been mentioned in several studies. For instance, Liu Y et al. demonstrated that MRPL13 can be treated as a prognostic molecular marker that can promote the growth, division, metastasis and invasion of breast cancer cells as a catalyst and ultimately lead to poor prognosis of patients. Min S et al. revealed that low expression of MRPL13 can lead to ribosome defects and weaken the oxidative phosphorylation ability of mitochondria [17], which play a crucial role in tumor progression. Therefore, targeting MRPL13 may have unexpected effects on cancer treatment. Moreover, a few studies have suggested that MRPL13 promotes tumor cell activity. However, the systematic study of the role of MRPL13 in the promotion of cancer occurrence, progression, metastasis, and prognosis, from pan-cancer to lung adenocarcinoma, is still unclear. Thus, our research is the first to comprehensively and systematically investigate this relationship using various bioinformatics analysis tools and *in vitro* experiments to verify our conclusions.

Our findings revealed that the expression level of MRPL13 between tumor and normal tissue is significantly different in many cancers. Specifically, we observed upregulation of MRPL13 in BLCA, BRCA, CESC, CHOL, COAD, ESCA, GBM, HNSC, LIHC, LUAD, LUSC, PRAD, STAD, and UCEC, while KICH, KIRC, PCPG, and HCA exhibited downregulation. To confirm these results at the RNA and protein levels, we utilized PCR, WB, and IHC analysis on our samples and data from public databases for lung adenocarcinoma. Additionally, our analysis of TCGA data indicated that MRPL13 expression is a prognostic indicator for several cancer types, including LUAD, BRCA, HNSC, and STAD. This study is the first to report a correlation between MRPL13 and cancer prognosis across multiple cancer types.

As a well-recognized diagnostic evaluation tool in the current era [18], the area under the receiver operating characteristic (ROC) curve can accurately evaluate the diagnostic efficiency of the gene model, with a value closer to 1 indicating a better diagnostic effect. The evaluation of the ROC curve demonstrated that MRPL13 is highly valuable in diagnosing pan-cancer, including LUAD, and underscores its significance in aiding the diagnosis and treatment of clinical patients.

The top 30 positively and negatively correlated genes with MRPL13 were identified in BRCA, LUAD, HANS, and STAD. The intersection of genes coexpressed in two or more cancers was analyzed and extracted, and then the differentially expressed genes were analyzed by gene ontology and KEGG pathway enrichment through algorithms. For example, ELOC, located in the 8q area, can increase the growth rate and invasiveness of prostate cancer cells and is related to the differentiation of hormone resistance in prostate cancer [19], bringing great challenges to the prevention and treatment of prostate cancer. ELOC-mutant RCC can show variable behavior ranging from very indolent to aggressive [20]. Additionally, MRPL15, which belongs to the same family as MRPL13, promotes the malignant progression of non-small cell lung cancer and affects the inhibitory effect of the immune system on tumor cells [21]. C8orf76 directly binds to carcinogenic lncRNA, induces its expression, and activates the MAPK signal. C8orf76 plays a crucial role in the occurrence of gastric cancer and can serve as an independent prediction model to evaluate the clinical outcome of patients with gastric cancer [22]. NUDCD1, ENY2, PNO1, CCT4 and PSMD14 are considered to promote the proliferation, invasion and treatment resistance of tumor cells in a variety of cancers, including pancreatic cancer, rectal cancer, liver cancer, breast cancer, glial cancer and head and neck squamous cell carcinoma [23–26]. Furthermore, nivolumab (NIVO) can also protect tumor cells from treatment by upregulating several genes, including DCAF13 [27]. Moreover, MRPL13 can also promote the malignant progression of tumors by inhibiting the expression of protective genes, such as NIFX and CBX7 [28, 29]. All of these results indicate that the MRPL13 gene may interact with many genes and play a role in multiple cancers through various pathways, which is an interesting phenomenon that warrants further investigation. The enrichment analysis of biological processes indicates that MRPL13 may regulate mitotic cell cycle phase transition, cell cycle regulation, and DNA replication across cancers. These pathways have been mentioned in previous studies [30]. The enrichment analysis of cellular components suggests that MRPL13 is related to mitochondrial intima and mitochondrial protein complexes, as well as translation factor activity and RNA binding. Other enriched pathways included amyotrophic lateral sclerosis and Huntington disease. These findings indicate that MRPL13 can not only affect the tumor process itself but also form a complex regulatory network by regulating coexpressed genes and ultimately promote the formation and development of tumors.

Somatic mutations are critical in determining tumor heterogeneity. By comparing the mutations of patients with high and low MRPL13 expression, it is concluded that MRPL13 may make tumor cells escape the cell cycle checkpoint, avoid apoptosis and aging, and gain abnormal proliferation ability by affecting the classical TP53 mutation. Tumor mutation burden (TMB) is an immunotherapy biomarker that reflects the genetic potential of the immune system in resisting tumor rejection. Patients with TMB mutations are more likely to benefit from the efficacy of PD-1/PD-L1 inhibitors [31]. Cancer with a positive correlation between TMB and MRPL13 may benefit more from treatment with immune checkpoint inhibitors. Moreover, microsatellite instability (MSI) refers to a special mutation of new microsatellite alleles due to the insertion or deletion of repetitive units compared with normal tissues. Cancer cells in MSI patients may have greater variation and are more easily recognized by the immune system. Patients with high MSI typically exhibit immunogenicity and extensive T-cell infiltration, which leads to a good clinical response to immune checkpoint inhibitors [32]. Furthermore, homologous recombination defects (HRDs) result in defects in the repair pathway of DNA double-strand breaks. HRD is highly sensitive to platinum drugs and PARP inhibitors that cause DNA breaks, making HRD an essential biomarker in ovarian cancer treatment [33]. Furthermore, chromosome instability involved in cancer development is the principle of polyploid mutation. Polyploidy is a common indicator of tumors and is closely related to tumor prognosis. Estimating the tumor’s purity and ploidy is beneficial to understanding cancer genome evolution and heterogeneity within the tumor [34]. Finally, in the analysis of gene expression and dryness score, we found that the expression of MRPL13 was directly proportional to the dryness score of cancer cells in most pan-cancers. It has been proven that MRPL13 can prevent treatment by promoting cell self-renewal and strengthening drug resistance. The malignant growth of the tumor is realized. In conclusion, MRPL13 could be a potential predictive biomarker for immunotherapy of malignant tumors, including lung adenocarcinoma (LUAD).

The tumor microenvironment is a complex ecosystem that includes tumor cells, tumor-related cells, immune and inflammatory cells, interstitial tissues, microvessels and various cytokines and chemokines [35, 36]. The type and quantity of immune cell infiltration largely determine the effect of immunotherapy, the possibility of immune escape, and the drug resistance of immunotherapy [37]. Our study showed that high expression of MRPL13 is negatively correlated with immune cell infiltration in BRCA, LUAD, HNSC, and STAD. The low expression group of MRPL13 in lung adenocarcinoma showed higher levels of CD8^+^ T cells, B cells, NK cells, and macrophages. These immune cells have positive effects on antitumor immunity. Macrophages may directly kill tumor cells in the early stage, while NK cells, T cells, and B cells may play an antitumor role in subsequent specific immunity [38, 39]. Another main finding is that in most pan-cancers, including UVM, GBMLGG, SKCM, KICH, and LGG, MRPL13 is positively correlated with most chemokines, receptors, and immune-stimulation and immunosuppression marker genes and negatively correlated with a few pan-cancers, such as ESCA, HNSC, and LUSC. Additionally, MRPL13 showed a strong relationship with immune checkpoint suppressor genes such as VEGFA, CD274 (PD-L1), and CTLA4 across various tumors. Finally, we obtained inverse relationships between MRP13 expression and stromal, immune, and ESTIMATE scores in patients with LUAD. MRPL13 can weaken the antitumor immune effect by affecting the tumor immune microenvironment. Therefore, MRPL13 may exert a negative influence in regulating the immune response of cancer and act as a potential molecular marker for the benefits of immunotherapy, especially in LUAD. Patients with low expression of MRPL13 may benefit more from immunotherapy.

Cuproptosis, a new type of cell death mentioned recently, is induced by copper ion carriers and differs significantly from traditional types of cell death, such as apoptosis, ferroptosis, and necrosis. The discovery of cuproptosis provides a new approach to exploring the treatment mechanism of tumors. Therefore, it is essential to examine the relationship between molecular markers and cuproptosis-related genes. Our study showed that MRPL13 and its associated genes were significantly correlated with cuproptosis-related genes across cancers, with the most significant ones being FDX1, GCSH, DLST, and DLD. FDX1, which plays a crucial role in oxidative stress, trace copper transport, and mitochondrial metabolism, and has been shown in previous studies to be closely related to cuproptosis [40]. GCSH regulates extracellular acidification by controlling the release of lactate dehydrogenase, and low expression leads to damage to cell metabolic activity [41]. DLST, an important gene related to cuproptosis, controls glutamine from entering the tricarboxylic acid cycle (TCA) for oxidative decarboxylation, and its deletion inhibits the production of NADH and damages OXPHOS, leading to cell growth stagnation and apoptosis [42]. In head and neck squamous cell carcinoma, dihydrolipoamide dehydrogenase regulates cell death through cystine deprivation [43]. Our results suggest that MRPL13 may influence genes that regulate tumor progression, which provides insight into the potential role of MRPL13 in cuproptosis and tumor treatment.

We investigated the changes in the functional status of 14 cancers caused by MRPL13. By downloading the single-cell data in the CancerSEA database and uploading the data to Xiantao Academic to draw a correlation map, it was found that the expression of MRPL13 is significantly related to the cell cycle in most cancers. The cell cycle is a crucial biological process in cancer cell differentiation and has been a major area of focus in cancer research. Early studies identified inhibitors of cyclin and the cyclin-dependent kinase complex as part of the cell cycle checkpoint and proposed molecular approaches to prevent DNA-damaged cells from replicating their DNA. Recent studies have shown that the cell cycle mechanism regulates cell adhesion specifically, primarily through cyclin-dependent kinase 1 (CDK1), and have elucidated the molecules that mediate crosstalk between cell adhesion and the cell cycle [44]. In lung adenocarcinoma (LUAD), MRPL13 expression was found to be directly proportional to metastasis, epithelial-mesenchymal transition (EMT), the cell cycle, DNA repair, invasion, DNA damage, and proliferation but inversely proportional to hypoxia and inflammation. These findings suggest that MRPL13 affects tumor prognosis through multiple pathways.

Moreover, downstream targets of a gene can be easily identified through the PPI network and related analysis methods, providing a strong foundation for subsequent experimental analyses. However, discovering the upstream molecules of a target gene has always been a challenge in bioinformatics analysis. In this study, the GPSAdb database was employed to identify the upstream gene FOXM1, which may induce changes in MRPL13 expression after knockout in lung adenocarcinoma cell lines. Furthermore, the correlation between MRPL13 expression and FOXM1 expression was confirmed in various pan-cancers, which enriches the theoretical framework of MRPL13’s role in promoting the occurrence and progression of pan-cancer. Nevertheless, the limitation of this analysis is that the obtained relationship between MRPL13 and FOXM1 is not a clear indicator of correlation but rather an upstream-downstream relationship derived from data changes. Therefore, further experiments are needed to confirm the findings.

In the *in vivo* experiment, we utilized CCK-8, flow cytometry, and Transwell assays to demonstrate that MRPL13 knockout effectively reduced cell proliferation, increased the number of cells in the nondividing phase, and attenuated cell migration. Our findings are consistent with those of previous studies, further substantiating the positive impact of MRPL13 on tumor cell proliferation and metastasis [45]. Decreasing MRPL13 expression may therefore be a promising strategy to inhibit tumor initiation and progression.

Our research is also insufficient because pan-cancer analysis involves a large number of cancer types. The results of bioinformatics analysis are slightly different, and even contradictory explanations appear in different cancer types. In addition, in the experimental verification, due to the influence of various factors, we cannot obtain all types of cancer samples, which leads to our verification only in lung adenocarcinoma. Further clinical studies are needed in the future to explore the role of MRPL 13 in tumor diagnosis, prognosis and treatment.

In conclusion, this study provides the first comprehensive analysis of the role of MRPL13 in pan-cancer development. Based on its differential expression in tumors and normal tissues, MRPL13 can serve as a diagnostic and prognostic marker for LUAD, BRCA, STAD, and HNSC, as well as gene mistranslation mutations. Additionally, MRPL13 expression correlated inversely with CD4+ T cells and macrophages in the immune microenvironment but directly with most immune genes. The expression of MRPL13 and its related genes is also directly proportional to the composition of copper death genes, suggesting a potential role in cuproptosis. Knockout of FOXM1 resulted in altered MRPL13 expression, indicating the possible involvement of FOXM1 in MRPL13 regulation. Single-cell data further confirmed the positive association between MRPL13 expression and various cancer cell states, including the cell cycle, invasion, metastasis, and proliferation. Moreover, *in vitro* experiments confirmed that MRPL13 knockout reduced cell proliferation, arrested cells in nondividing phases, altered EMT protein expression, and reduced cell migration, supporting the potential of MRPL13 as a therapeutic target for LUAD. Nonetheless, further research is necessary to investigate the molecular pathways and functions of MRPL13 in cancer.

## Supplementary Material

Supplementary Figures

Supplementary Tables

Supplementary Material 1

Supplementary Material 2

Supplementary Materials 3-6

Supplementary Material 7

Supplementary Material 8

Supplementary Material 9

Supplementary Material 10
