# Supplementary material for: Multi-omics analysis of MRPL-13 as a tumor-promoting marker from pan-cancer to lung adenocarcinoma
Source: Aging (Albany NY). 2023 Oct 12;15(19):10640–80. doi: 10.18632/aging.205104 (PMC10599762; doi:10.18632/aging.205104)
Supplement: Supplementary Figures [file aging-15-205104-s001.pdf]

SUPPLEMENTARY FIGURES

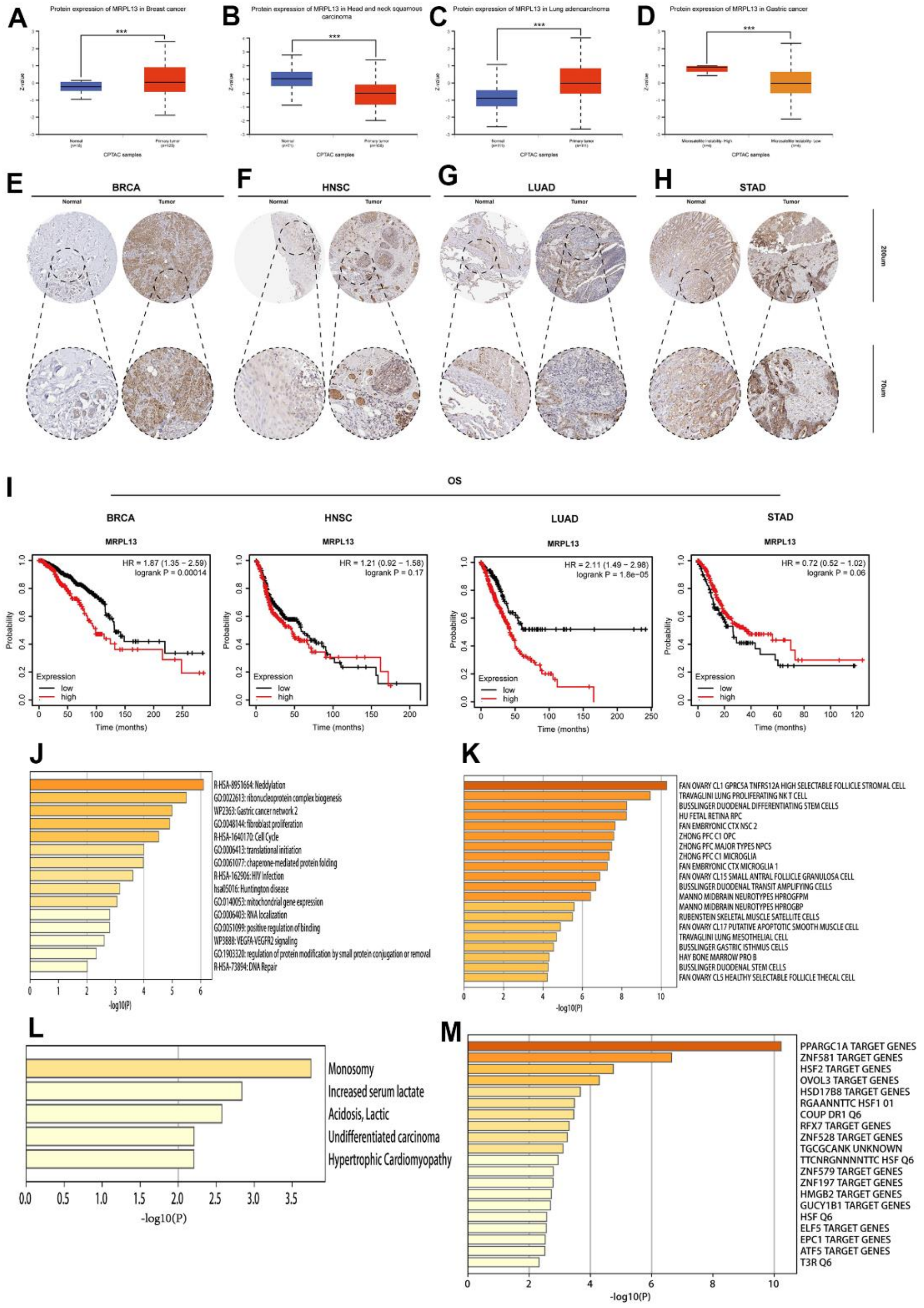

Supplementary Figure 1. Representative immunohistochemical staining (IHC) shows the differences in the expression of MRPL13 between tumor samples and normal samples in HPA database and verification the relationship between MRPL13 expression and prognosis of pan-cancer in GEO. (**A**–**C**) Comparison of MRPL13 protein expression between tumor and normal samples. (**D**) Comparison of MRPL13 protein expression between Microsatellite Instability-High and Microsatellite Instability-Low samples. (**E**–**H**) Representative immunohistochemical staining (IHC) images of pan-cancer in HPA database. (**I**) Kaplan-Meier analysis verifies the relationship between MRPL13 expression and OS in GEO database. (**J**–**M**) Enrichment analysis results of MRPL13 expression-related genes in BRCA, LUAD, HNSC and STAD. *p < 0.05, **p < 0.01, ***p < 0.001.

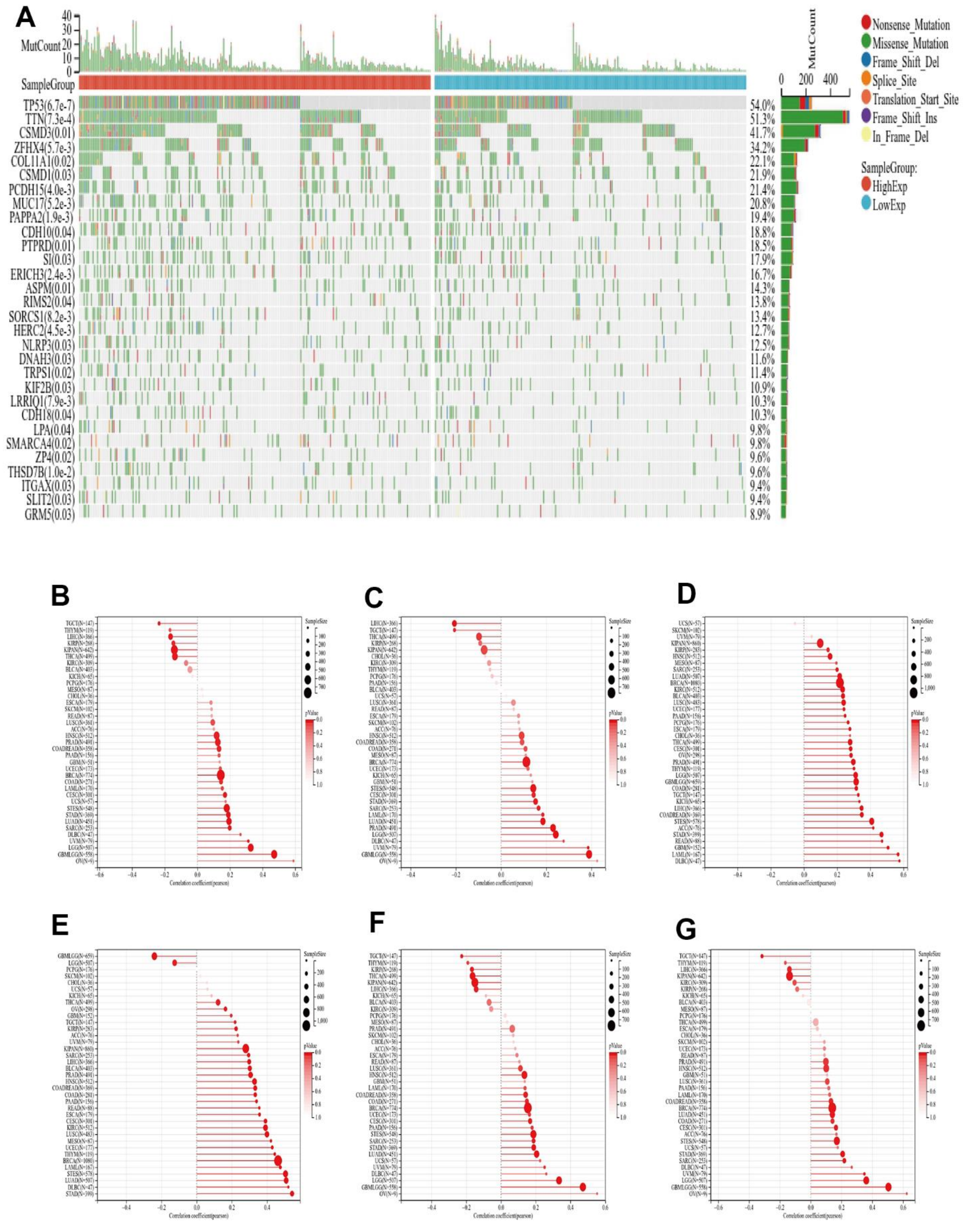

Supplementary Figure 2. Relationship between MRPL13 expression and somatic mutation and tumor dryness score. (**A**) Somatic mutation in high and low MRPL13 expression groups. (**B**–**G**) Correlation between MRPL13 expression and tumor dryness score (DNAss, EREG-METHss, DMPss, ENHss, RNAss, EREG.EXPss).

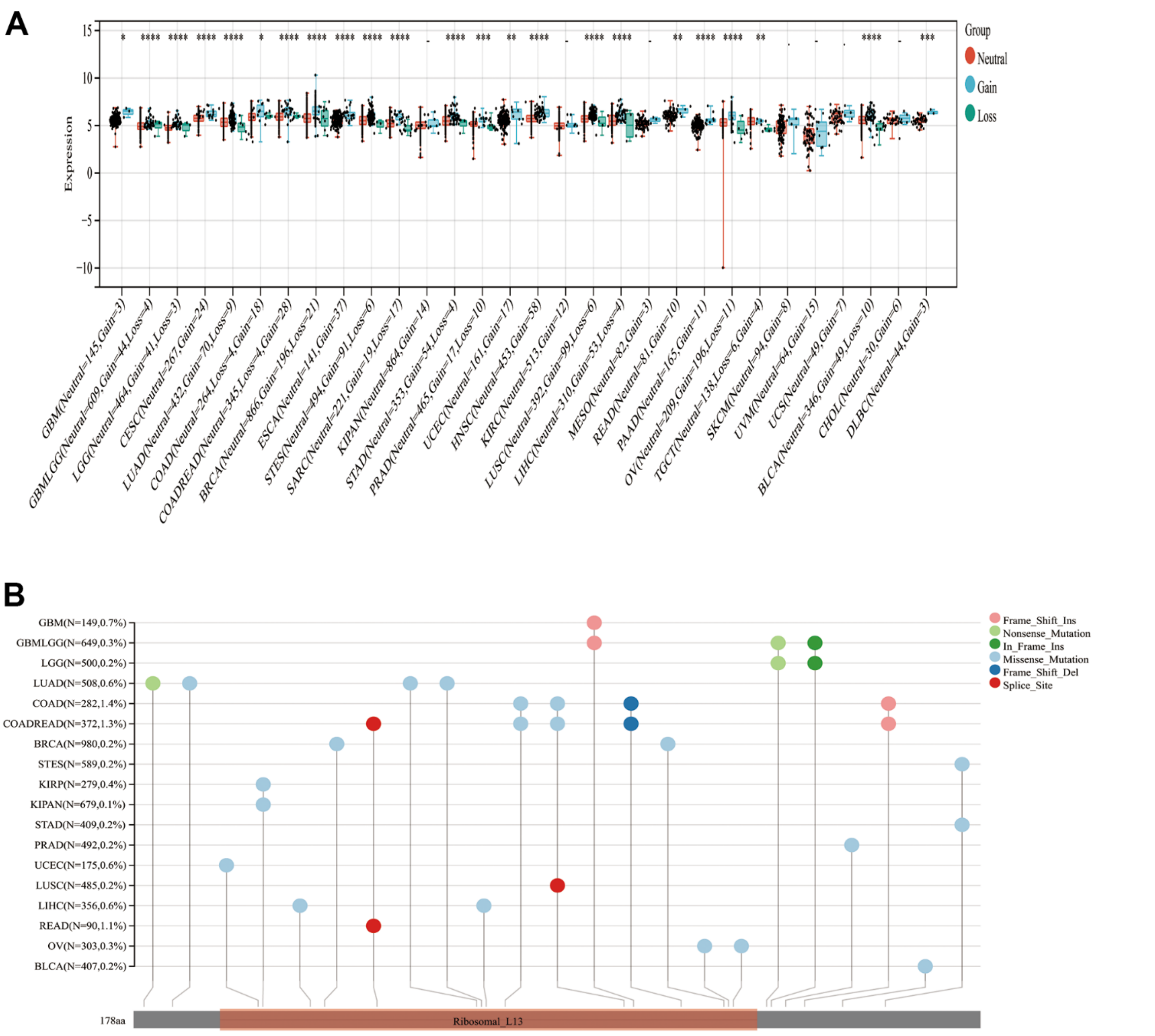

Supplementary Figure 3. The relationship between genetic alterations of MRPL13 and its expression in general cancer. (**A**) MRPL13 mutation and gene expression in pan-cancer. (**B**) The mutation of MRPL13 in individual tumors.

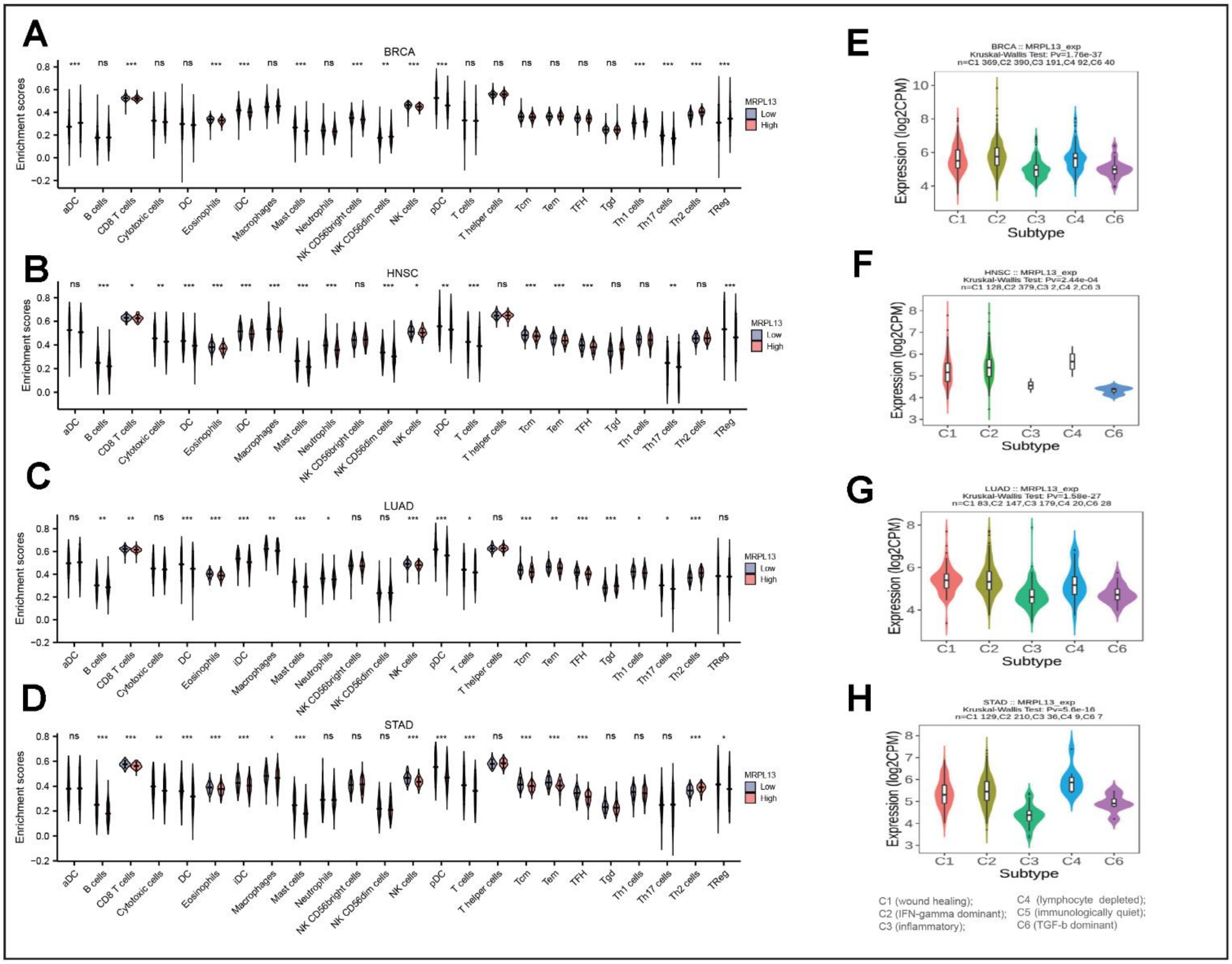

Supplementary Figure 4. Relationship between MRPL13 expression and immune cell infiltration and immune subtypes. (**A**–**D**) The infiltration of immune cells according to the high and low expression of MRPL13 in BRCA, LUAD, HNSC and STAD. (**E**–**H**) TISIDB analysis showed the association between MRPL13 expression level and 5 immune subtypes.

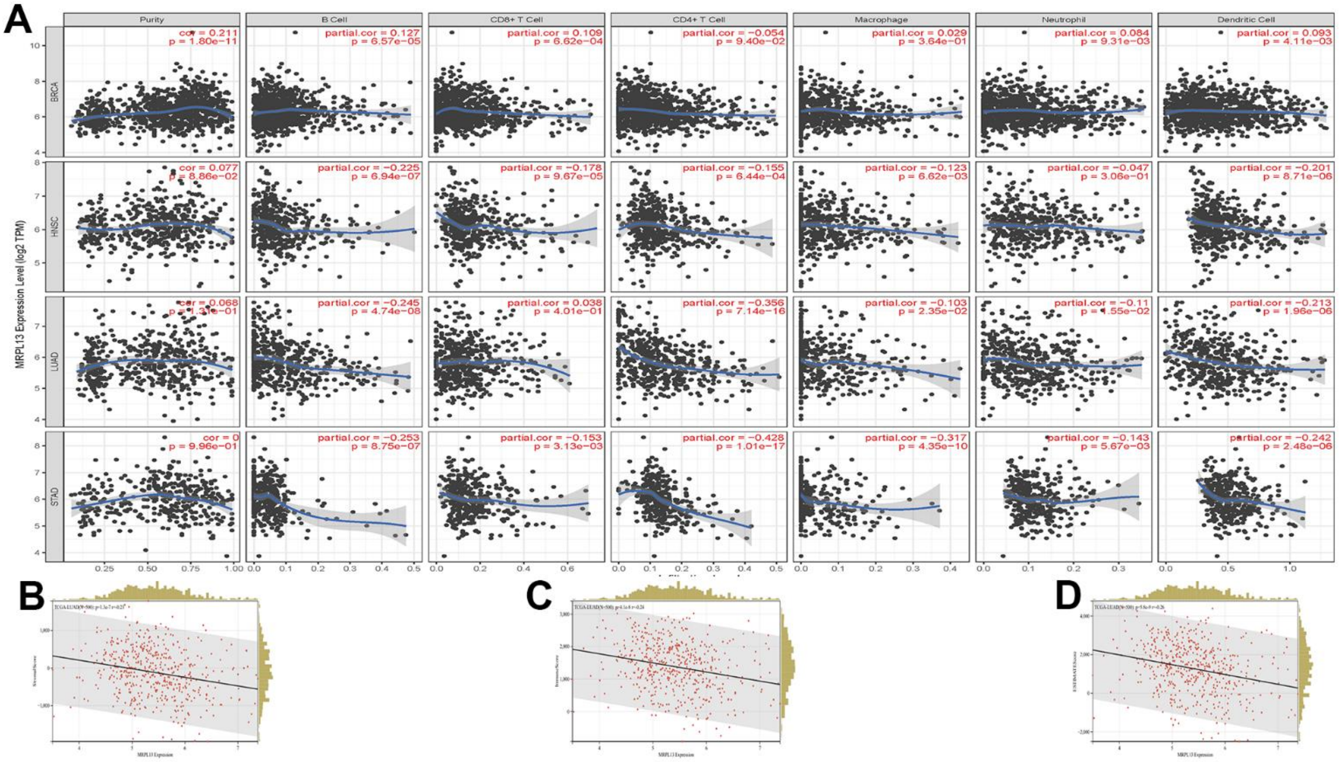

Supplementary Figure 5. Immune-associated infiltration with MRPL13. (**A**) Analysis of immune-associated cells infiltration with MRPL13 expression in BRCA, LUAD, HNSC and STAD. (**B**–**D**) The relationship between Stromal, Immune and ESTIMATE Scores with MRPL13. p ≥ 0.05; *p < 0.05; **p < 0.01; ***p < 0.001.

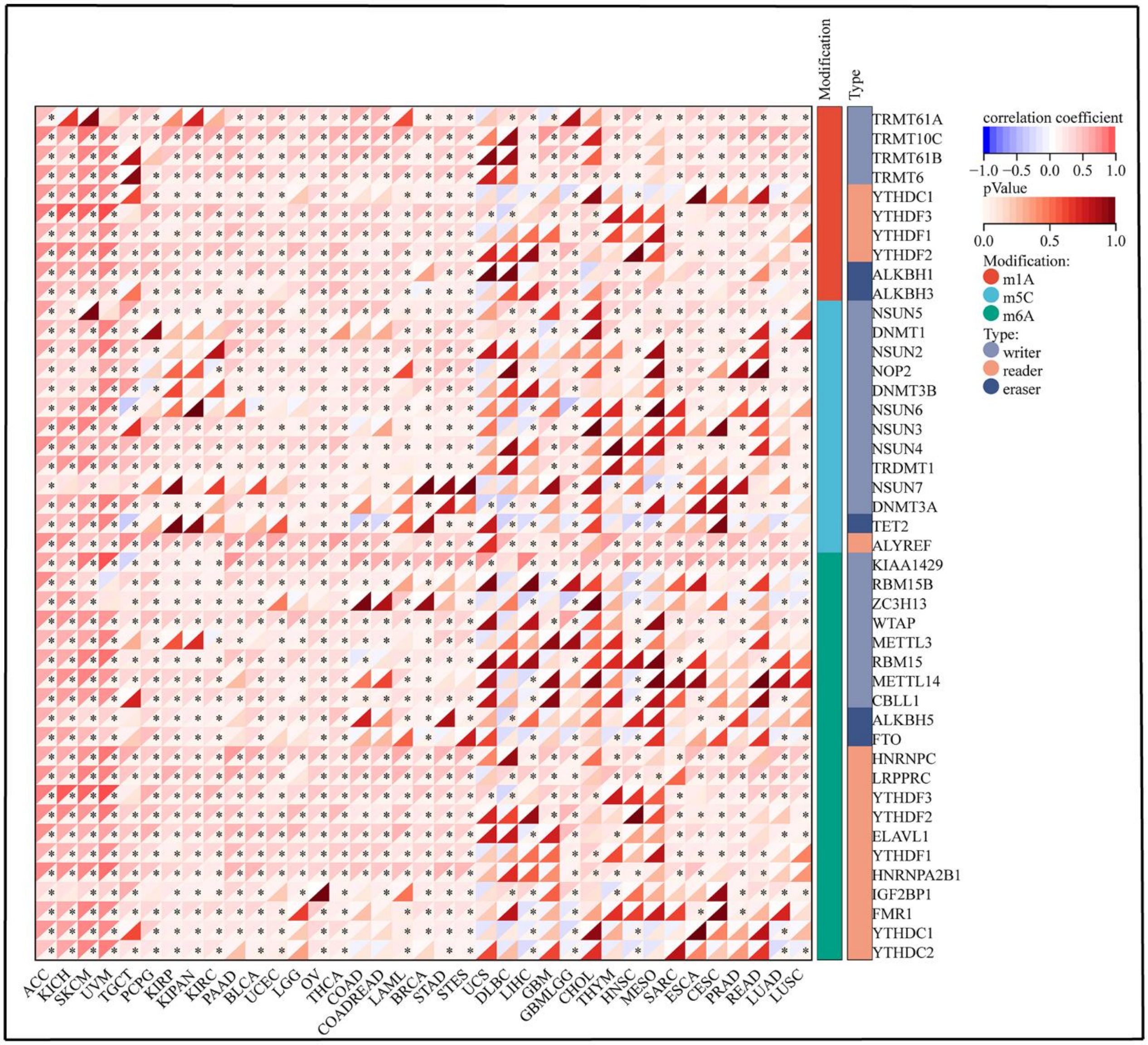

Supplementary Figure 6. Correlation of RNA modifications (m1A, m5C, and m6A) and MRPL13 in different types of cancers. *p-value < 0.05.

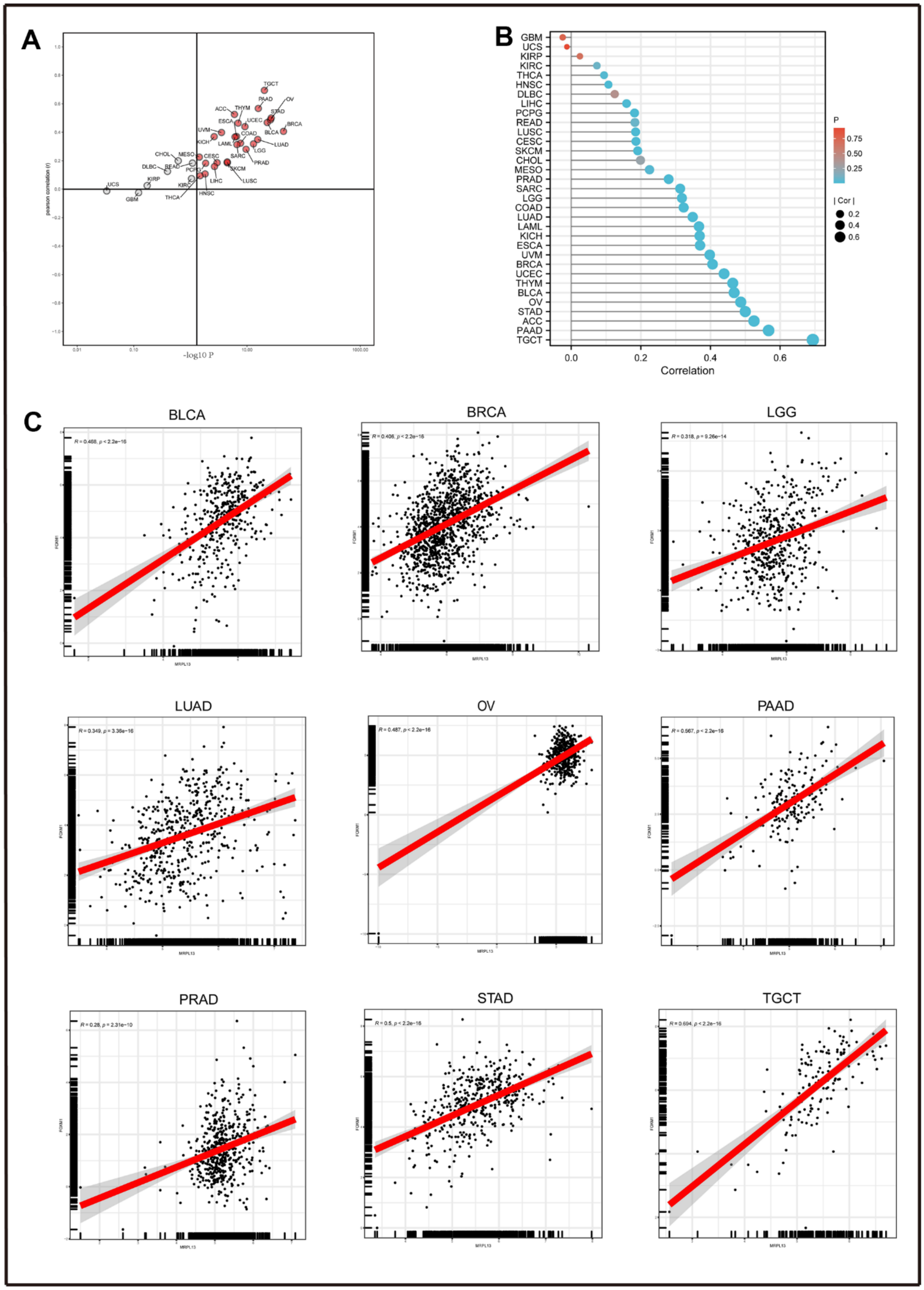

Supplementary Figure 7. The result of querying candidate causal perturbations of MRPL13. (**A**) Pearson correlation between MRPL13 and its upstream disturbance factors FOXM1 in pan-cancer. (**B**) Heatmaps show the correlation between MRPL13 and upstream disturbance factor FOXM1 in pan-cancer. (**C**) MRPL13 is positively correlated to FOXM1 in BLCA, BRCA, LGG, LUAD, OV, PAAD, PRAD, STAD, TGCT.
