# Supplementary material for: Multi-omics analysis of MRPL-13 as a tumor-promoting marker from pan-cancer to lung adenocarcinoma
Source: Aging (Albany NY). 2023 Oct 12;15(19):10640–80. doi: 10.18632/aging.205104 (PMC10599762; doi:10.18632/aging.205104)
Supplement: Supplementary Tables [file aging-15-205104-s002.pdf]

SUPPLEMENTARY TABLES

Supplementary Table 1. Comparison of clinical characteristics of patients with high and low MRPL13 expression.

Characteristic	Low expression of MRPL13	High expression of MRPL13	p
N	267	268	
Age, n (%)			0.095
<=65	119 (23.1%)	136 (26.4%)	
>65	142 (27.5%)	119 (23.1%)	
Gender, n (%)			0.090
Female	153 (28.6%)	133 (24.9%)	
Male	114 (21.3%)	135 (25.2%)	
Race, n (%)			0.565
Asian	4 (0.9%)	3 (0.6%)	
Black or African American	32 (6.8%)	23 (4.9%)	
White	204 (43.6%)	202 (43.2%)	
T stage, n (%)			< 0.001
T1	111 (20.9%)	64 (12%)	
T2	123 (23.1%)	166 (31.2%)	
T3	22 (4.1%)	27 (5.1%)	
T4	9 (1.7%)	10 (1.9%)	
N stage, n (%)			0.008
N0	187 (36%)	161 (31%)	
N1	41 (7.9%)	54 (10.4%)	
N2	27 (5.2%)	47 (9.1%)	
N3	0 (0%)	2 (0.4%)	
M stage, n (%)			0.296
M0	162 (42%)	199 (51.6%)	
M1	8 (2.1%)	17 (4.4%)	
Pathologic stage, n (%)			0.015
Stage I	164 (31.1%)	130 (24.7%)	
Stage II	56 (10.6%)	67 (12.7%)	
Stage III	34 (6.5%)	50 (9.5%)	
Stage IV	9 (1.7%)	17 (3.2%)	
Primary therapy outcome, n (%)			0.175
PD	29 (6.5%)	42 (9.4%)	
SD	18 (4%)	19 (4.3%)	
PR	3 (0.7%)	3 (0.7%)	
CR	182 (40.8%)	150 (33.6%)	
Smoker, n (%)			0.206
No	43 (8.3%)	32 (6.1%)	
Yes	217 (41.7%)	229 (44%)	
OS event, n (%)			< 0.001
Alive	196 (36.6%)	147 (27.5%)	
Dead	71 (13.3%)	121 (22.6%)	
Age, median (IQR)	67 (59, 73)	65 (58.5, 71)	0.078

Supplementary Table 2. Clinical characteristics of patients with tumor samples participating in immunohistochemistry.

Characteristics	
N	50
Age, n (%)	
<=65	19
>65	31
Age, median (IQR)	69.2
Gender	
Female	32
Male	18
T stage	
T1	26
T2	18
T3	4
T4	2
N stage	
N0	43
N1	7
N2	0
N3	0
M stage	
M0	50
M1	0
Pathologic stage	
Stage I	32
Stage II	12
Stage III	6
Stage IV	0
Smoker	
No	39
Yes	11
OS event	
Alive	46
Dead	4
