# Supplementary material for: Multi-omics analysis of MRPL-13 as a tumor-promoting marker from pan-cancer to lung adenocarcinoma
Source: Aging (Albany NY). 2023 Oct 12;15(19):10640–80. doi: 10.18632/aging.205104 (PMC10599762; doi:10.18632/aging.205104)
Supplement: Supplementary Materials 3-6 [file aging-15-205104-s005.pdf]

Supplementary Material 3. Experimental details of qRT–PCR assay, Western blotting analysis, immunohistochemistry (IHC) staining assay, cell viability assay, Transwell assay and flow cytometry assay.

qRT-PCR assay

Total RNA was extracted using the RNA-Quick Purification kit. cDNA Synthesis reagent was used to reverse transcribe RNA into cDNA. Real-time quantitative PCR (RT-qPCR) was done using the Roche Light Cycler 480 Detection System. All reagents are used according to the manufacturer's instructions. In this study, the relative expression of the MRPL13 were normalized to GAPDH levels, and the relative gene expression was determined by the $2^{-\Delta\Delta CT}$ method. The primers in qPCR were as follows: GAPDH forward primer: GGTGAAGGTCGGAGTCAAC. GG, and reverse primer: GAGGTCAATGAAGGGGTCATTG; MRPL13 forward primer: CTTCGCATACTGGCTACC, reverse primer: CTTCTGGAATATACTCATCTGG.

Western blotting analysis

Protein in tissue sample was extracted by the mixture of RIPA buffer and PMSF (100:1). The protein concentration of the samples was determined by the BCA protein assay kit. After sodium dodecyl sulfate-polyacrylamide gel electrophoresis, protein was transferred to polyvinylidene fluoride membrane (280 mA, 2 h). After sealing with 5% skim milk for 2 hours, the membrane was incubated with the primary antibody at 4 C overnight. The second antibody was incubated at room temperature (22–25 C) for 2 hours. Reactive protein bands were found using enhanced chemiluminescence (ECL), and the intensity of these membranes was determined using ChemiDoc™ MP Imaging System (Bio-Rad, USA). The antibodies used are as follows: MRPL13 (1:10000, ab190232, Abcam, Cambridge, UK).

Immunohistochemistry (IHC) staining assay

LUAD samples were taken from 50 patients who underwent surgical treatment in Zhejiang People's Hospital from 2020 to 2022. In short, tissue sections were incubated with antibodies against MRPL 13 (10 g/ml, Abcam) overnight, then with secondary antibodies, then stained with DAB, and finally counterstained with hematoxylin.

Cell viability assay

According to the manufacturer's suggestion, CCK-8 reagent was used to test the effect of MRPL13 gene knockout on LUAD cell viability. H1975 cells in the experimental group and the control group were inoculated into 96-well plates at a cell density of 5×103 per well. After 12 hours, it was changed to a medium containing 10% CCK-8 reagent, incubated in the dark, and then the absorbance at 450 nm was measured by an enzyme-labeled instrument (Synergy LX, BioTek, USA).

Transwell assay

We used Transwell membrane to detect the invasion and migration of LUAD cells. LUAD cells were digested with trypsin, and the same number of cells were inoculated in the upper layer of 24-well chamber with serum-free medium, and the lower layer was the control medium containing 15% serum. At the same time, during the invasion experiment, a layer of Matrigel matrix (Corning, USA) was laid on the bottom of the chamber (0.1ml/ 24-well plate per hole (the concentration is 200-300 μg/ mL)), and after 24 hours of culture at 37° C and 5% CO_2, the cells in the chamber were fixed with 4% formaldehyde, stained with 1% crystal violet, and the cells in the upper layer of the chamber were wiped with cotton swabs. Under the microscope, the number of cells entering the chamber was counted in five random areas to evaluate the invasion and migration ability of cells.

Flow cytometry assay

Each group of transfected cells verified by RNA was collected, and related reagents were prepared according to the manufacturer's instructions. Apoptosis was detected by Annexin V-FITC/PI double staining, and cell cycle was analyzed by PI staining. All tests were carried out on a Beckman flow machine. The pictures were visualized with FLOWJO software.

Supplementary Material 4. Intersection list of genes related to MRPL13 in BRCA, LUAD, HNSC and STAD.

DERL1
C8orf76
DCAF13
POLR2K
ENY2
ELOC
NSMCE2
TMEM70
TATDN1
MTERF3
DSCC1
NTAQ1
NUDCD1
NDUFB9
MRPL15
RBIS
C8orf33
COPS5
MED30
CYC1
CYRIB
MRPS28
EIF3H
ZFAND1
PPIL1
PTGES3
EIF3E
MAD2L1
RAN
H2AZ1
HSPE1
CACYBP
UBE2T
CKS2
BIRC5
LRR1
PSMA4
CKS1B
CCT4
TNXB

Supplementary Material 5. The list of gene mutation rates of cells with high and low expression of MRPL13.

Tag	MutCount	1
TP53	242	0.000000667
TTN	230	0.000728261
CSMD3	187	0.013909758
ZFHX4	153	0.005672077
COL11A1	99	0.022380166
CSMD1	98	0.029273059
PCDH15	96	0.004025917
MUC17	93	0.005182263
PAPPA2	87	0.001919859
CDH10	84	0.038470814

Supplementary Material 6. The comprehensive evaluation score of MRPL13 protein between the cancer tissue group and adjacent normal tissue group.

Number/ cancer	Doctor 1	Doctor 2	Doctor 3	Average		Number/normal	Doctor 1	Doctor 2	Doctor 3	Average	
1	6	6	6	6	0	1	3	3	3	3	0
2	5	5	6	5.333333333	0.471404521	2	3	4	4	3.666666667	0.471404521
3	6	6	5	5.666666667	0.471404521	3	3	4	4	3.666666667	0.471404521
4	3	2	3	2.666666667	0.471404521	4	3	3	4	3.333333333	0.471404521
5	8	7	7	7.333333333	0.471404521	5	6	5	5	5.333333333	0.471404521
6	6	7	7	6.666666667	0.471404521	6	6	6	6	6	0
7	7	6	7	6.666666667	0.471404521	7	6	6	6	6	0
8	7	7	7	7	0	8	6	6	6	6	0
9	7	7	7	7	0	9	6	7	7	6.666666667	0.471404521
10	3	3	2	2.666666667	0.471404521	10	3	2	3	2.666666667	0.471404521
11	3	3	1	2.333333333	0.942809042	11	3	3	3	3	0
12	5	6	6	5.666666667	0.471404521	12	1	2	2	1.666666667	0.471404521
13	4	5	6	5	0.816496581	13	2	3	2	2.333333333	0.471404521
14	7	7	7	7	0	14	3	3	2	2.666666667	0.471404521
15	6	8	7	7	0.816496581	15	5	5	5	5	0
16	7	7	7	7	0	16	4	4	5	4.333333333	0.471404521
17	7	7	7	7	0	17	4	3	4	3.666666667	0.471404521
18	7	7	7	7	0	18	4	3	4	3.666666667	0.471404521
19	7	7	7	7	0	19	7	7	7	7	0
20	5	6	7	6	0.816496581	20	7	7	8	7.333333333	0.471404521
21	3	3	2	2.666666667	0.471404521	21	7	7	7	7	0
22	1	2	2	1.666666667	0.471404521	22	4	5	5	4.666666667	0.471404521
23	2	2	2	2	0	23	7	6	7	6.666666667	0.471404521
24	3	2	2	2.333333333	0.471404521	24	3	4	4	3.666666667	0.471404521
25	6	7	8	7	0.816496581	25	3	4	4	3.666666667	0.471404521
26	7	7	7	7	0	26	3	4	4	3.666666667	0.471404521
27	8	7	7	7.333333333	0.471404521	27	1	2	2	1.666666667	0.471404521
28	7	7	7	7	0	28	3	3	3	3	0
29	7	6	7	6.666666667	0.471404521	29	5	6	6	5.666666667	0.471404521
30	7	6	7	6.666666667	0.471404521	30	5	6	6	5.666666667	0.471404521
31	7	6	7	6.666666667	0.471404521	31	3	5	5	4.333333333	0.942809042
32	7	7	7	7	0	32	7	6	7	6.666666667	0.471404521
33	6	6	7	6.333333333	0.471404521	33	7	6	7	6.666666667	0.471404521
34	2	3	3	2.666666667	0.471404521	34	3	4	4	3.666666667	0.471404521
35	1	2	3	2	0.816496581	35	2	2	2	2	0
36	6	4	5	5	0.816496581	36	6	7	7	6.666666667	0.471404521
37	5	6	6	5.666666667	0.471404521	37	5	6	6	5.666666667	0.471404521
38	4	5	6	5	0.816496581	38	5	6	6	5.666666667	0.471404521
39	8	7	6	7	0.816496581	39	7	8	7	7.333333333	0.471404521
40	8	7	6	7	0.816496581	40	7	9	8	8	0.816496581
41	7	8	8	7.666666667	0.471404521	41	4	5	5	4.666666667	0.471404521
42	6	8	8	7.333333333	0.942809042	42	6	7	7	6.666666667	0.471404521
43	7	8	8	7.666666667	0.471404521	43	7	7	7	7	0
44	7	8	8	7.666666667	0.471404521	44	7	7	7	7	0
45	7	9	8	8	0.816496581	45	7	7	7	7	0
46	3	2	3	2.666666667	0.471404521	46	2	3	2	2.333333333	0.471404521
47	2	2	4	2.666666667	0.942809042	47	3	3	3	3	0
48	3	4	6	4.333333333	1.247219129	48	3	3	3	3	0
49	8	6	5	6.333333333	1.247219129	49	3	3	3	3	0
50	9	7	7	7.666666667	0.942809042	50	9	8	9	8.666666667	0.471404521
