# Supplementary material for: Multi-omics analysis of MRPL-13 as a tumor-promoting marker from pan-cancer to lung adenocarcinoma
Source: Aging (Albany NY). 2023 Oct 12;15(19):10640–80. doi: 10.18632/aging.205104 (PMC10599762; doi:10.18632/aging.205104)
Supplement: Supplementary Material 7 [file aging-15-205104-s006.docx]

**Supplementary Material 7. Figure 14 invasion raw data.**


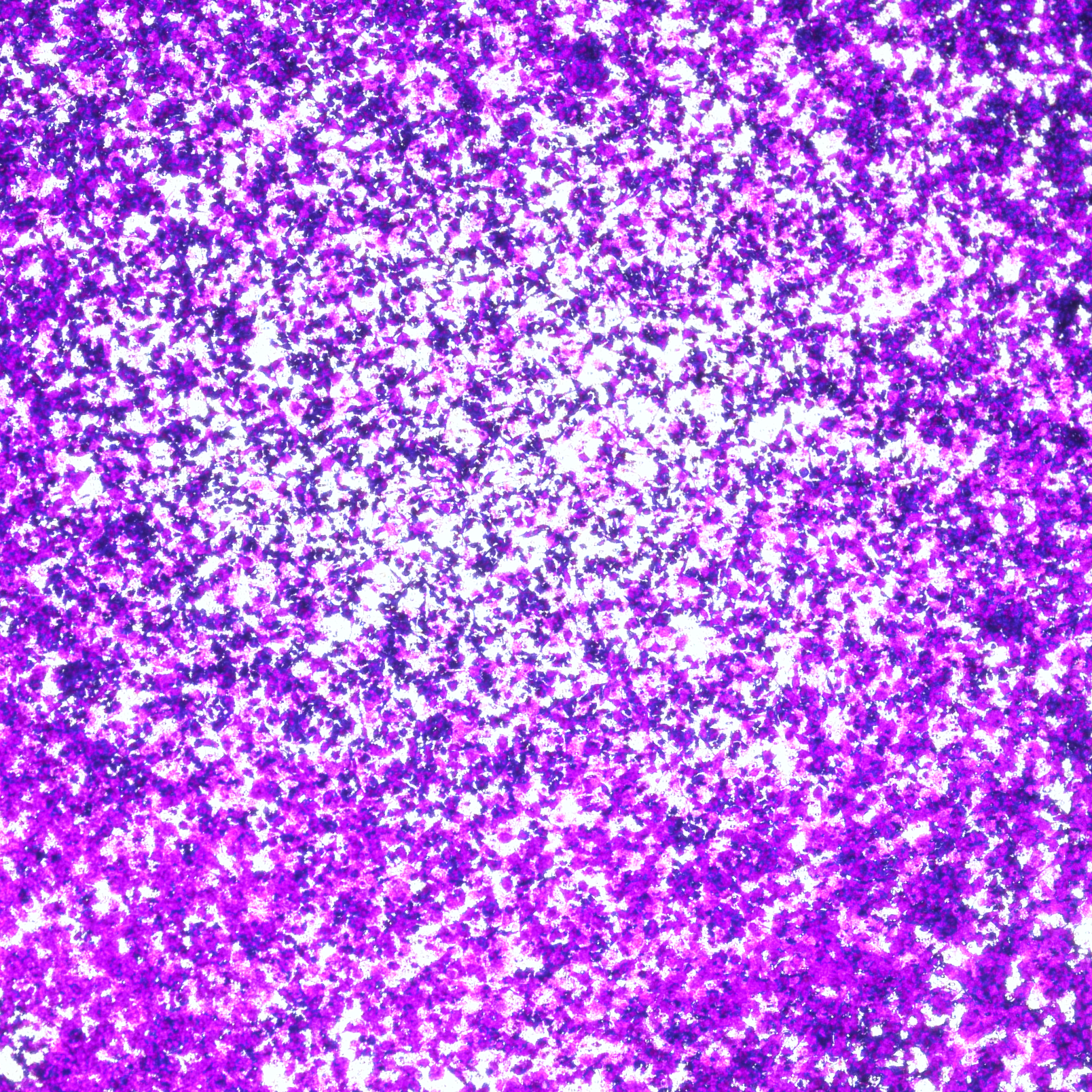


1975-100X


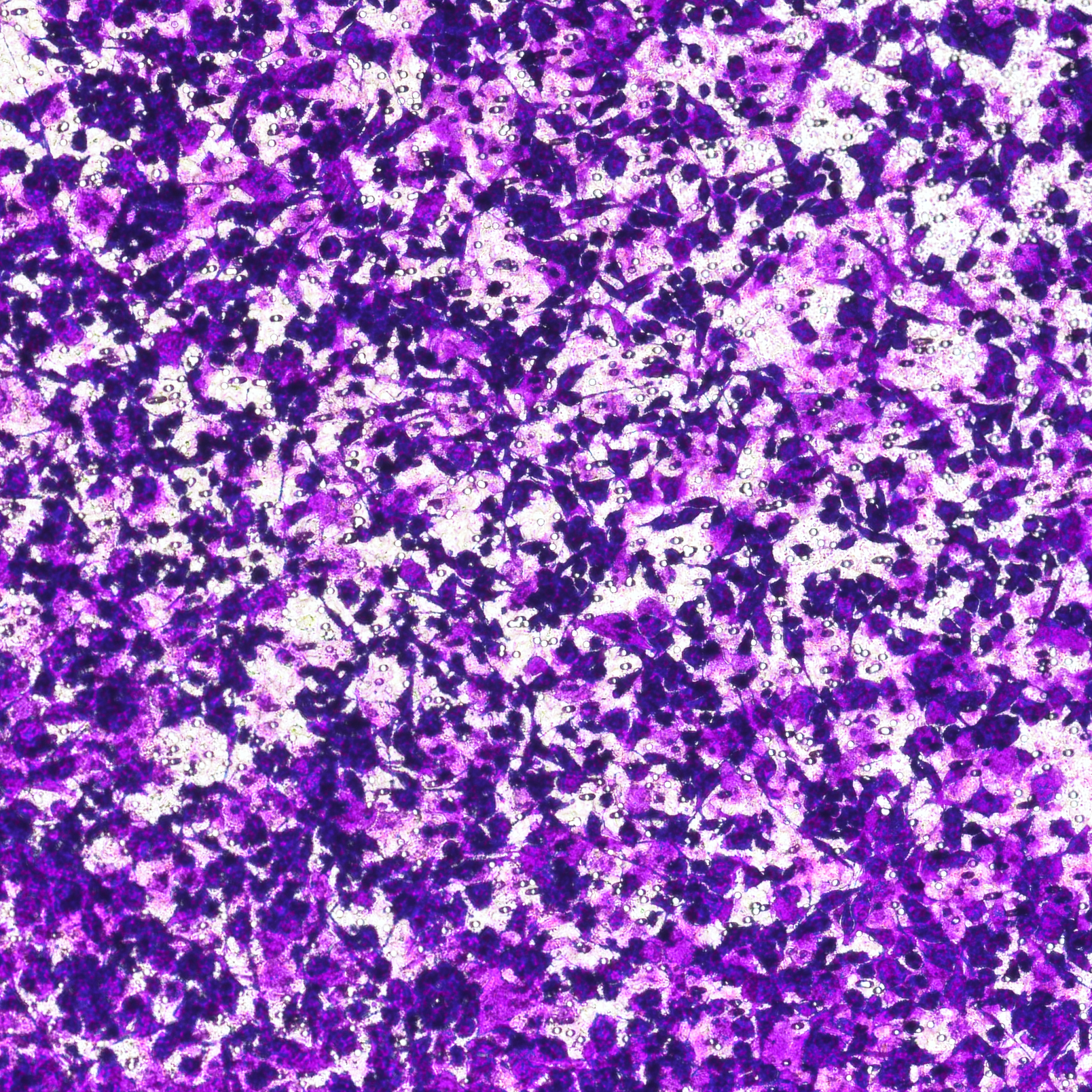


1975-250X


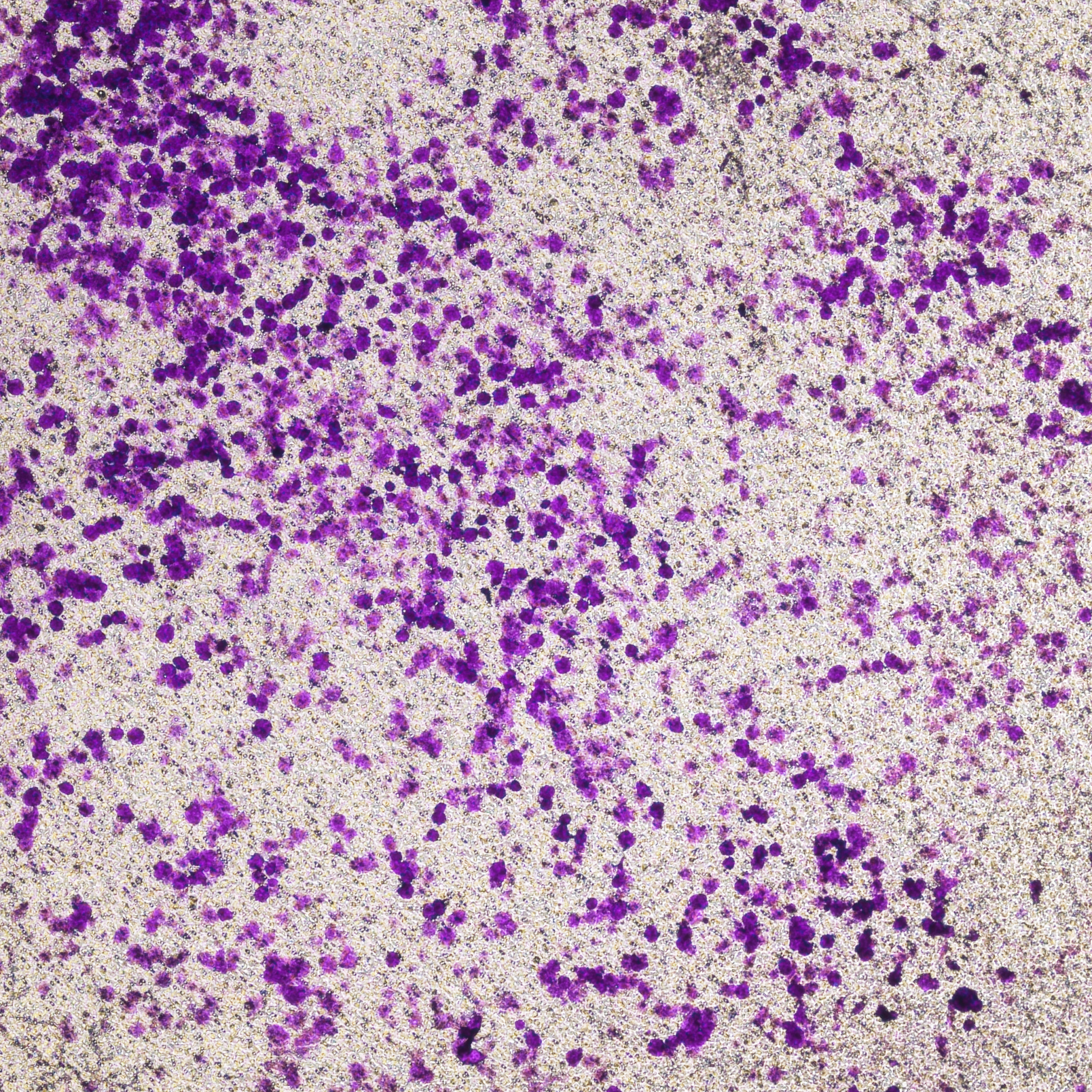


1975-MRPL13si-1-100X


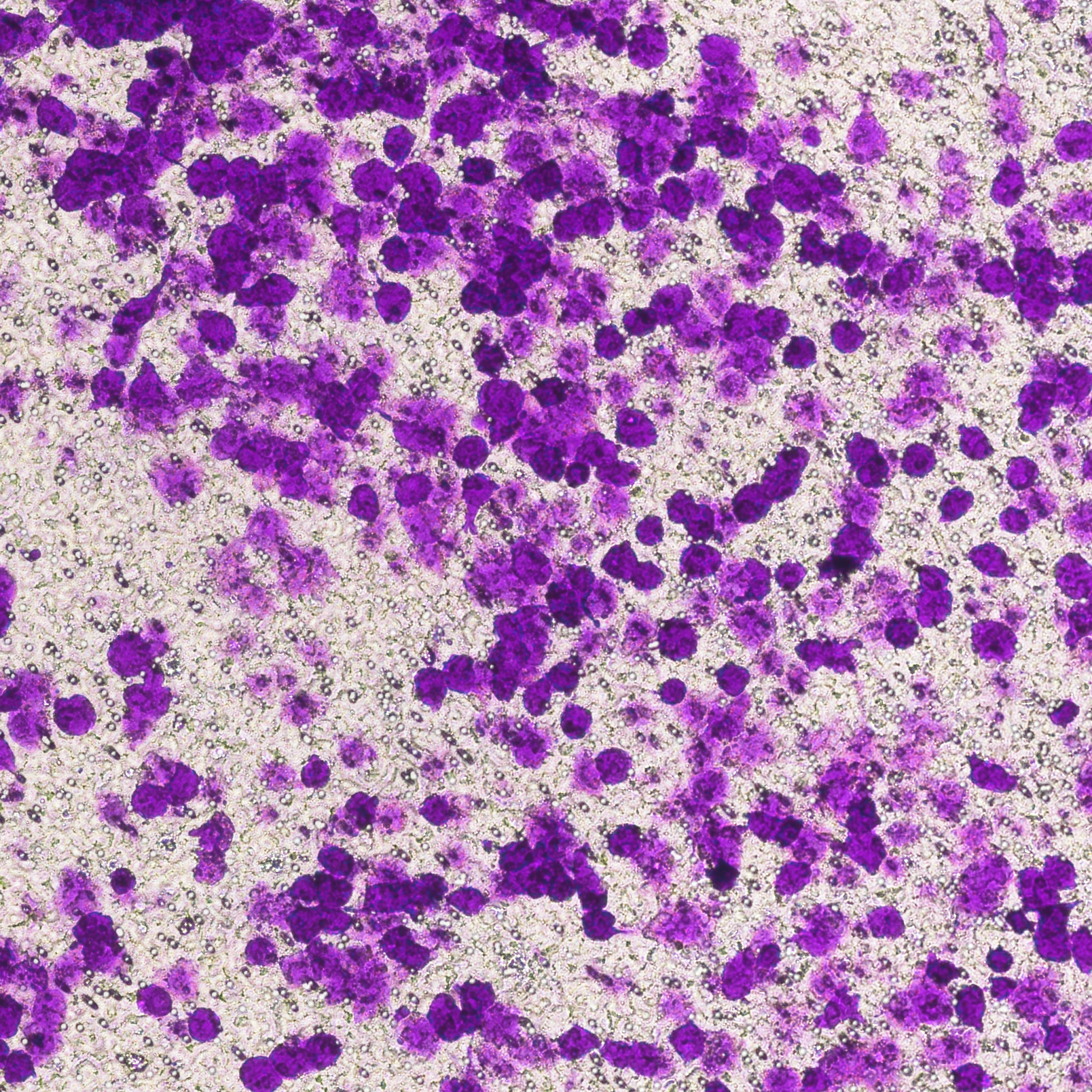


1975-MRPL13si-1-250X


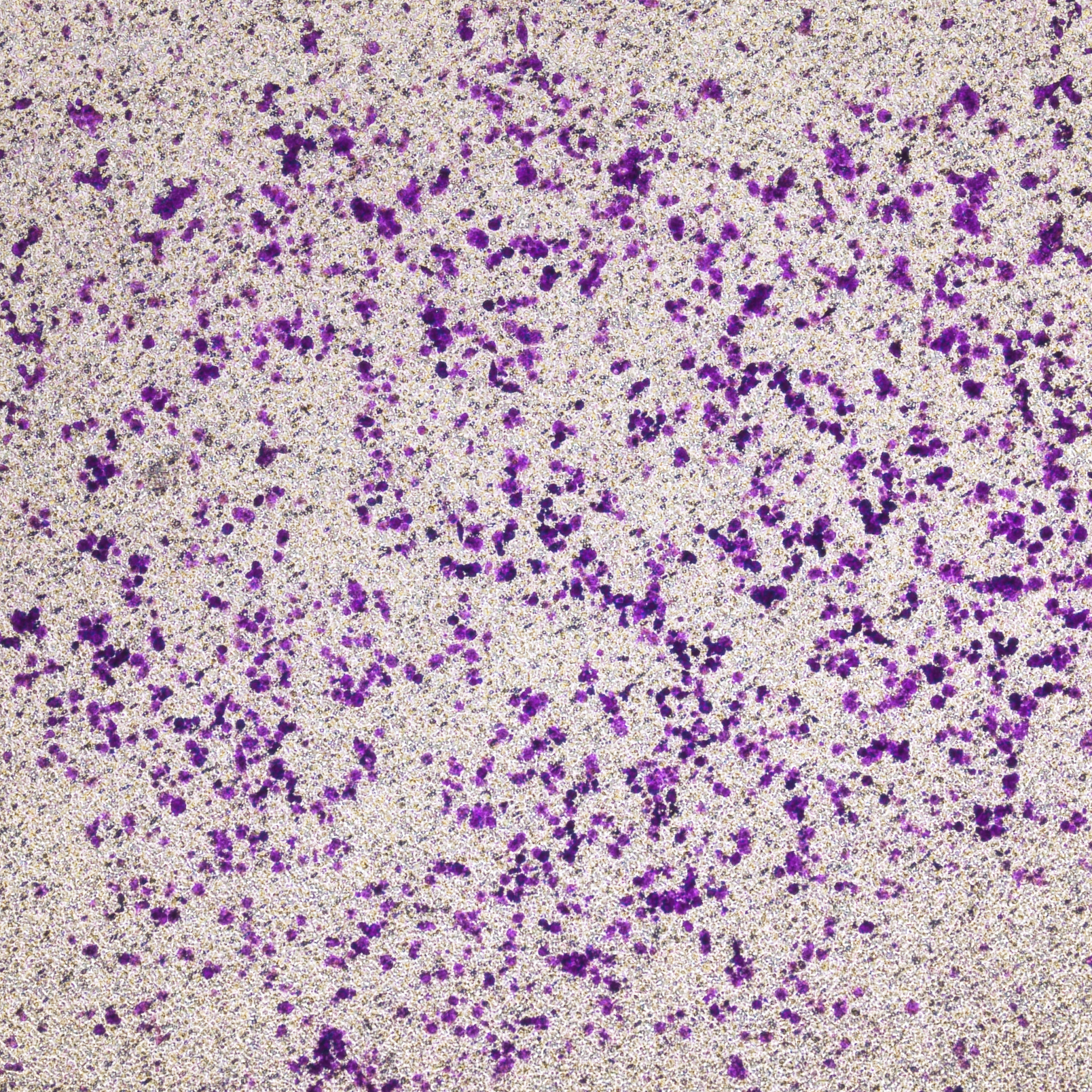


1975-MRPL13si-2-100X


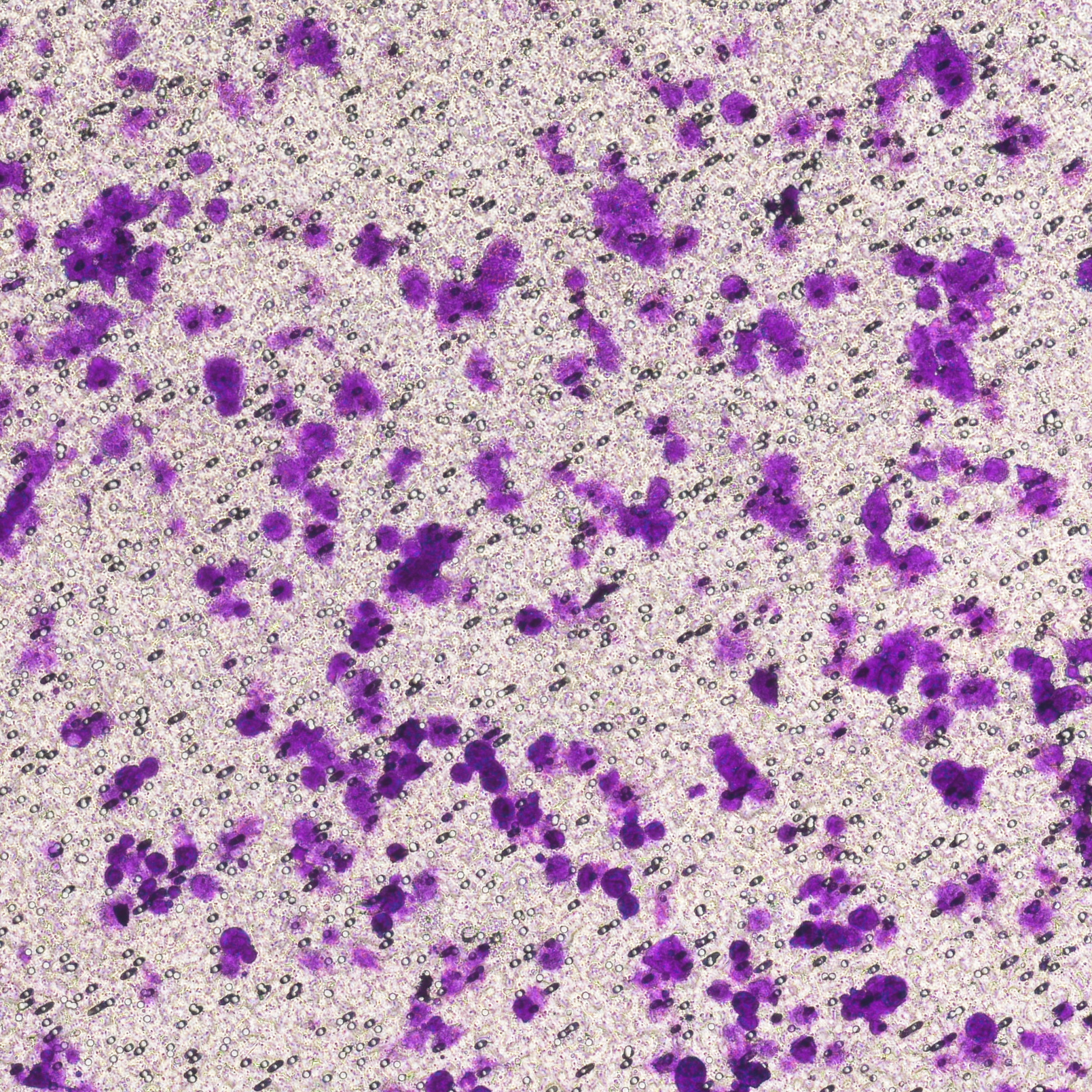


1975-MRPL13si-2-250X


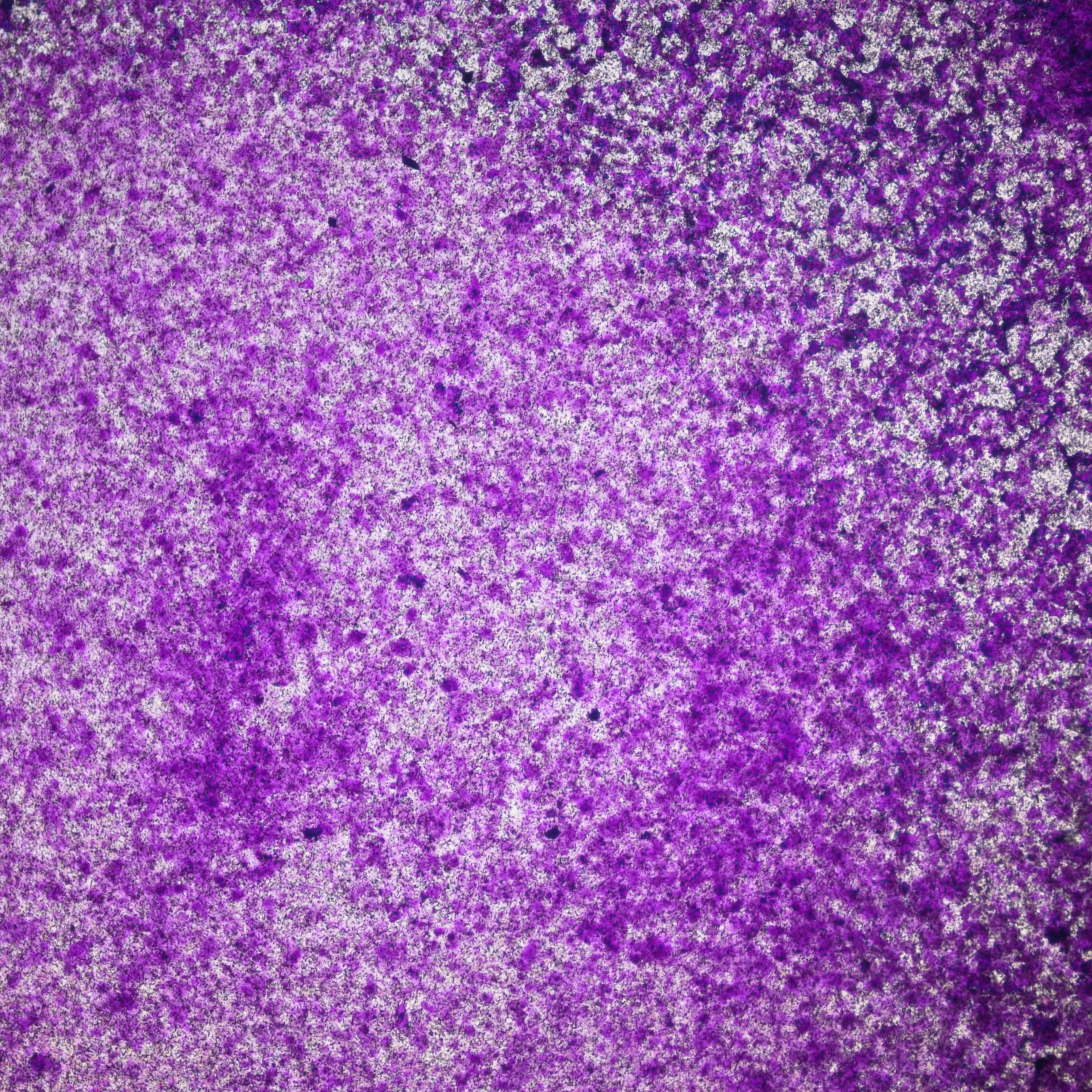


A549-100X


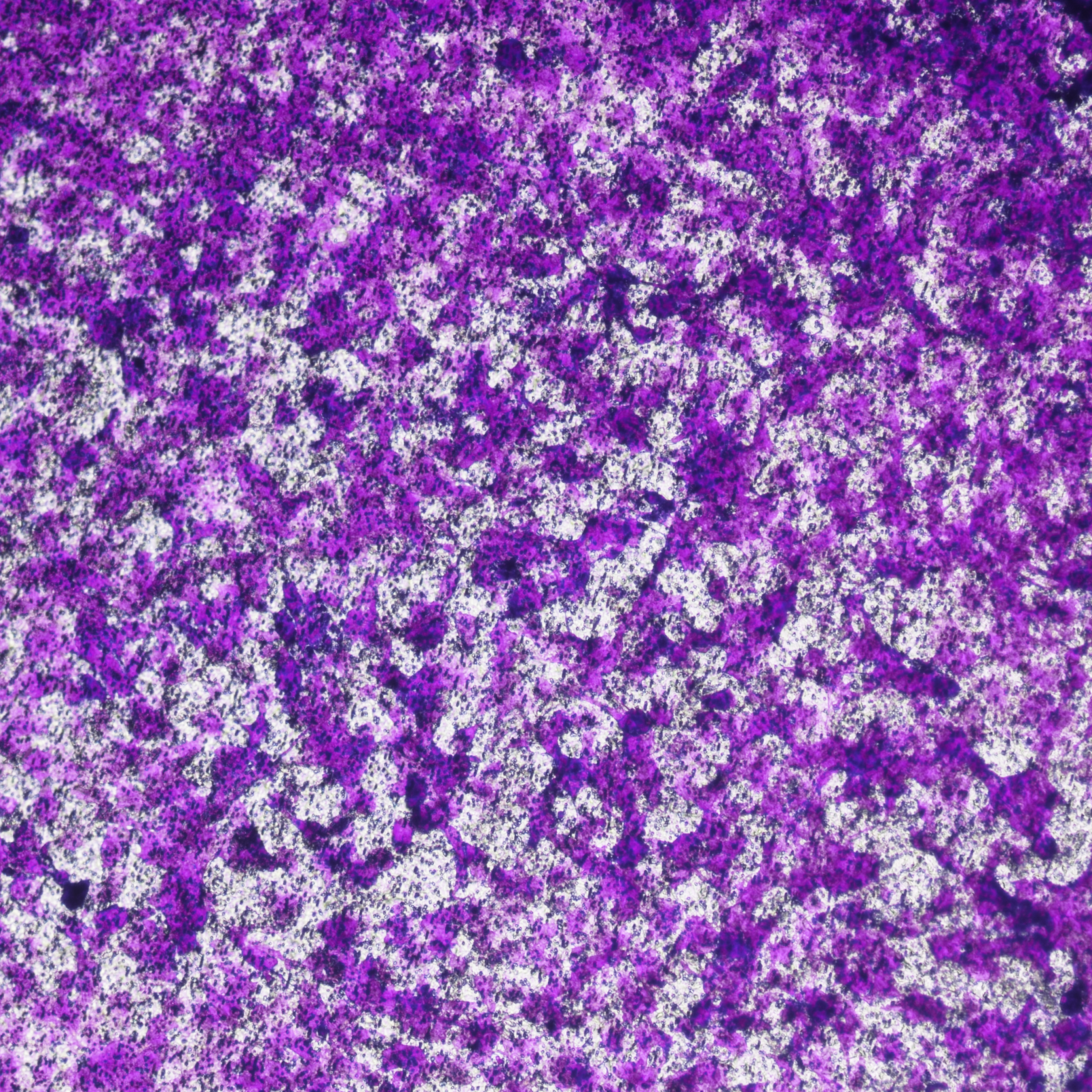


A549-250X


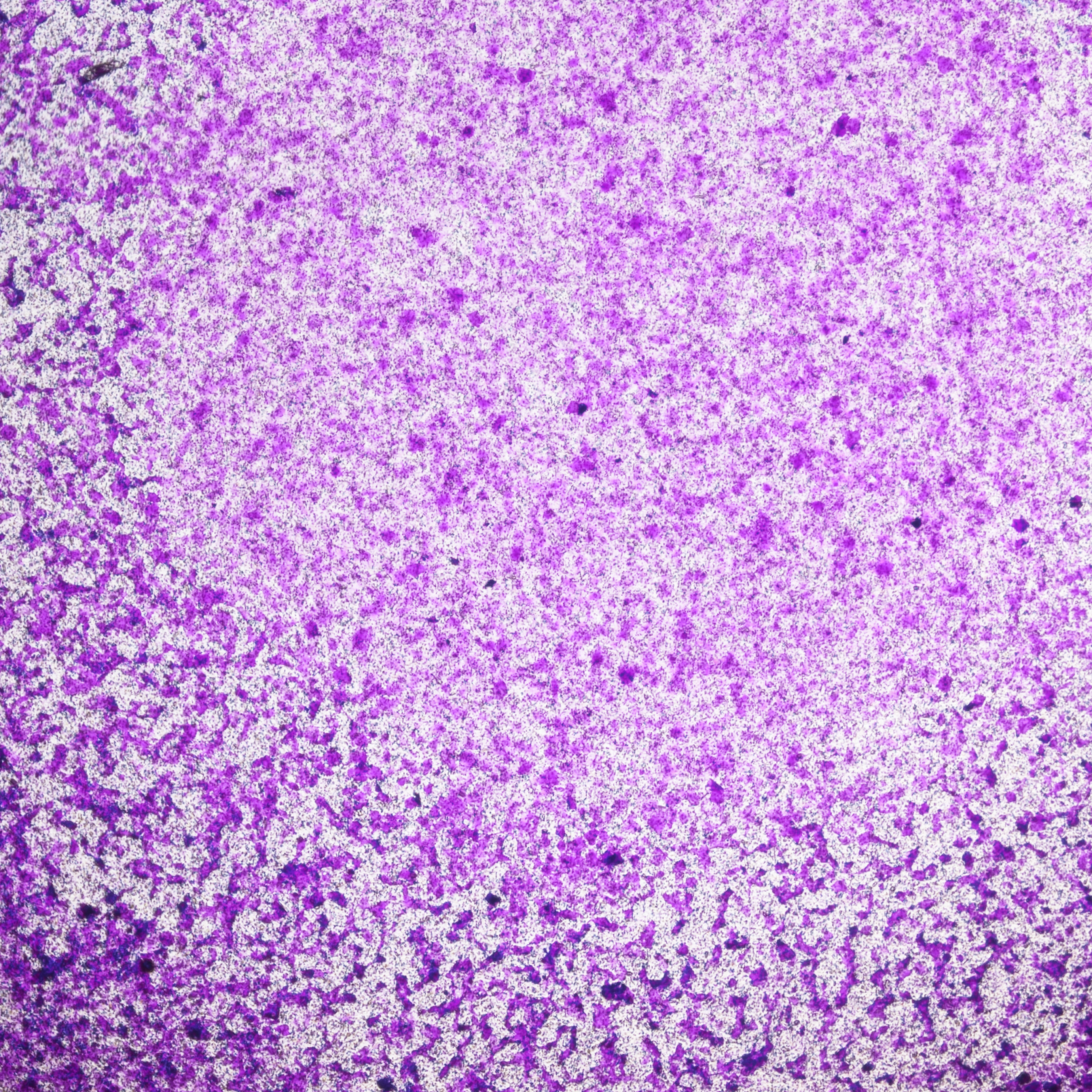


A549-MRPL13si-1-100X


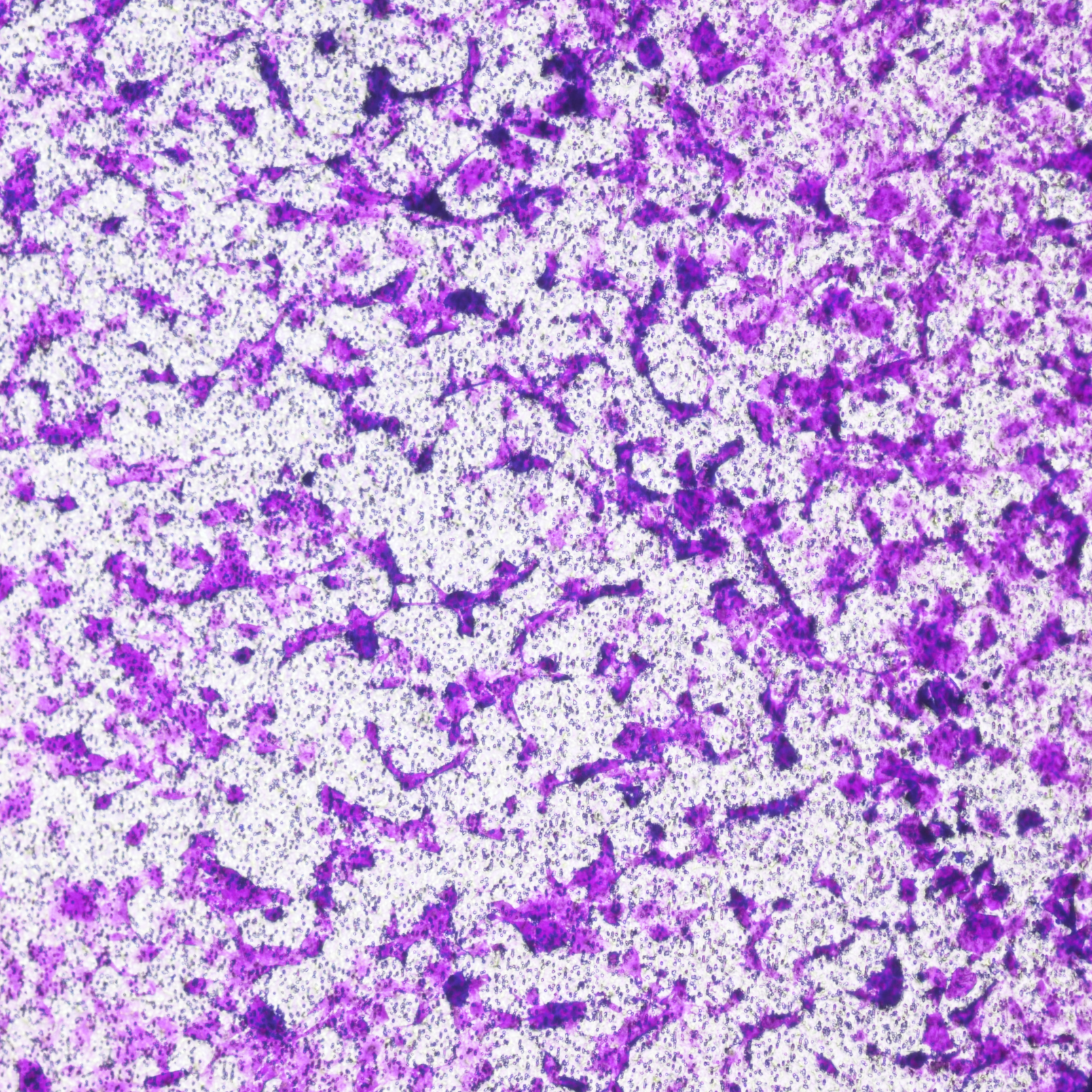


A549-MRPL13si-1-250X


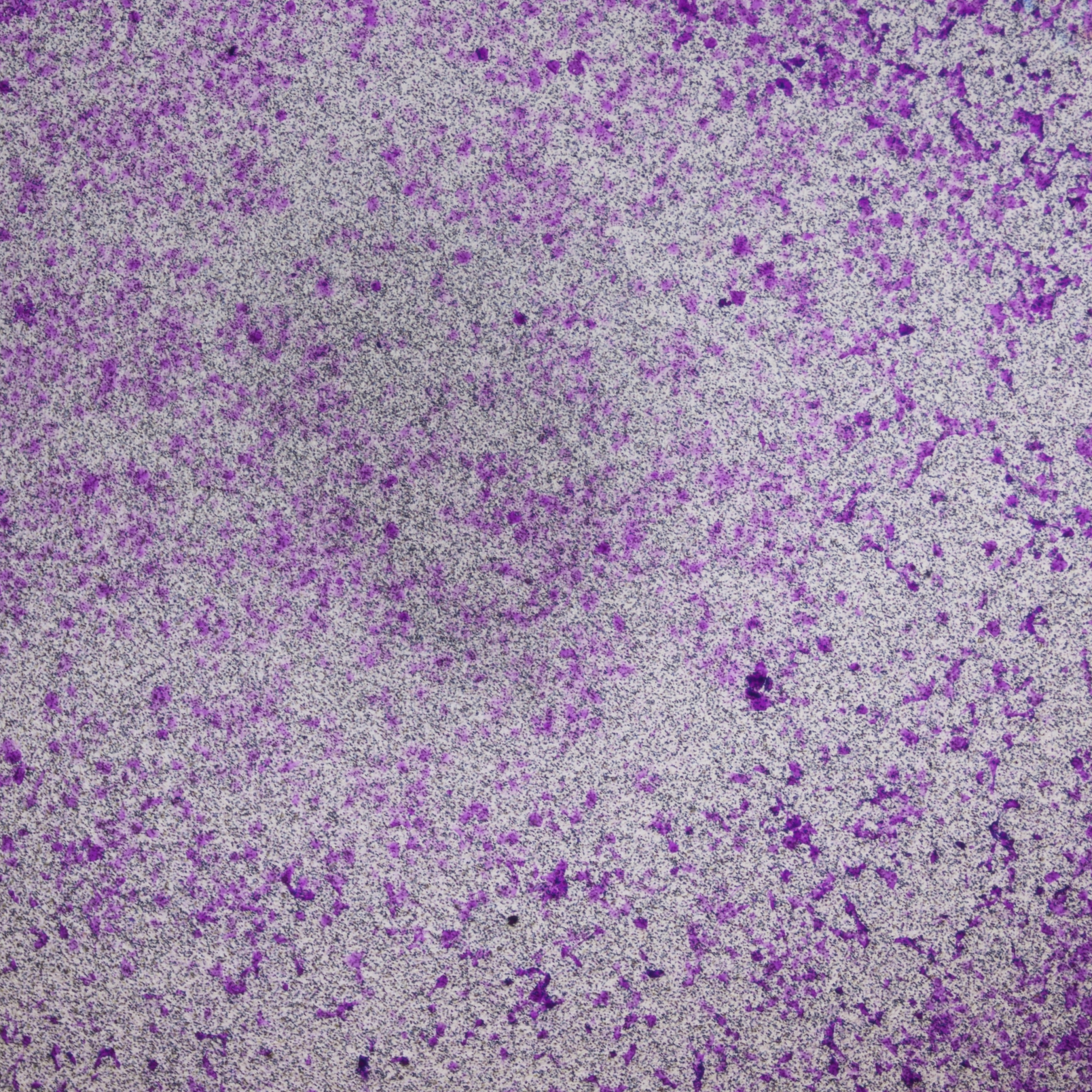


A549-MRPL13si-2-100X


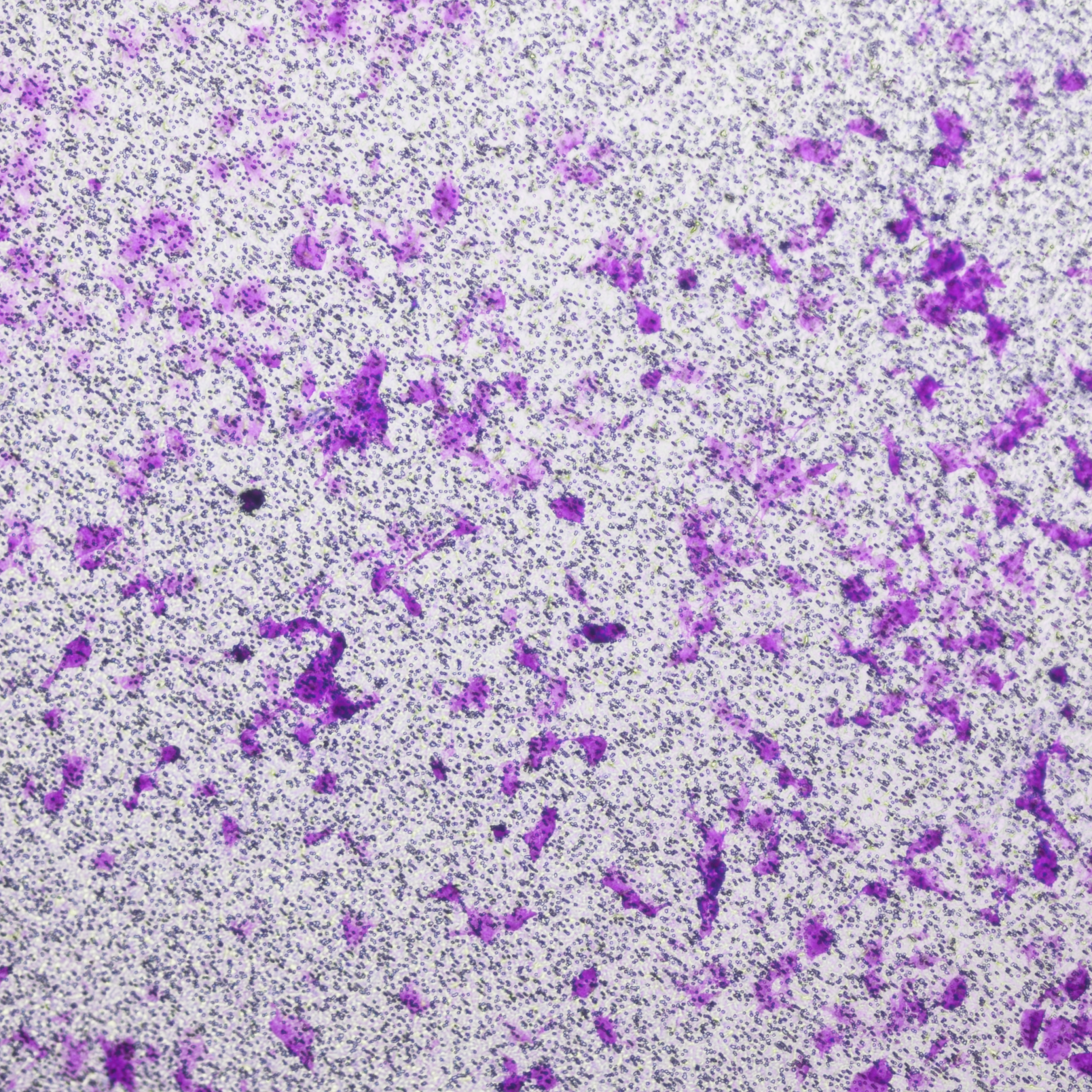


A549-MRPL13si-2-250X

| Gruop | frequency | MRPL13si-control | MRPL13si-1 | MRPL13si-2 |
| --- | --- | --- | --- | --- |
| A549-cell number | 1 | 15620 | 5699 | 6349 |
| NCI-H1975-cell-number | 1 | 18925 | 5575 | 5590 |
| A549-cell number | 2 | 14998 | 5495 | 5460 |
| NCI-H1975-cell-number | 2 | 18006 | 5517 | 6411 |
| A549-cell number | 3 | 16595 | 7738 | 6634 |
| NCI-H1975-cell-number | 3 | 17065 | 4530 | 5300 |
