# Supplementary material for: Multi-omics analysis of MRPL-13 as a tumor-promoting marker from pan-cancer to lung adenocarcinoma
Source: Aging (Albany NY). 2023 Oct 12;15(19):10640–80. doi: 10.18632/aging.205104 (PMC10599762; doi:10.18632/aging.205104)
Supplement: Supplementary Material 8 [file aging-15-205104-s007.docx]

**Supplementary Material 8. Figure 14 migration raw data.**


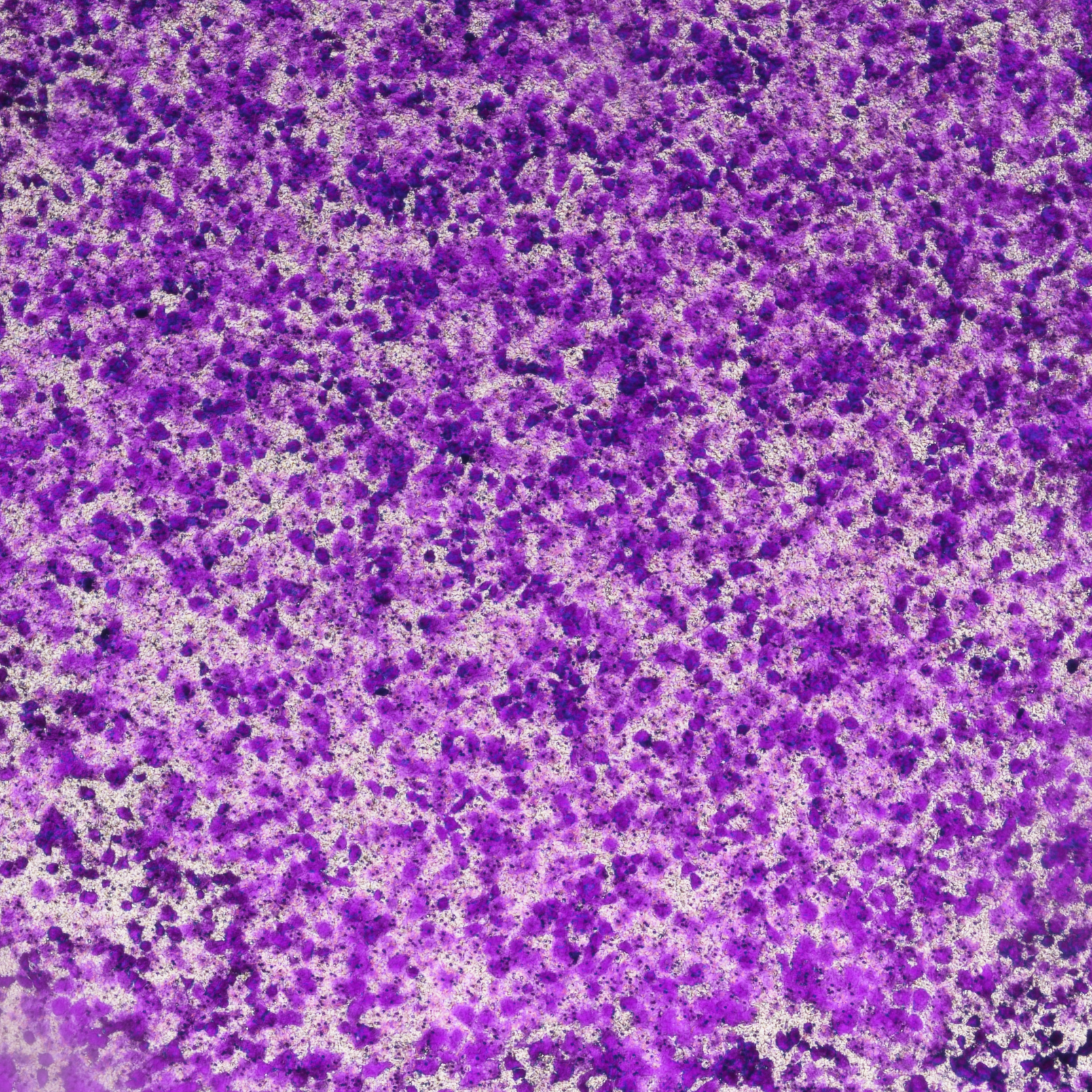


1975-100X


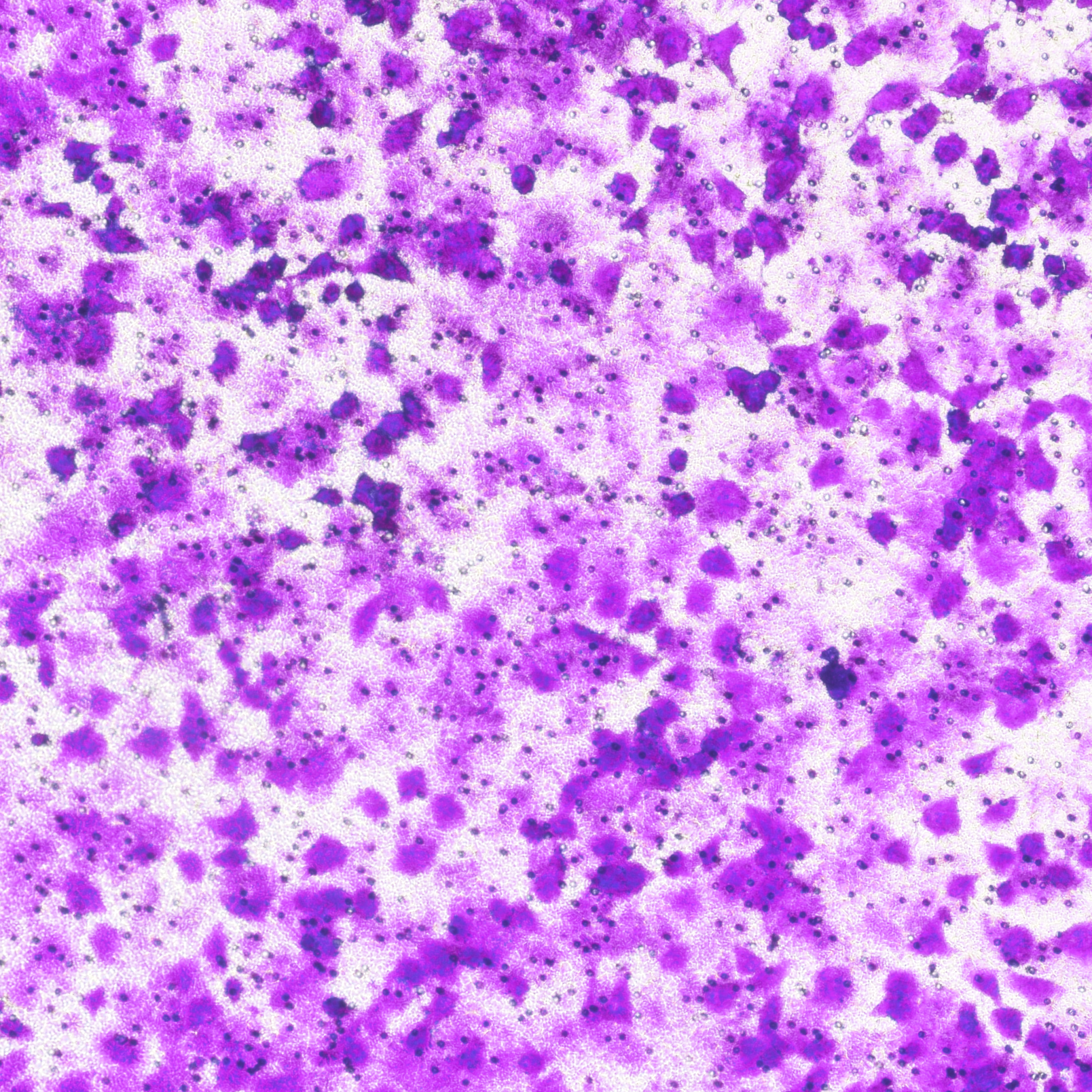


1975-250X


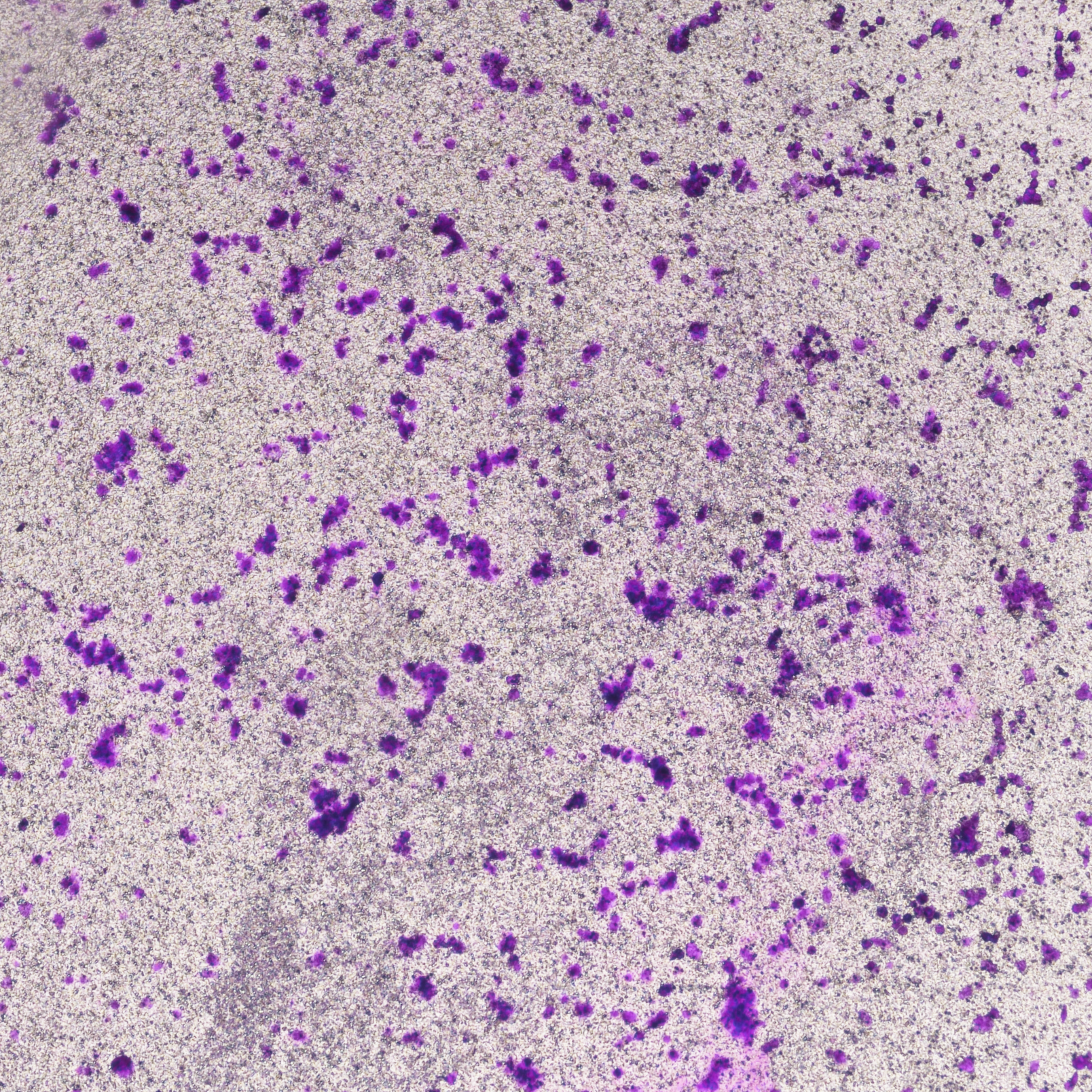


1975-MRPL13si-1-100X


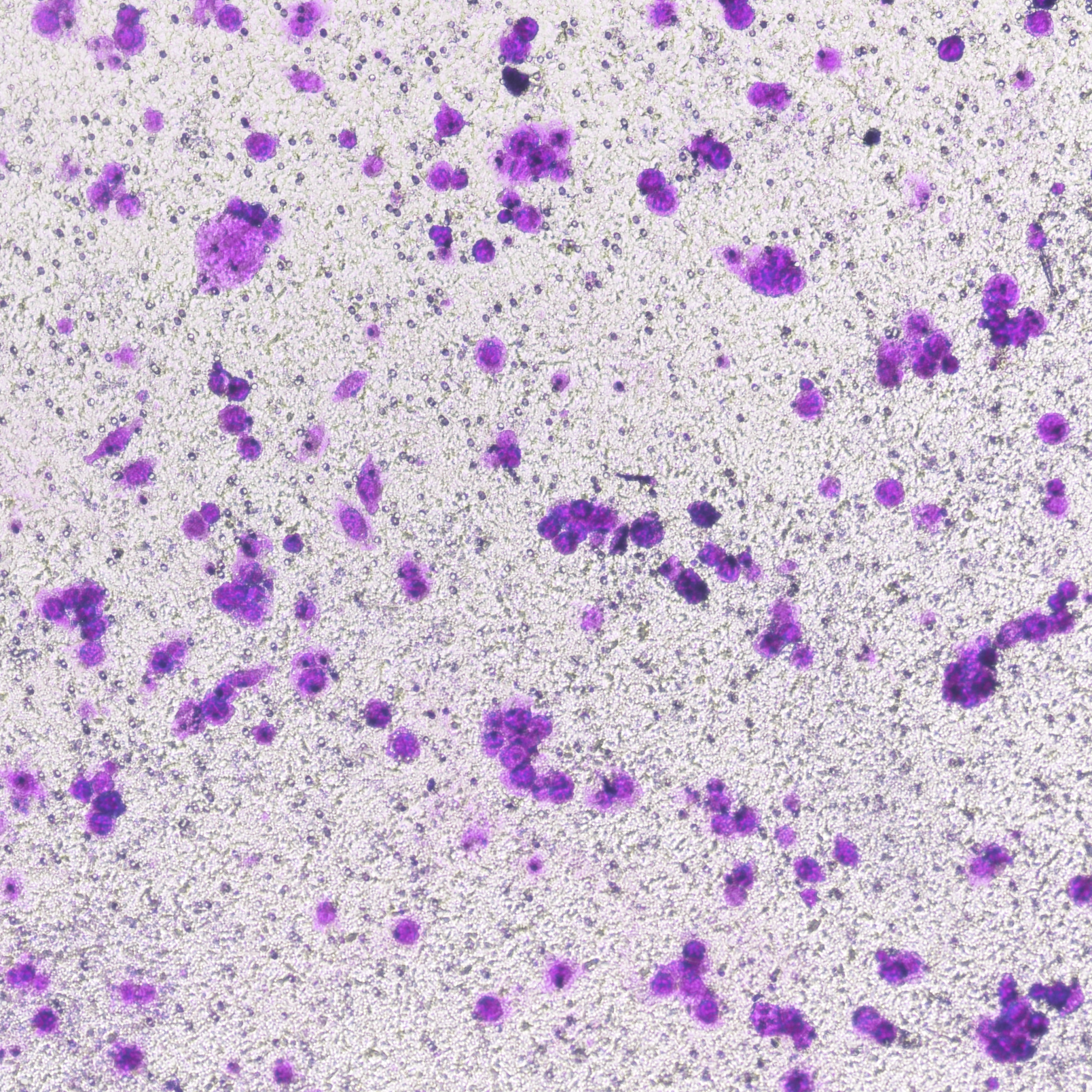


1975-MRPL13si-1-250X


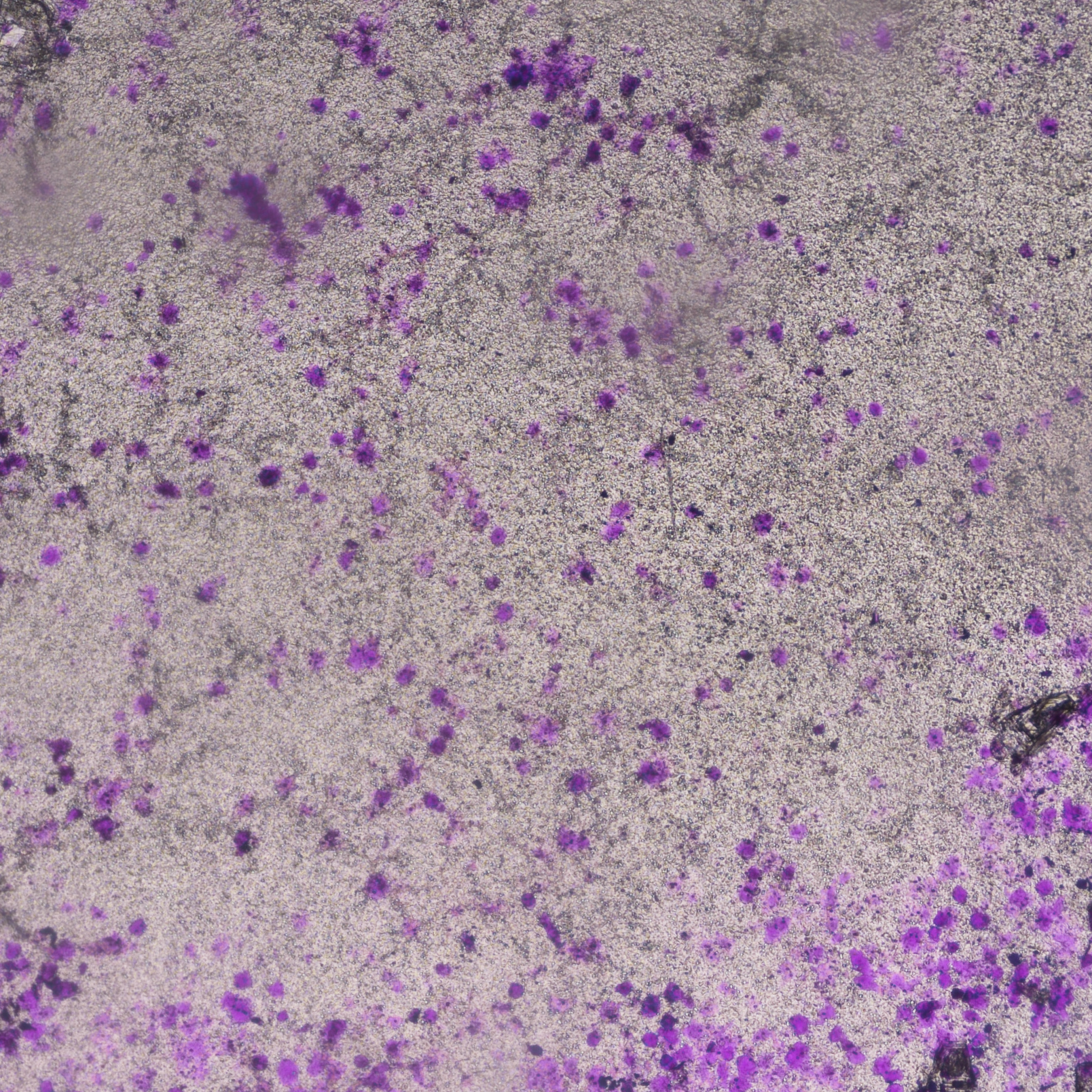


1975-MRPL13si-2-100X


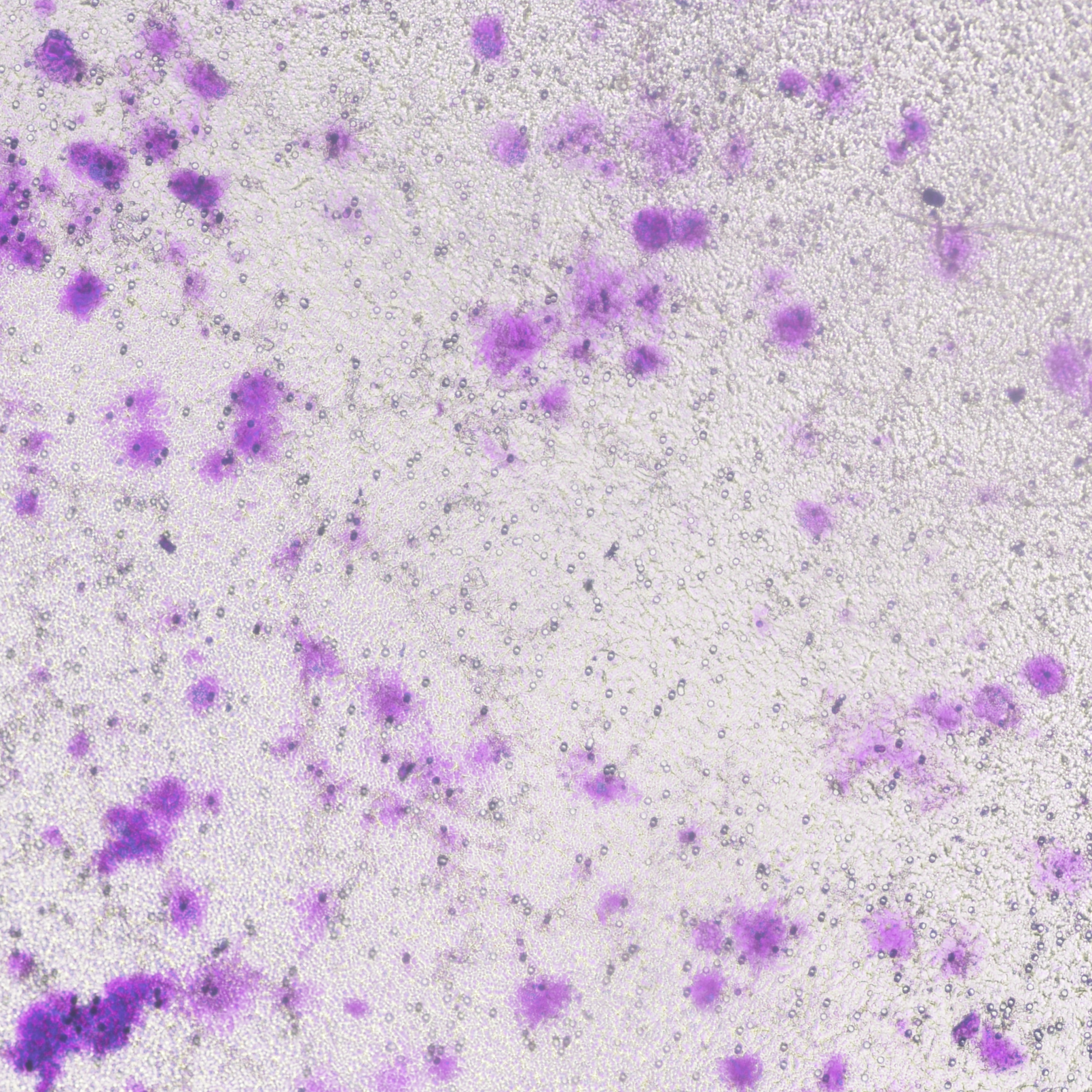


1975-MRPL13si-2-250X


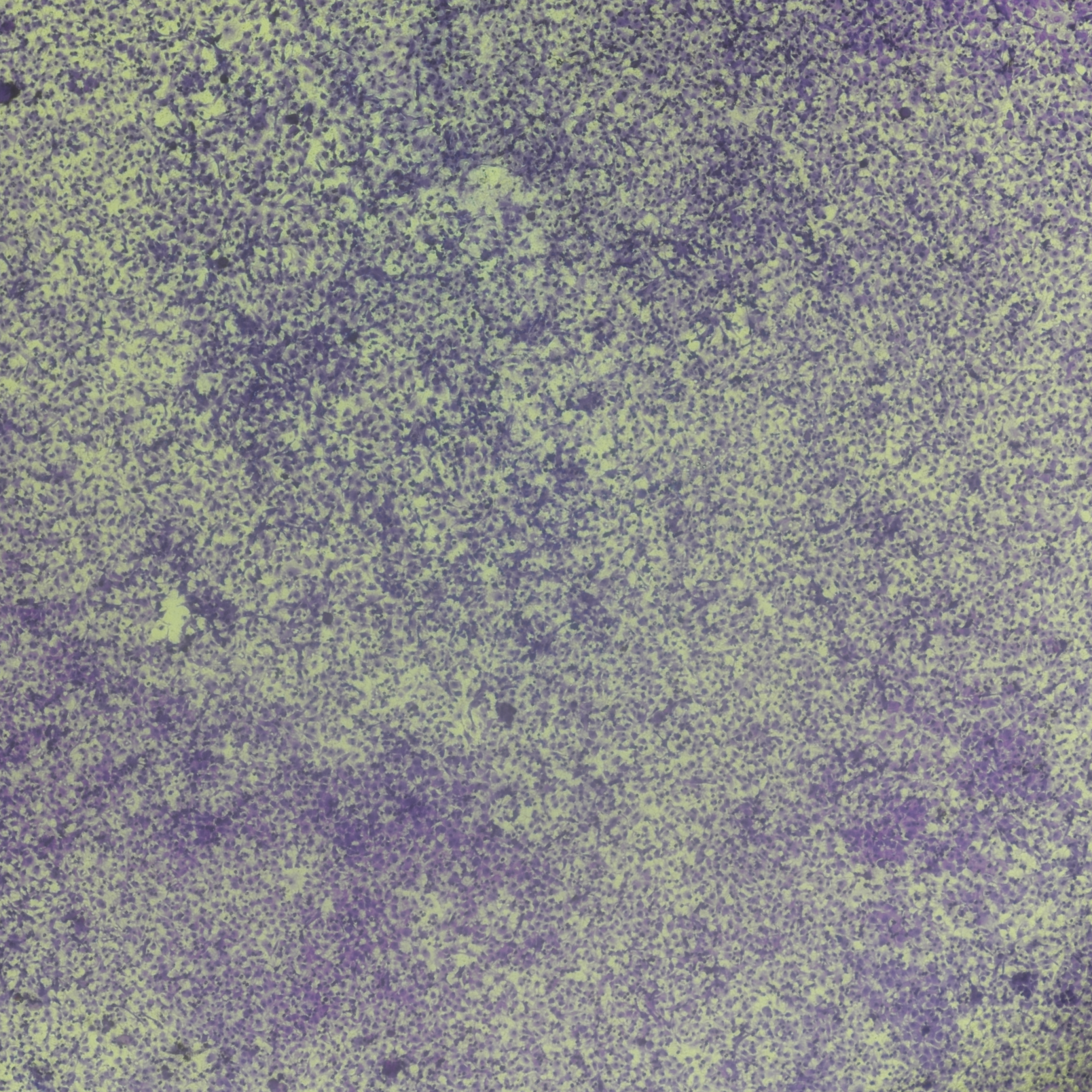


A549-100X


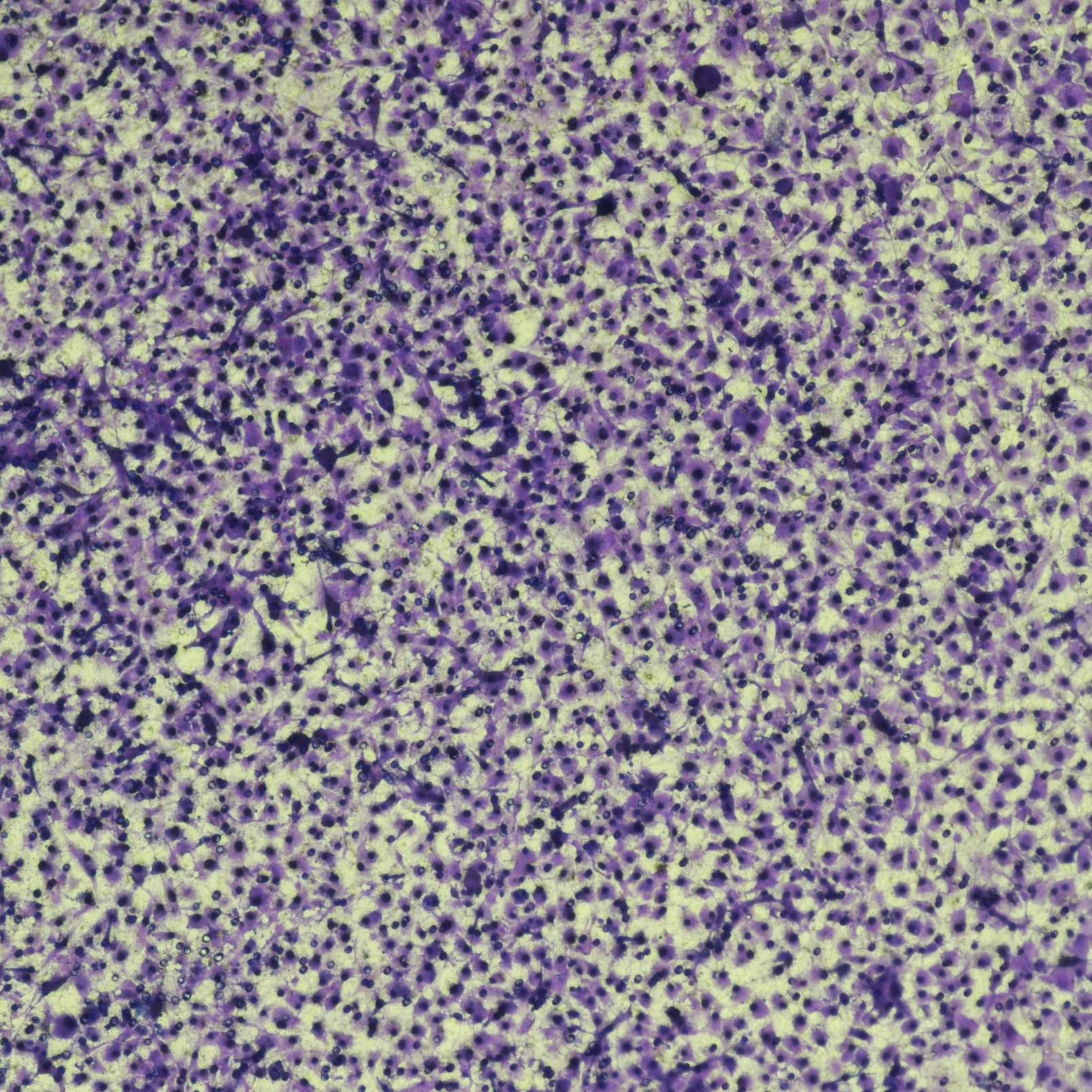


A549-250X


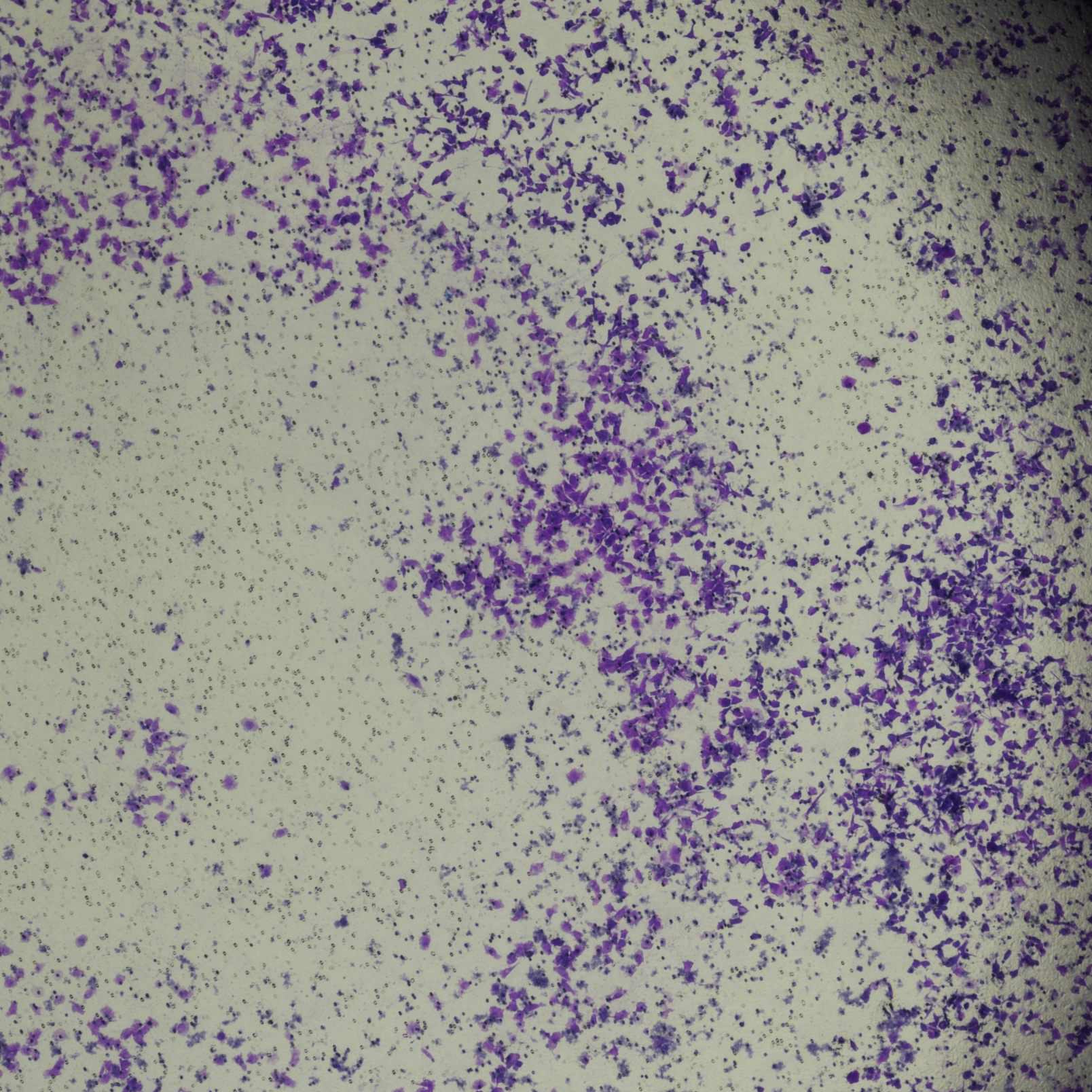


A549-MRPL13si-1-100X


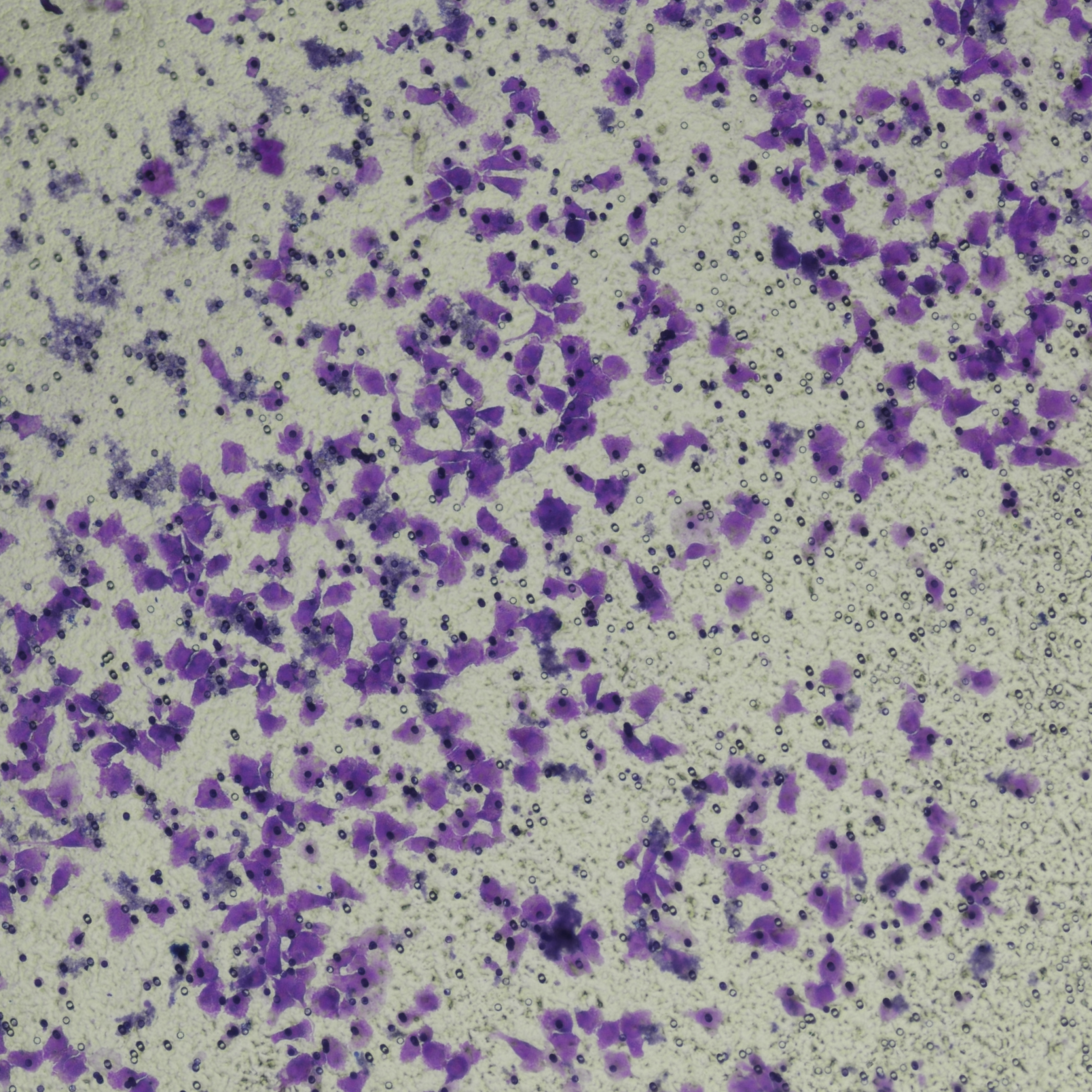


A549-MRPL13si-1-250X


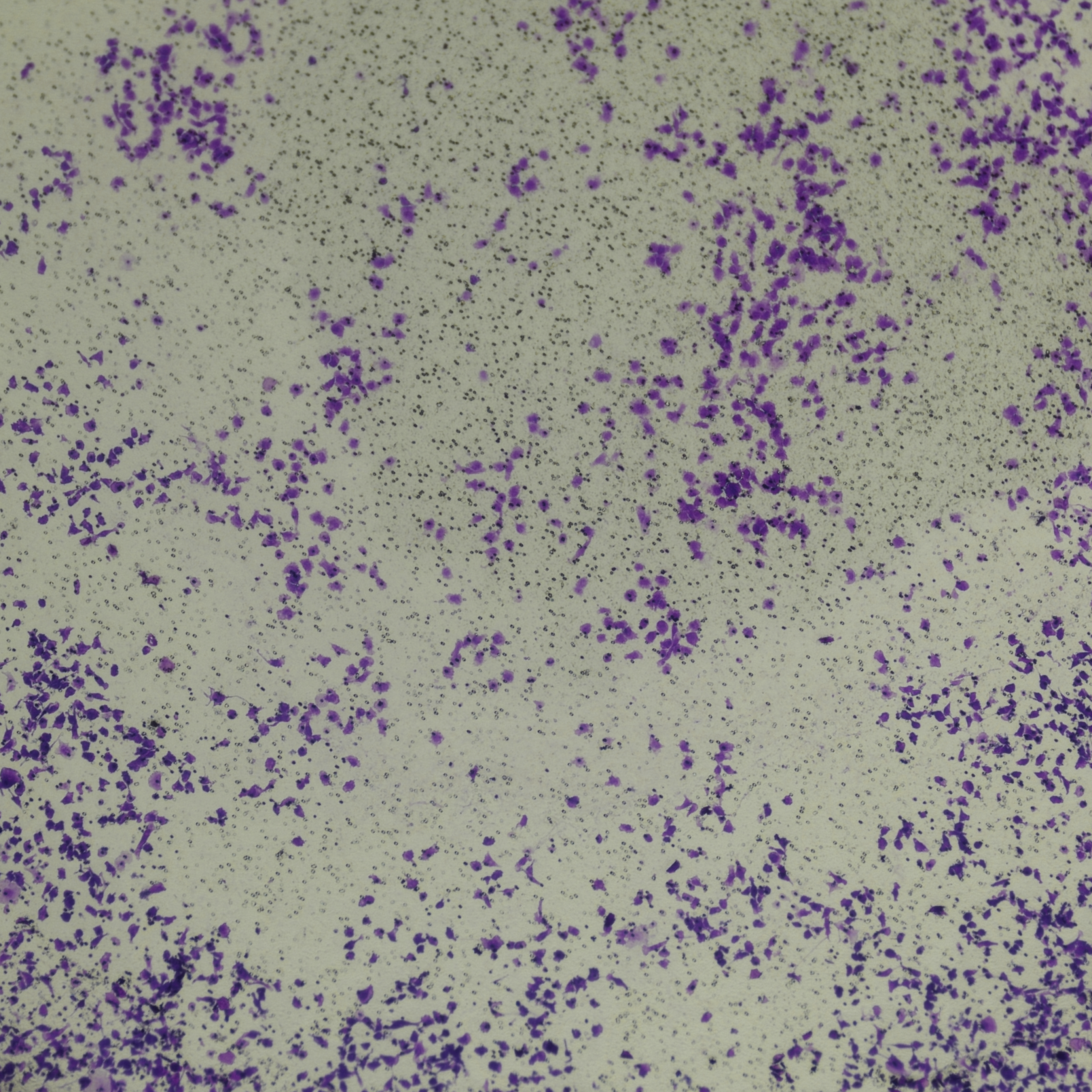


A549-MRPL13si-2-100X


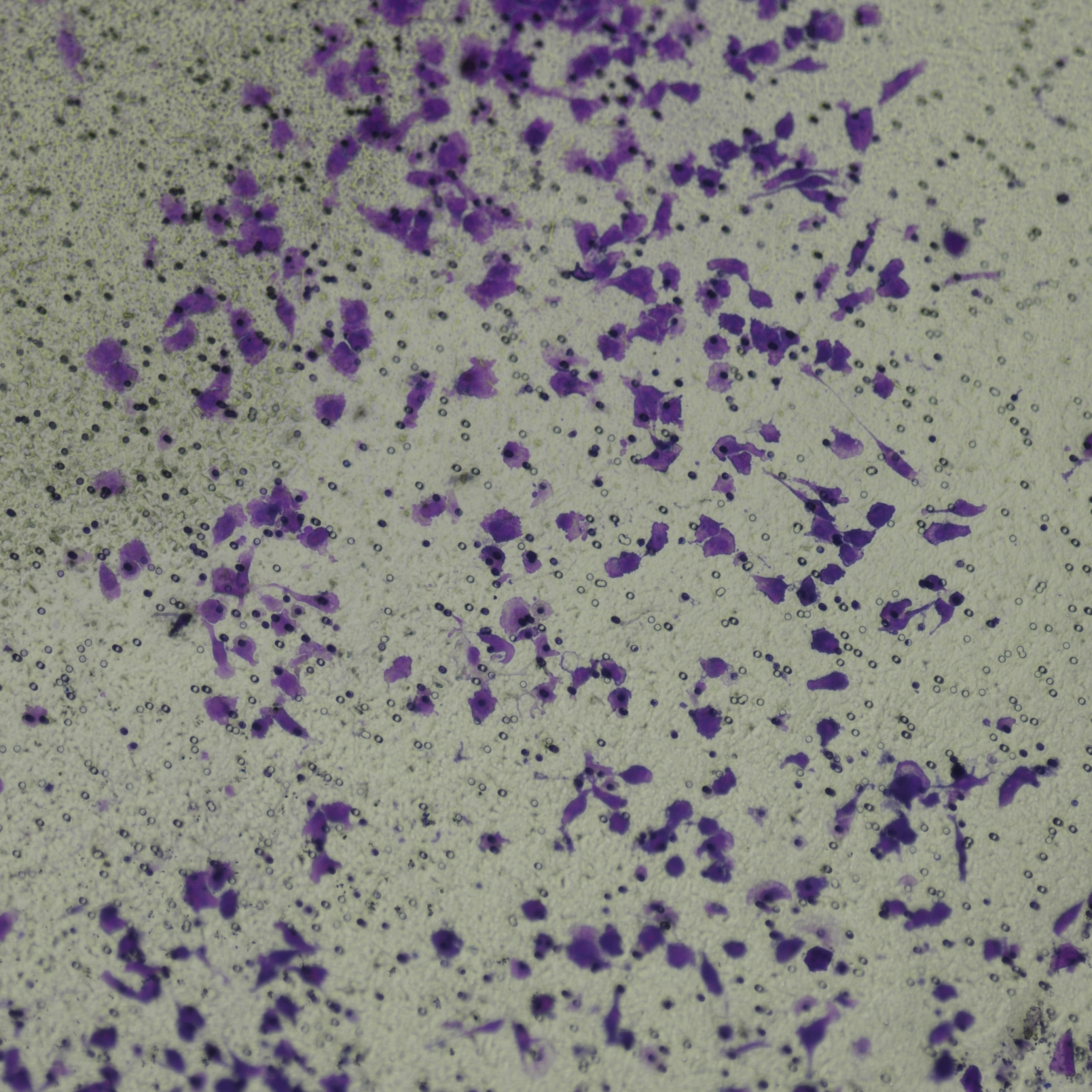


A549-MRPL13si-2-250X

| Gruop | frequency | MRPL13si-control | MRPL13si-1 | MRPL13si-2 |
| --- | --- | --- | --- | --- |
| A549-cell number | 1 | 8865 | 4820 | 5129 |
| NCI-H1975-cell-number | 1 | 4265 | 2514 | 2511 |
| A549-cell number | 2 | 8088 | 4605 | 4605 |
| NCI-H1975-cell-number | 2 | 4530 | 2700 | 2386 |
| A549-cell number | 3 | 8560 | 5020 | 5334 |
| NCI-H1975-cell-number | 3 | 4462 | 2350 | 2540 |
