# Supplementary material for: Multi-omics analysis of MRPL-13 as a tumor-promoting marker from pan-cancer to lung adenocarcinoma
Source: Aging (Albany NY). 2023 Oct 12;15(19):10640–80. doi: 10.18632/aging.205104 (PMC10599762; doi:10.18632/aging.205104)
Supplement: Supplementary Material 9 [file aging-15-205104-s008.docx]

**Supplementary Materials 9. Figure 14** **wound healing raw data 1.**


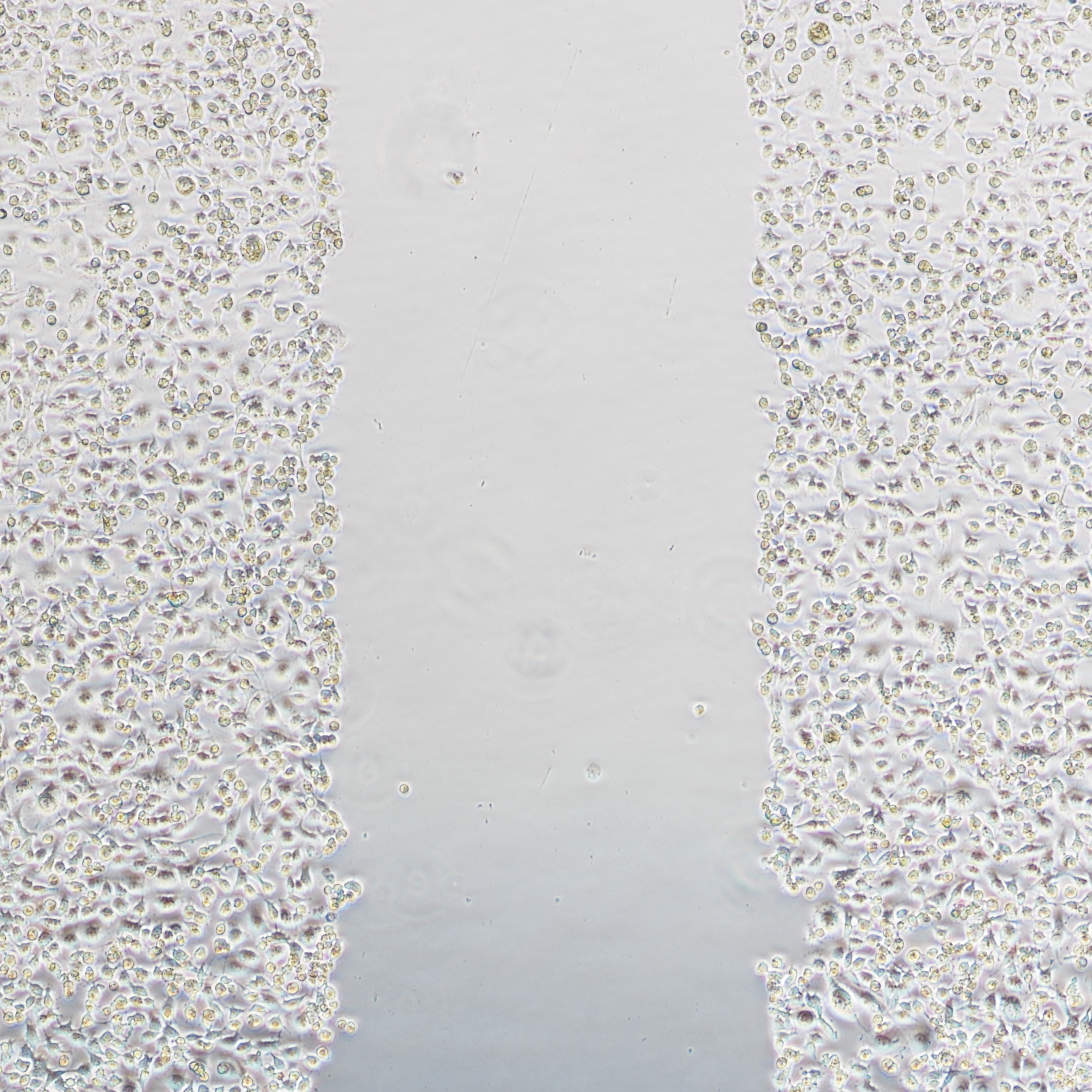


A549-0


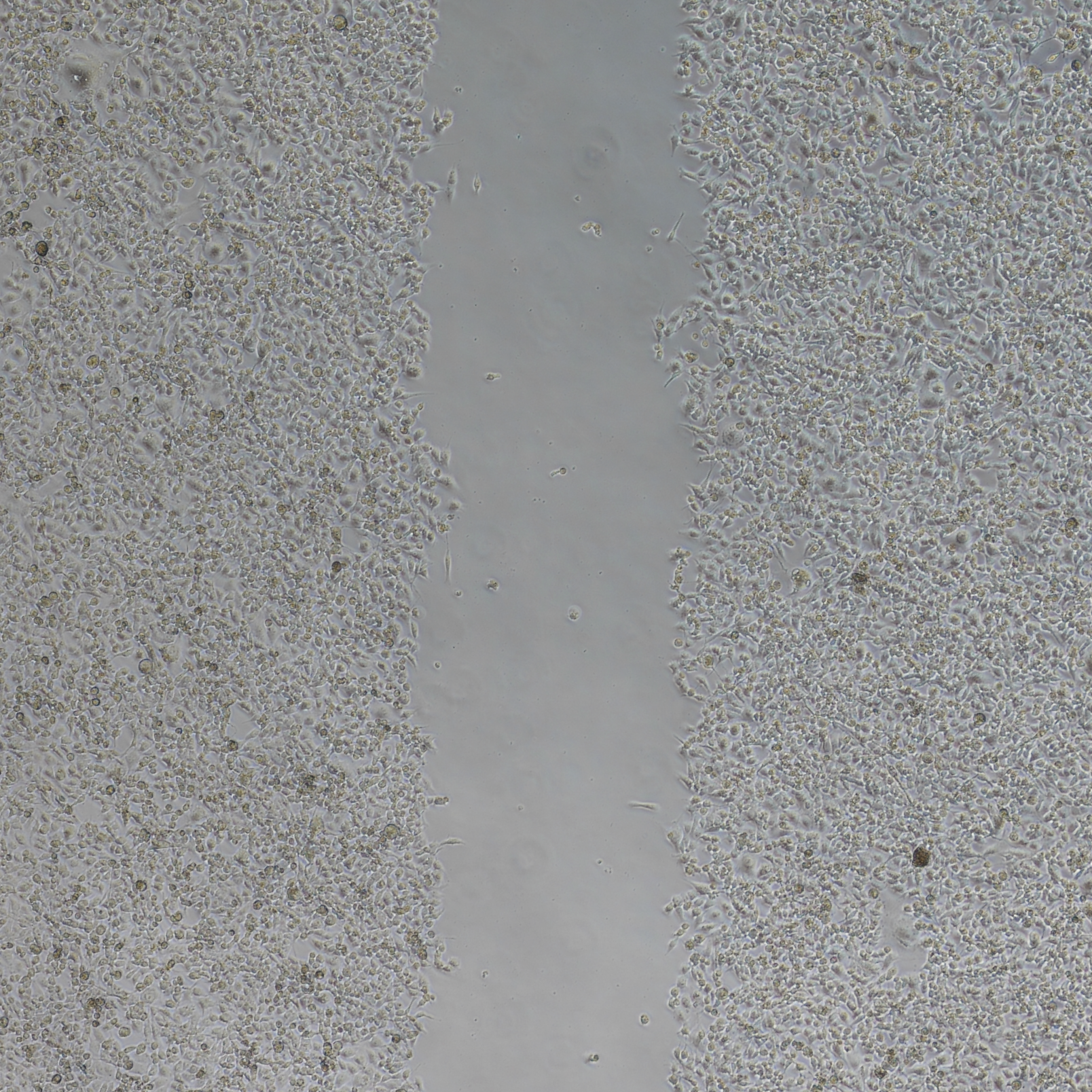


A549-24


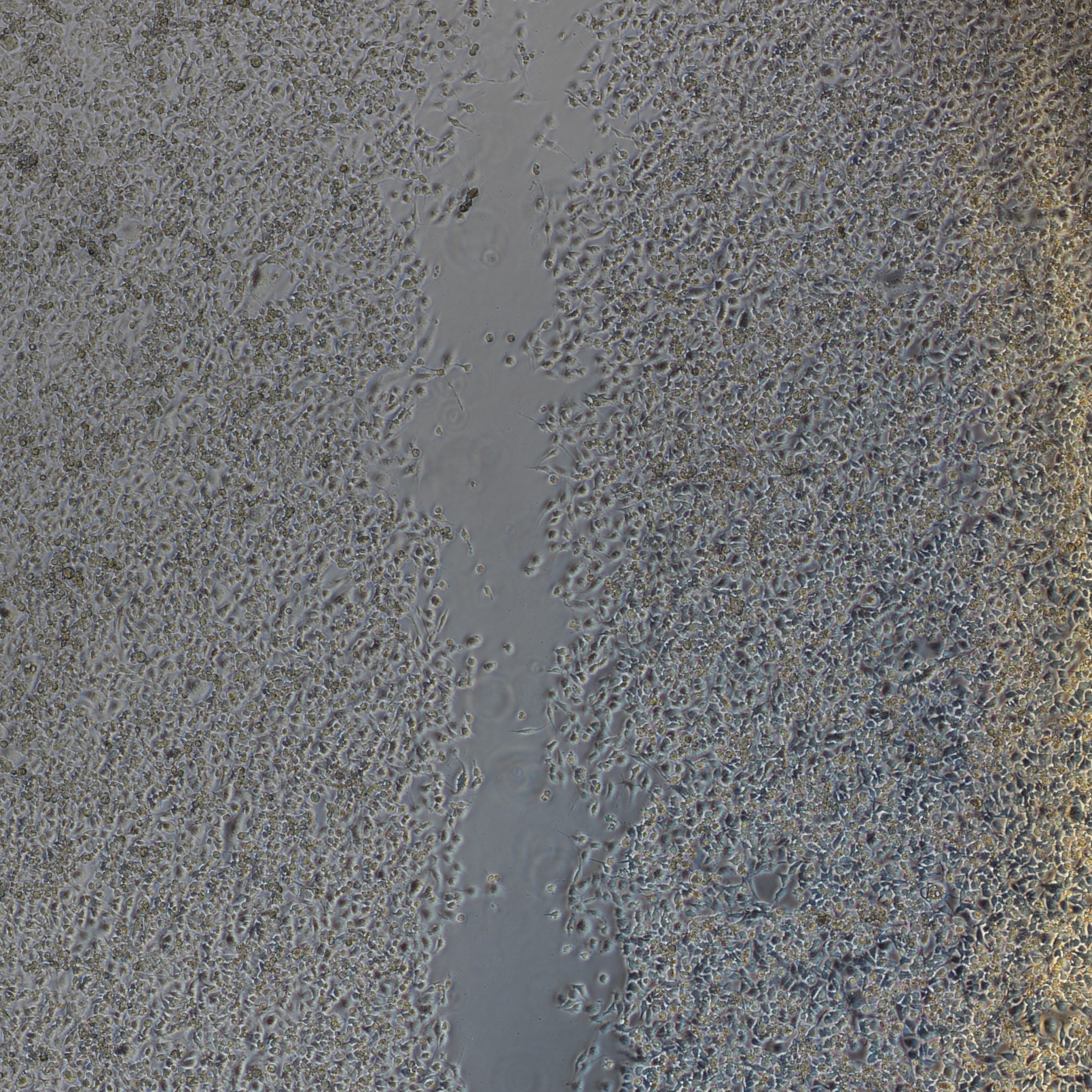


A549-48


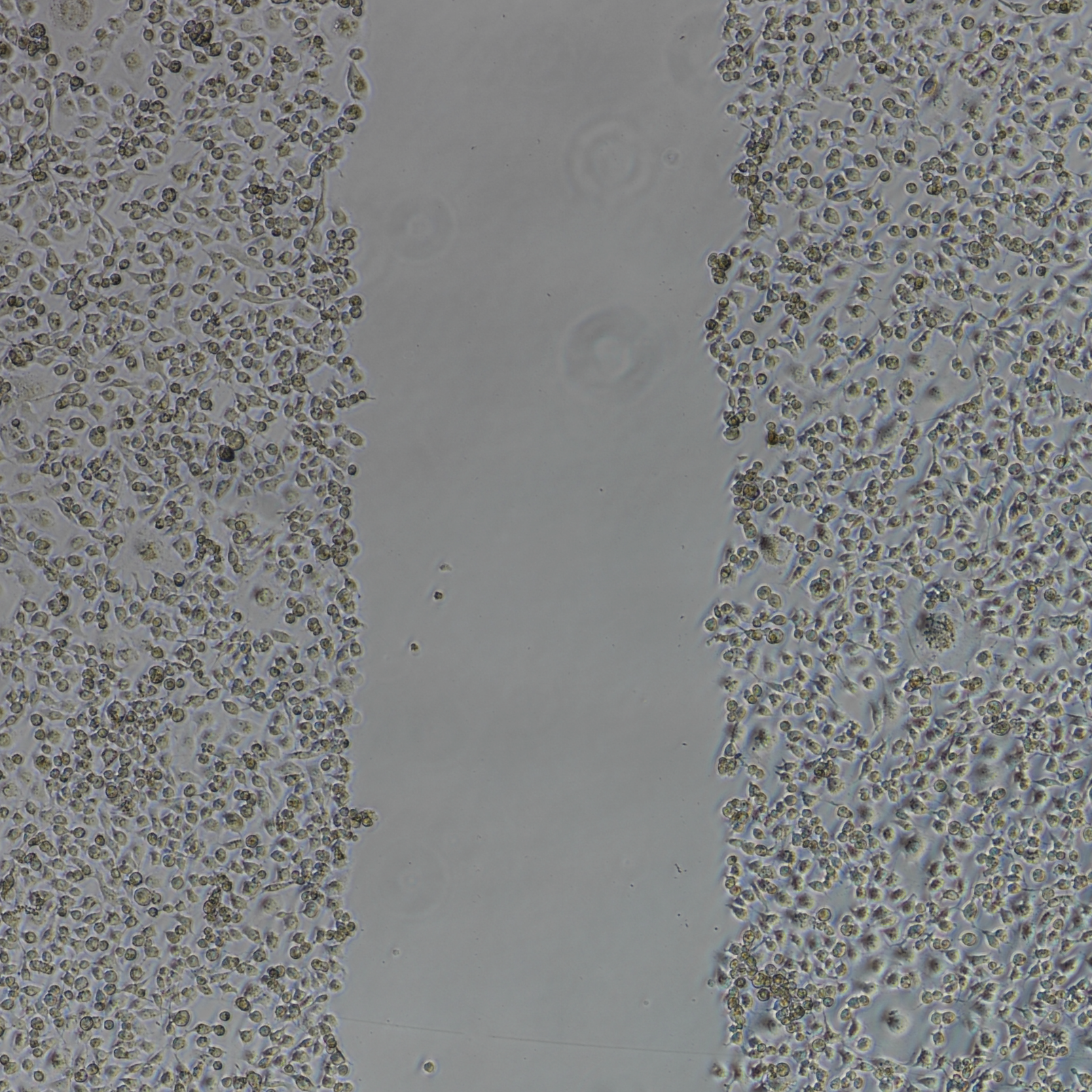


A549-MRPL13si-1-0


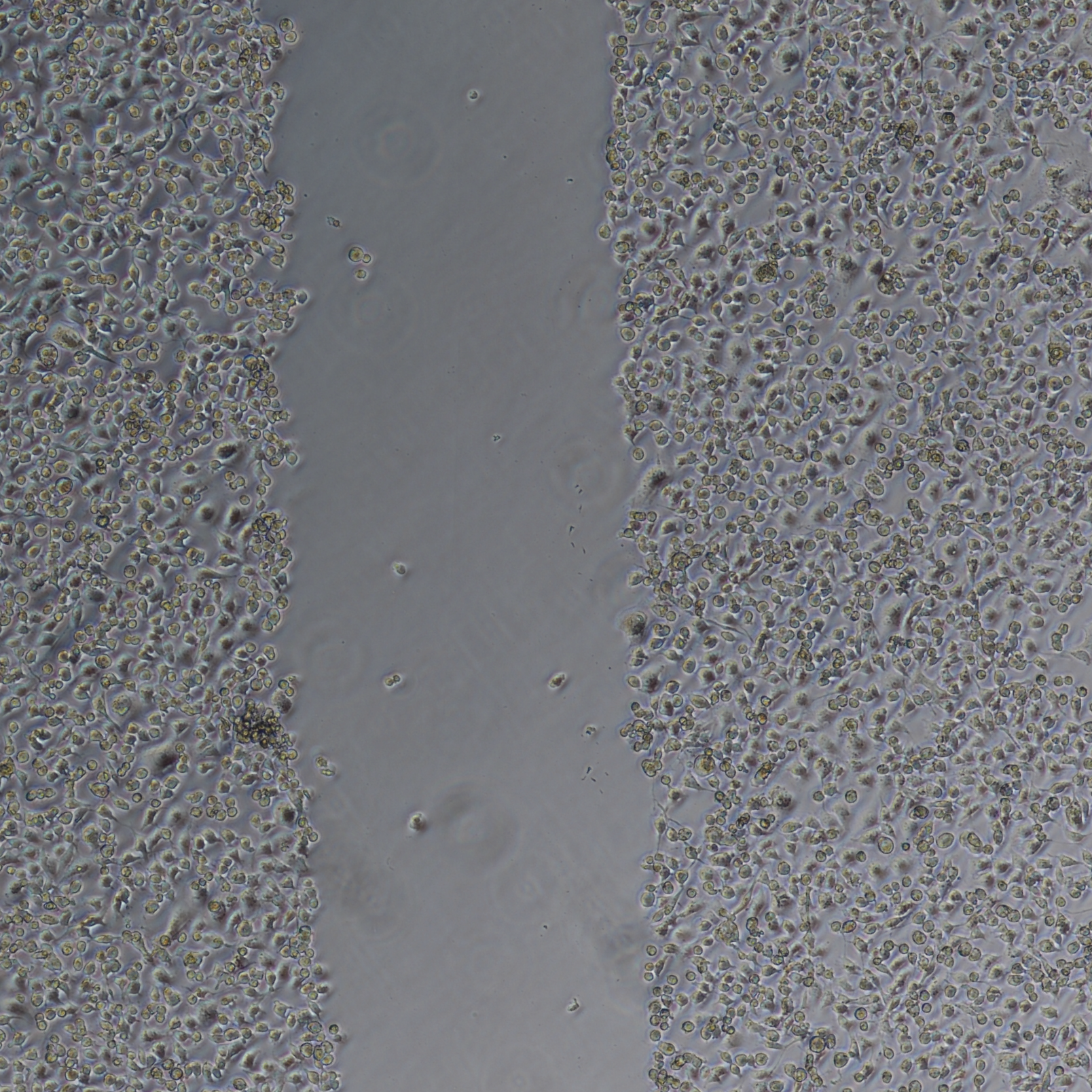


A549-MRPL13si-1-24


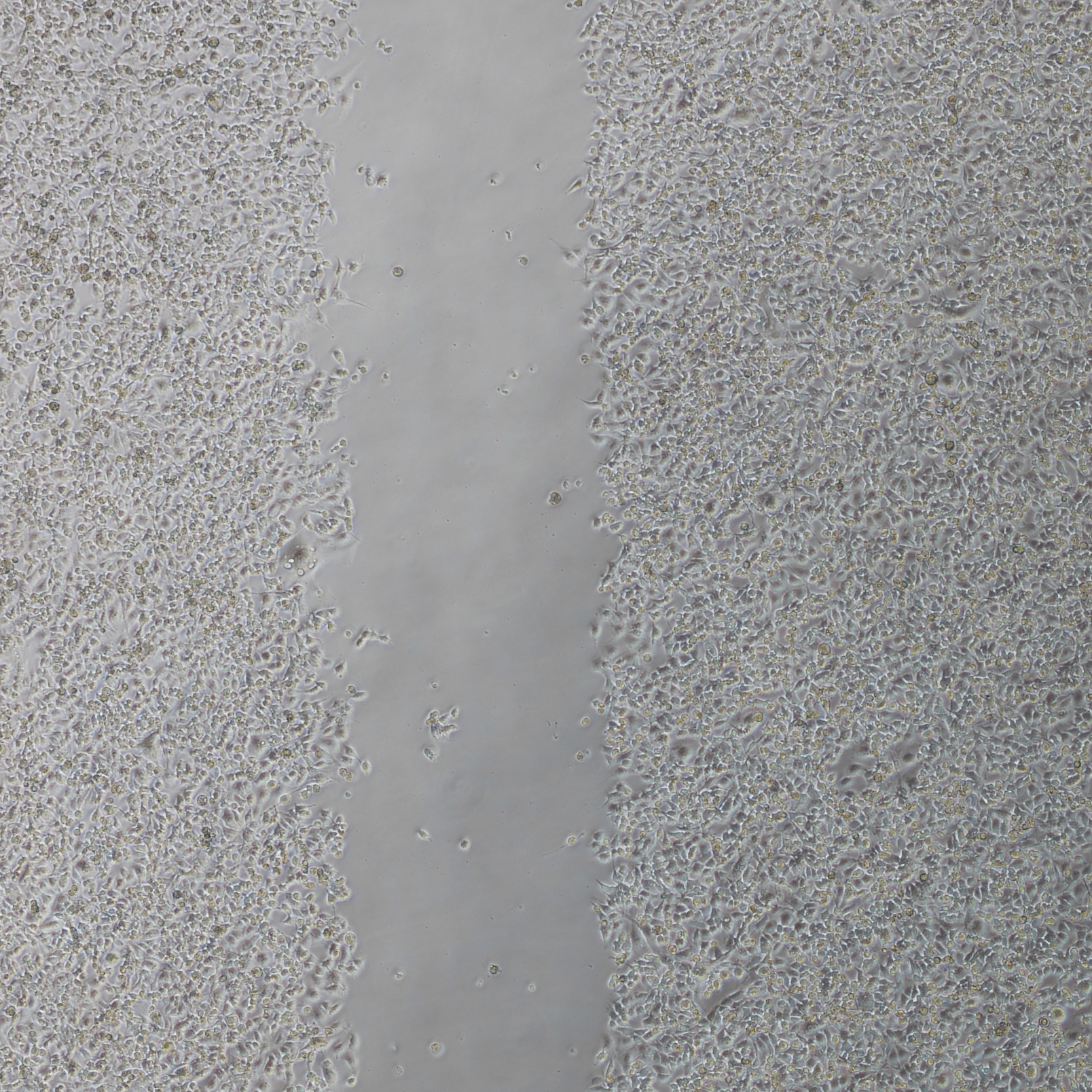


A549-MRPL13si-1-48


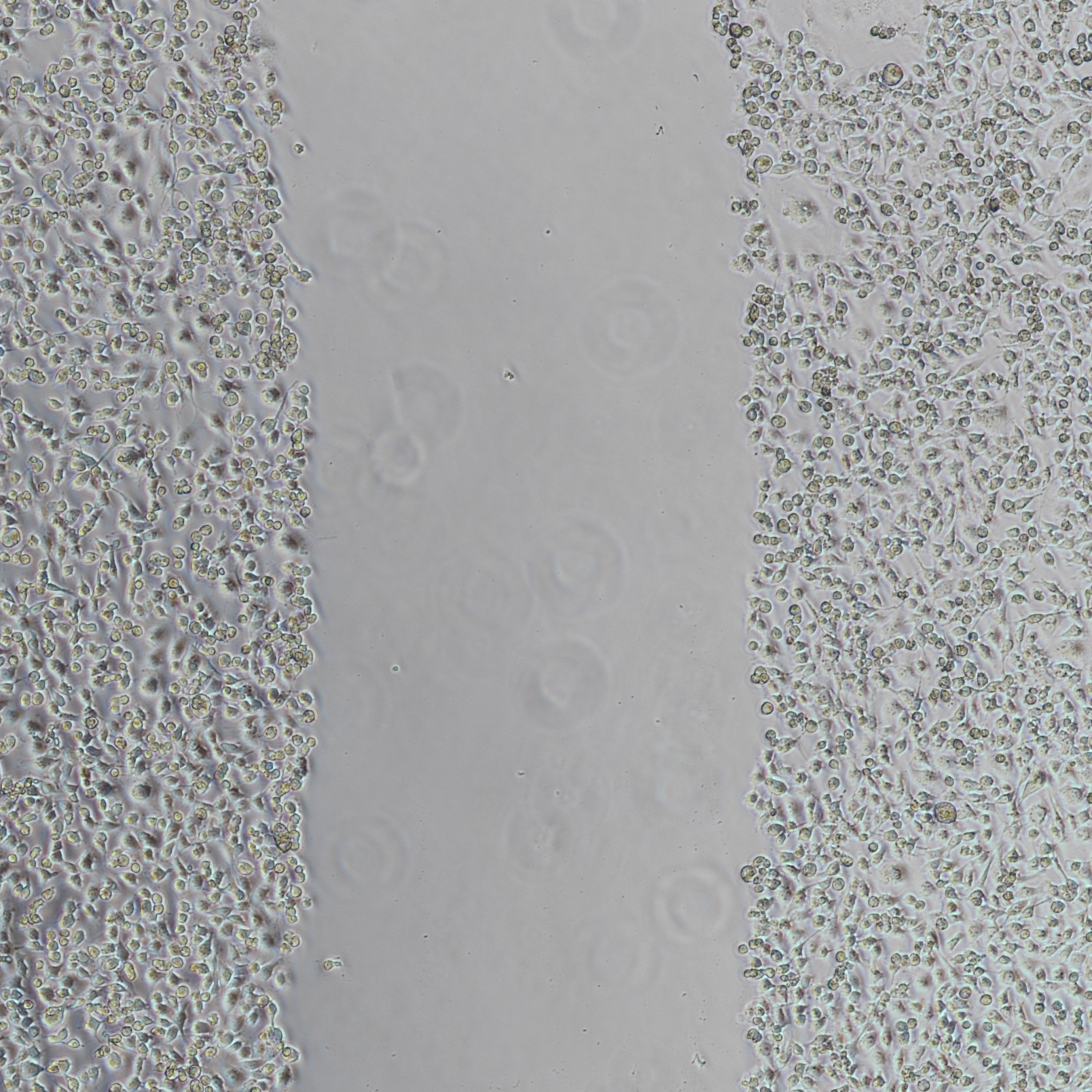


A549-MRPL13si-2-0


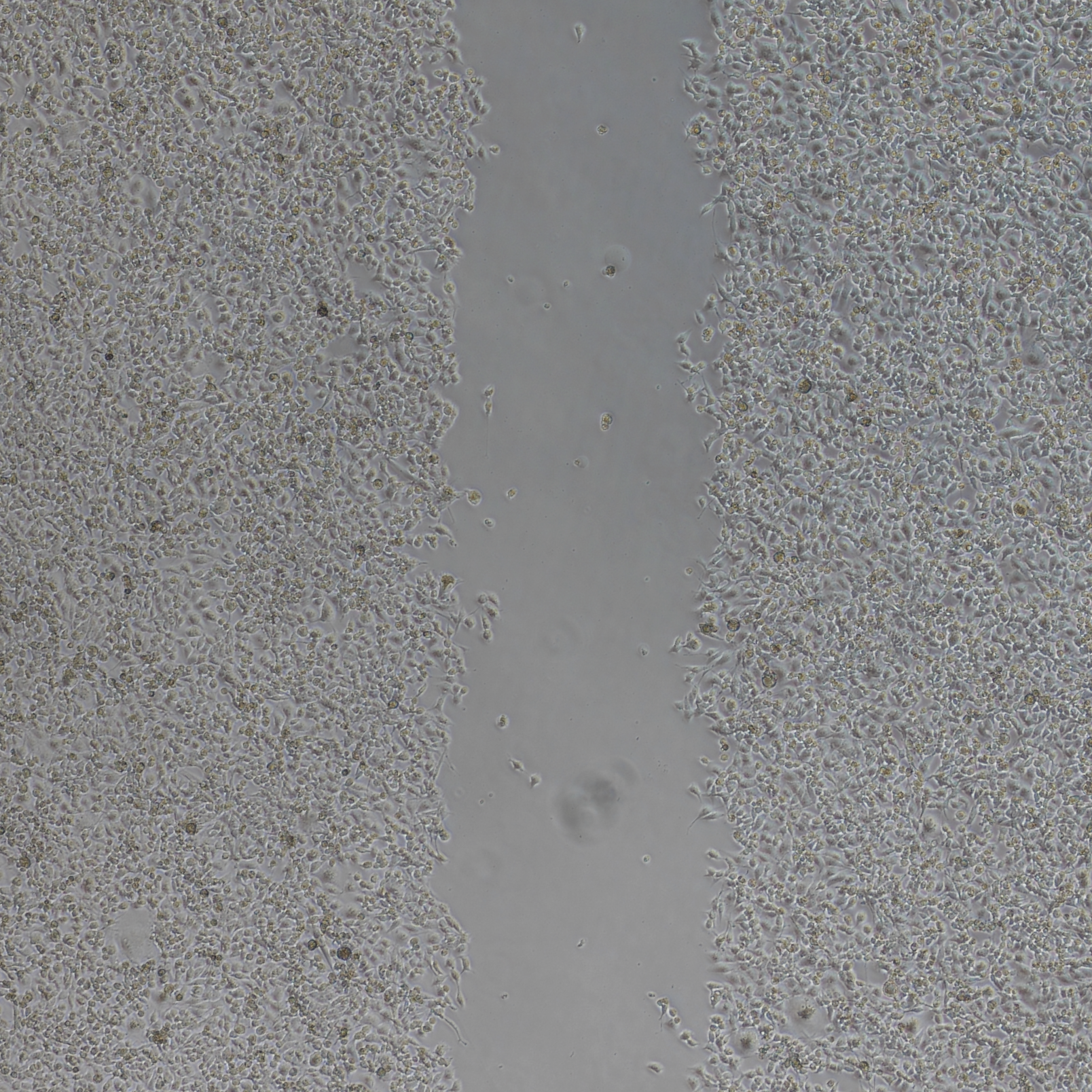


A549-MRPL13si-2-24


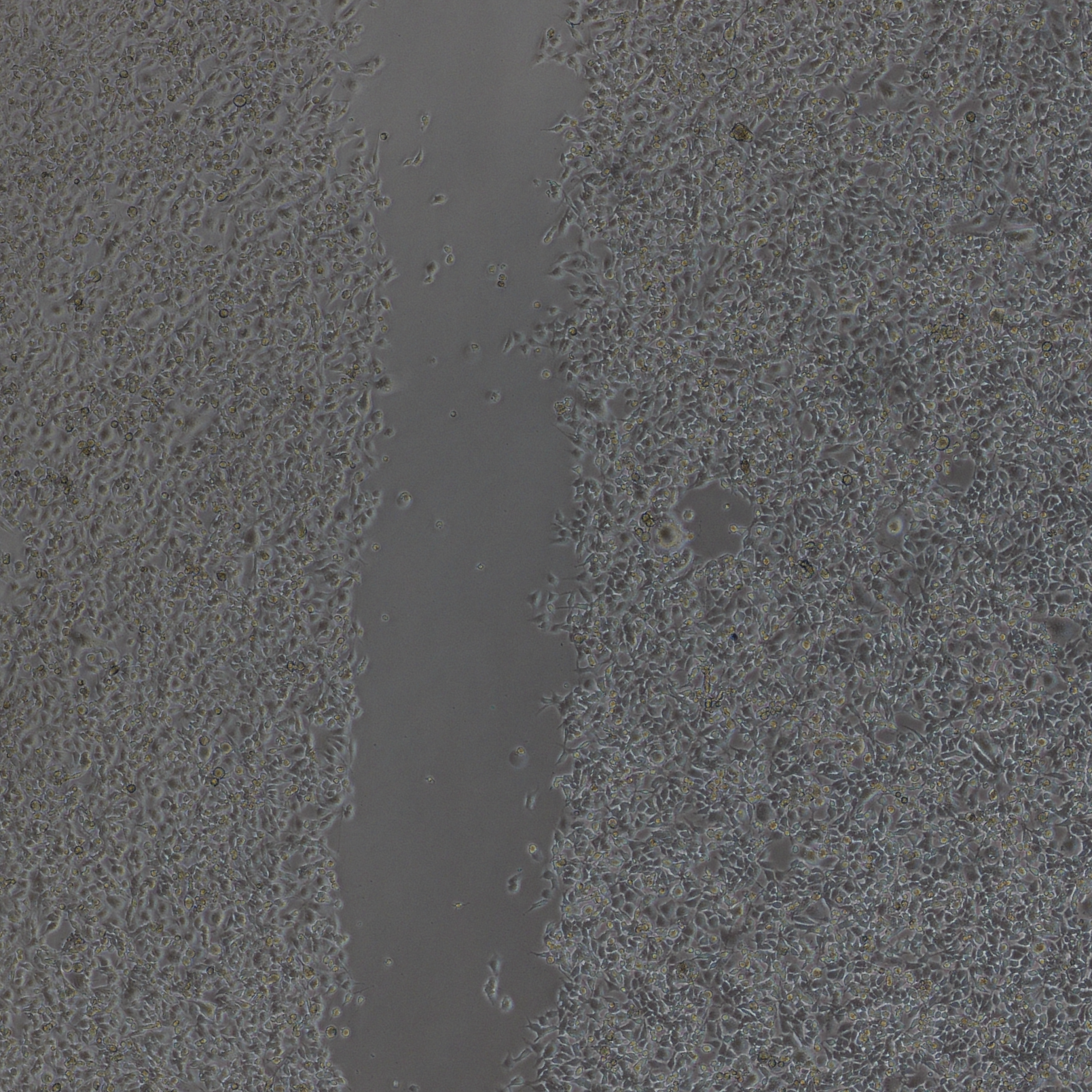


A549-MRPL13si-2-48
