# Supplementary material for: Multi-omics analysis of MRPL-13 as a tumor-promoting marker from pan-cancer to lung adenocarcinoma
Source: Aging (Albany NY). 2023 Oct 12;15(19):10640–80. doi: 10.18632/aging.205104 (PMC10599762; doi:10.18632/aging.205104)
Supplement: Supplementary Material 10 [file aging-15-205104-s009.docx]

**Supplementary Materials 10. Figure 14 wound healing raw data 2.**


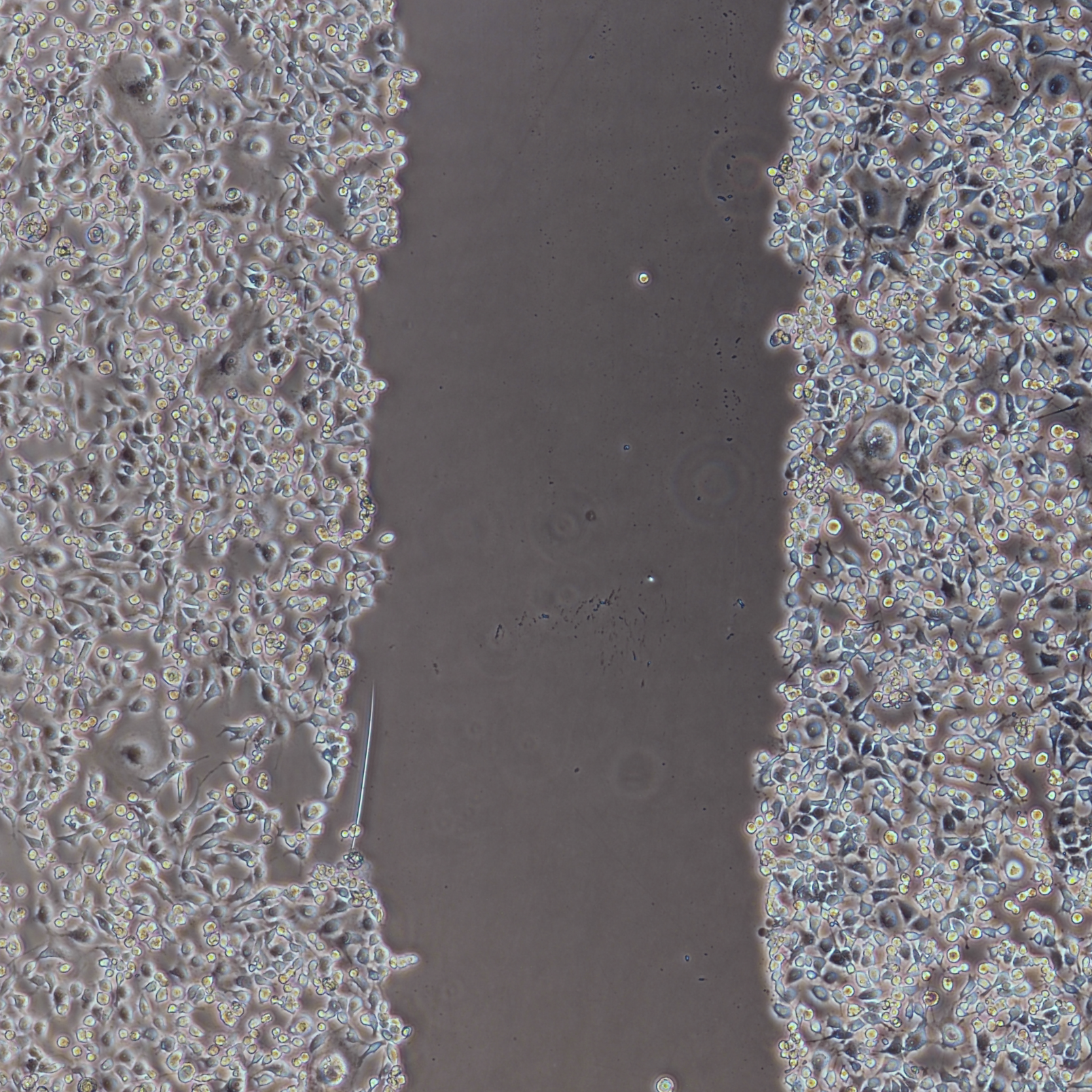


1975-0


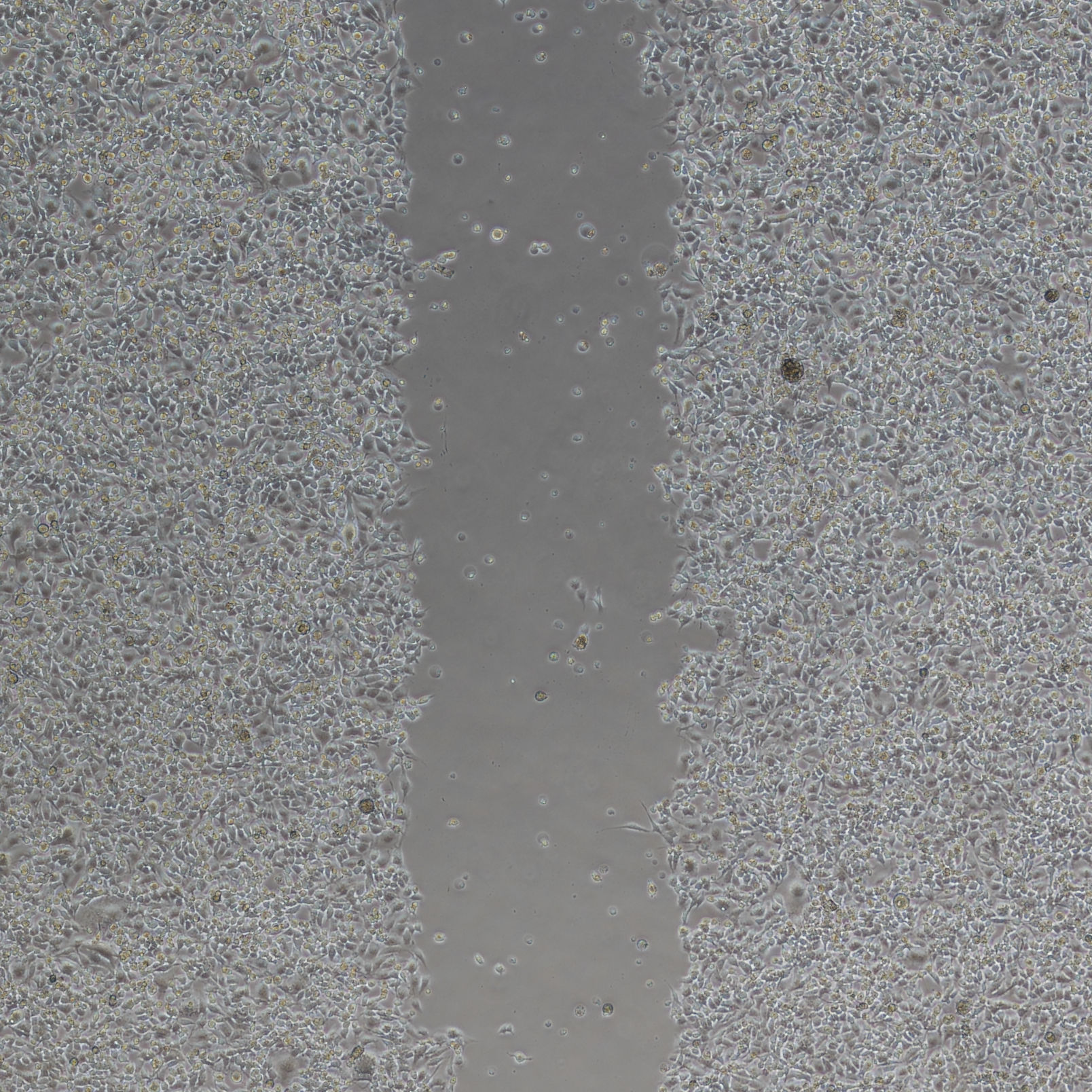


1975-24


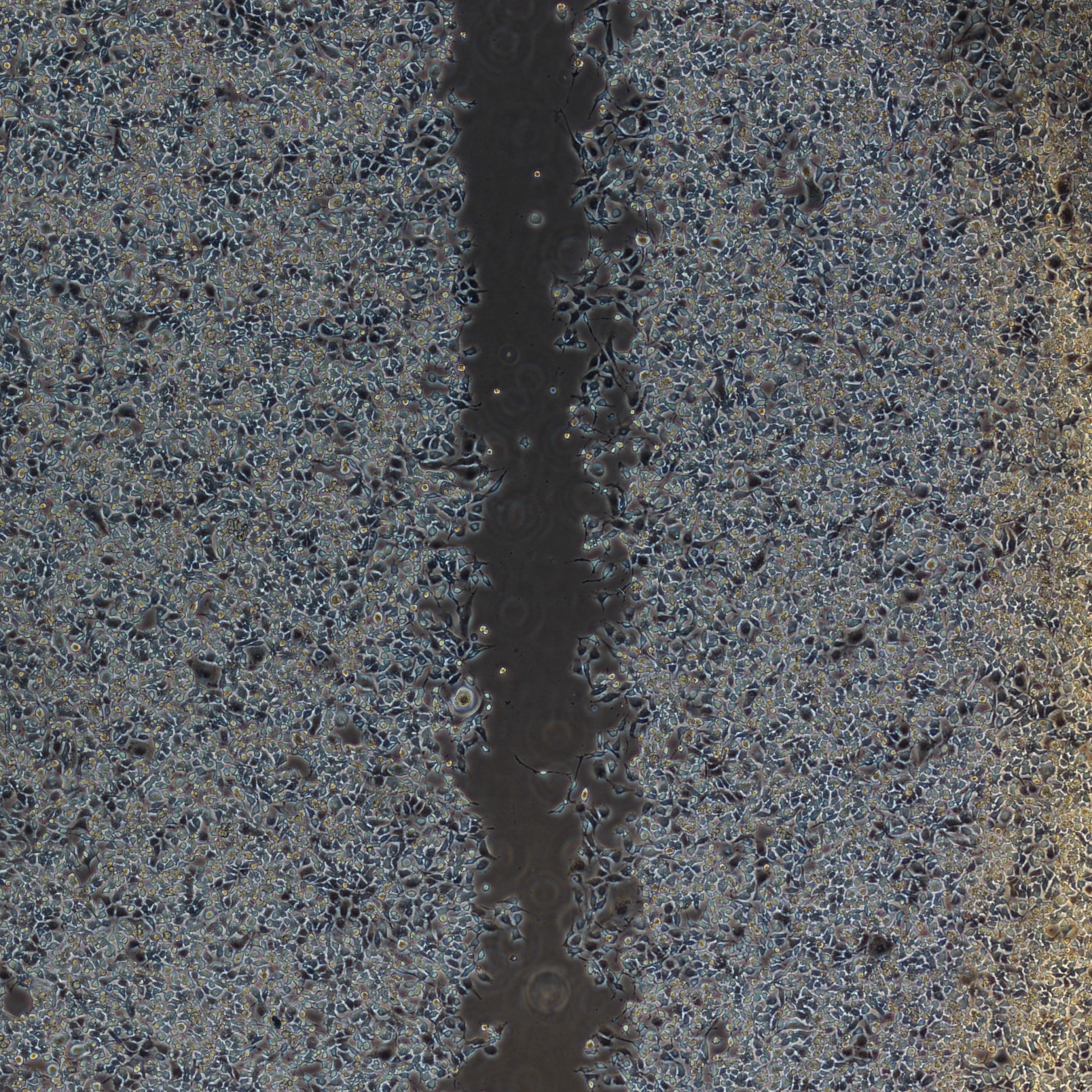


1975-48


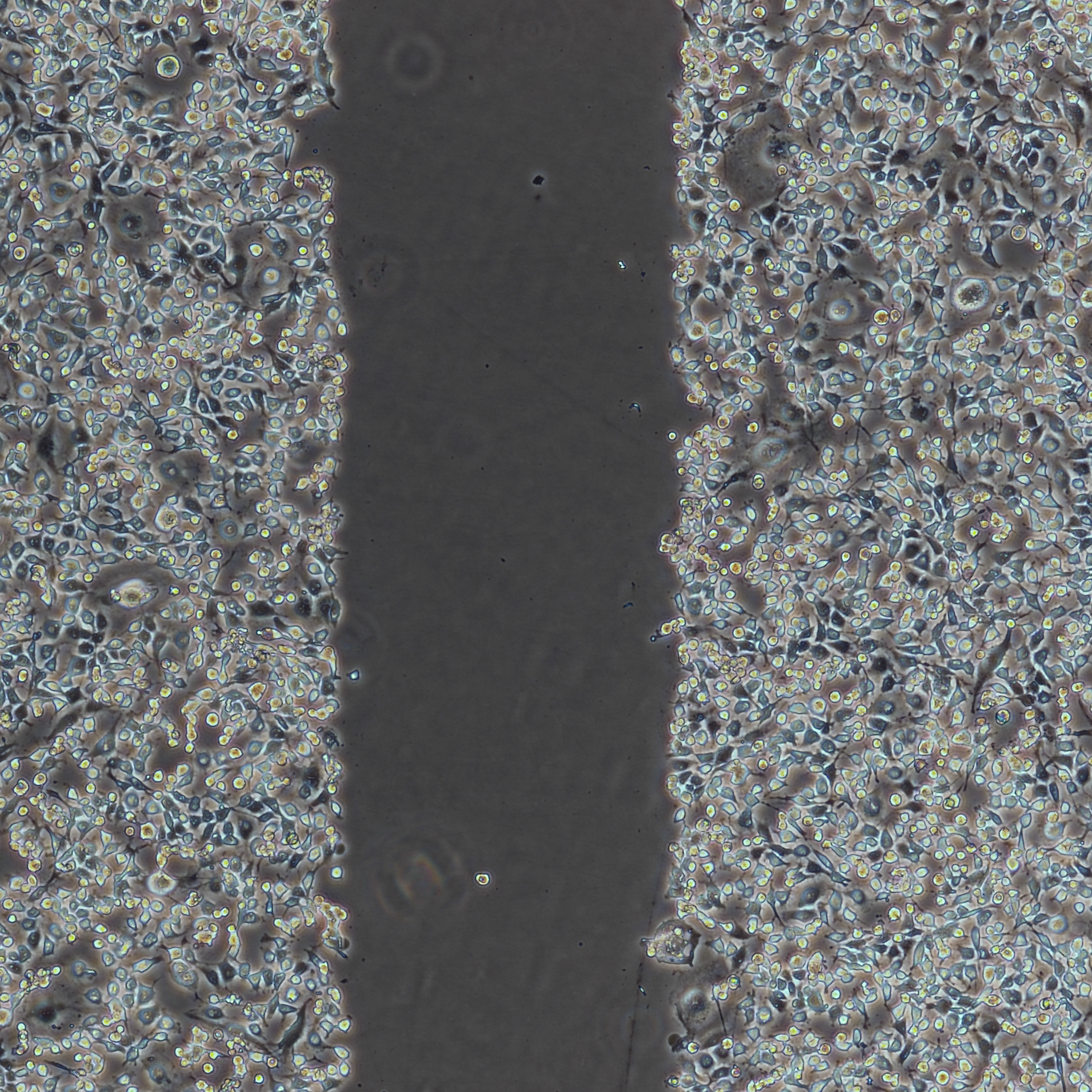


1975-MRPL13si-1-0


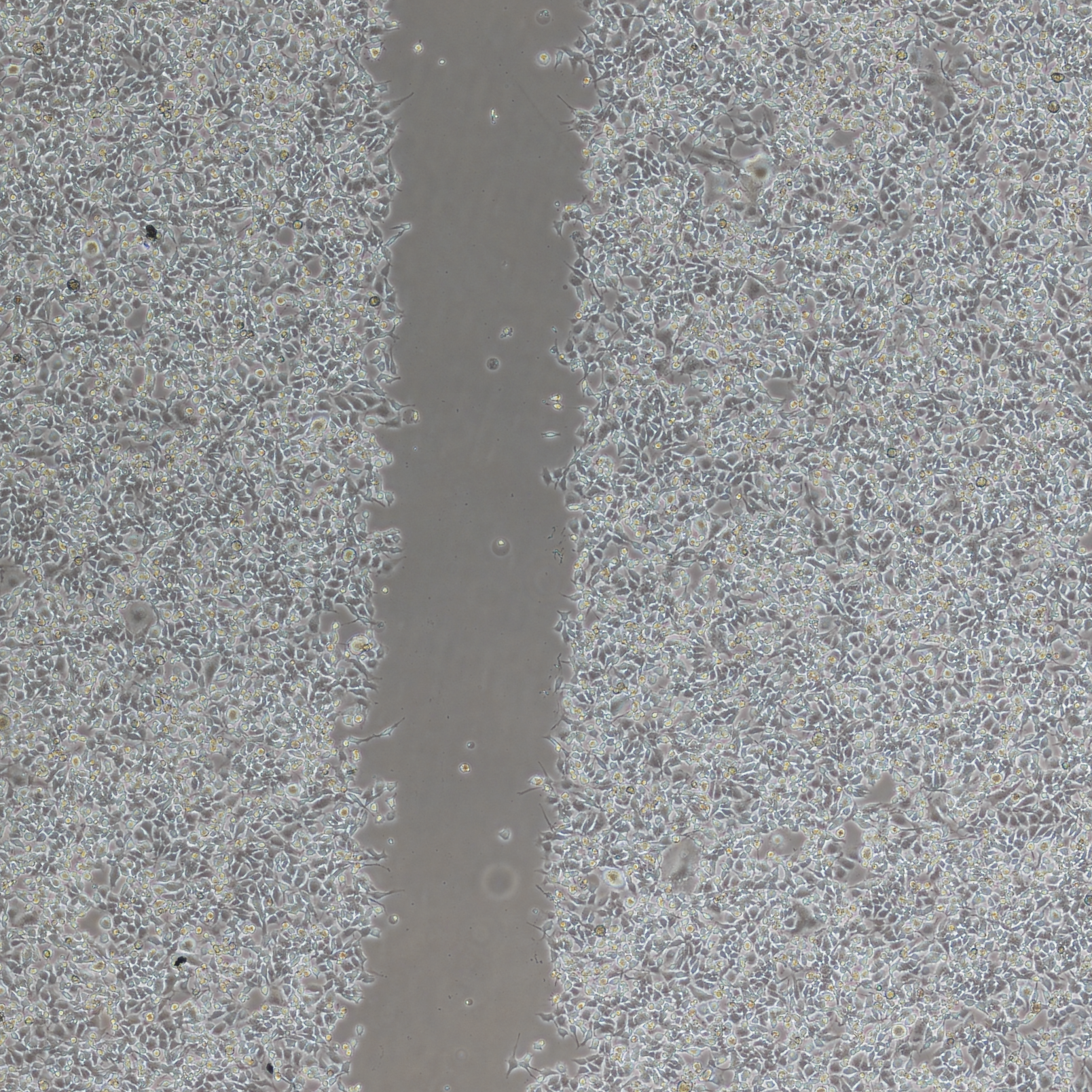


1975-MRPL13si-1-0-24


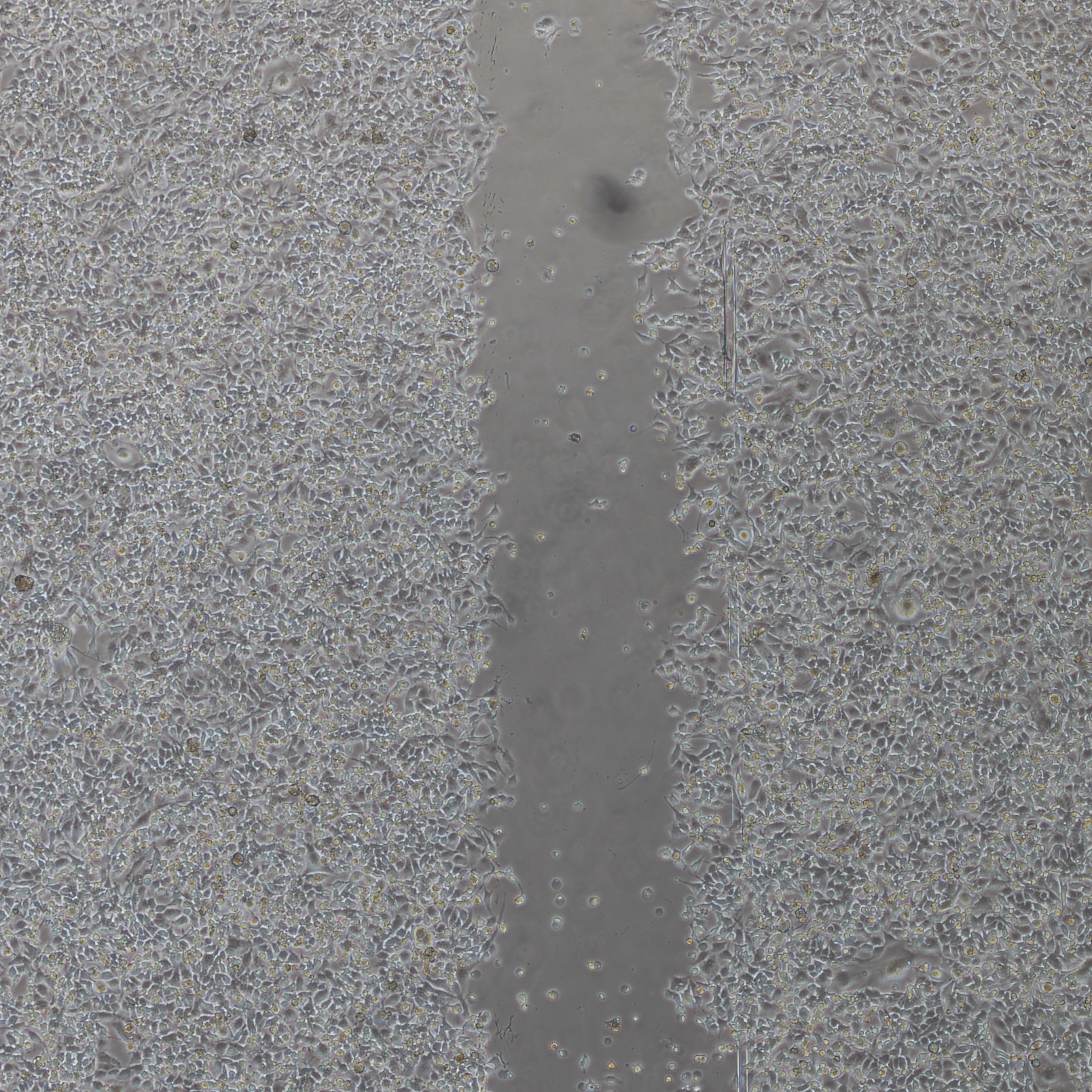


1975-MRPL13si-1-0-48


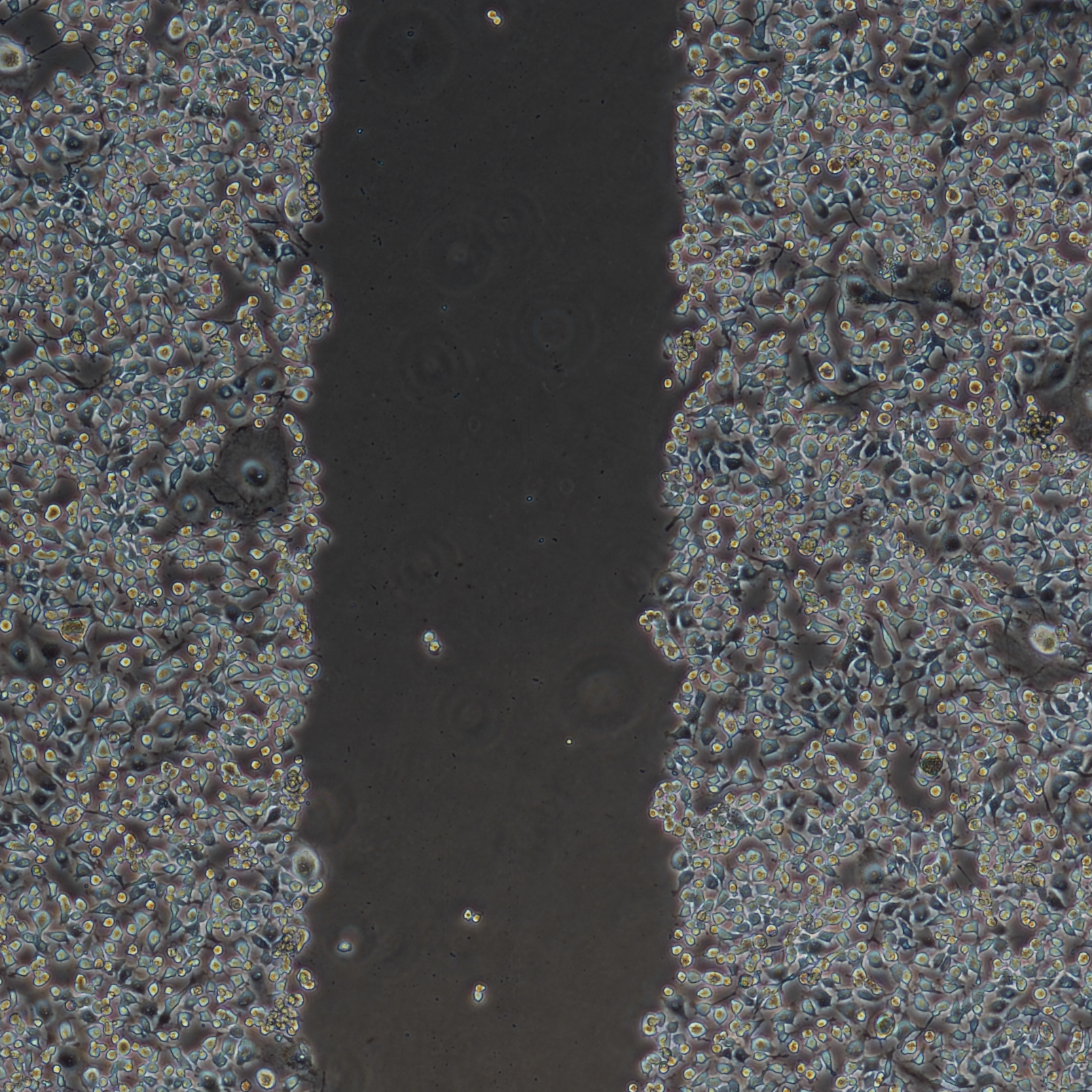


1975-MRPL13si-2-0


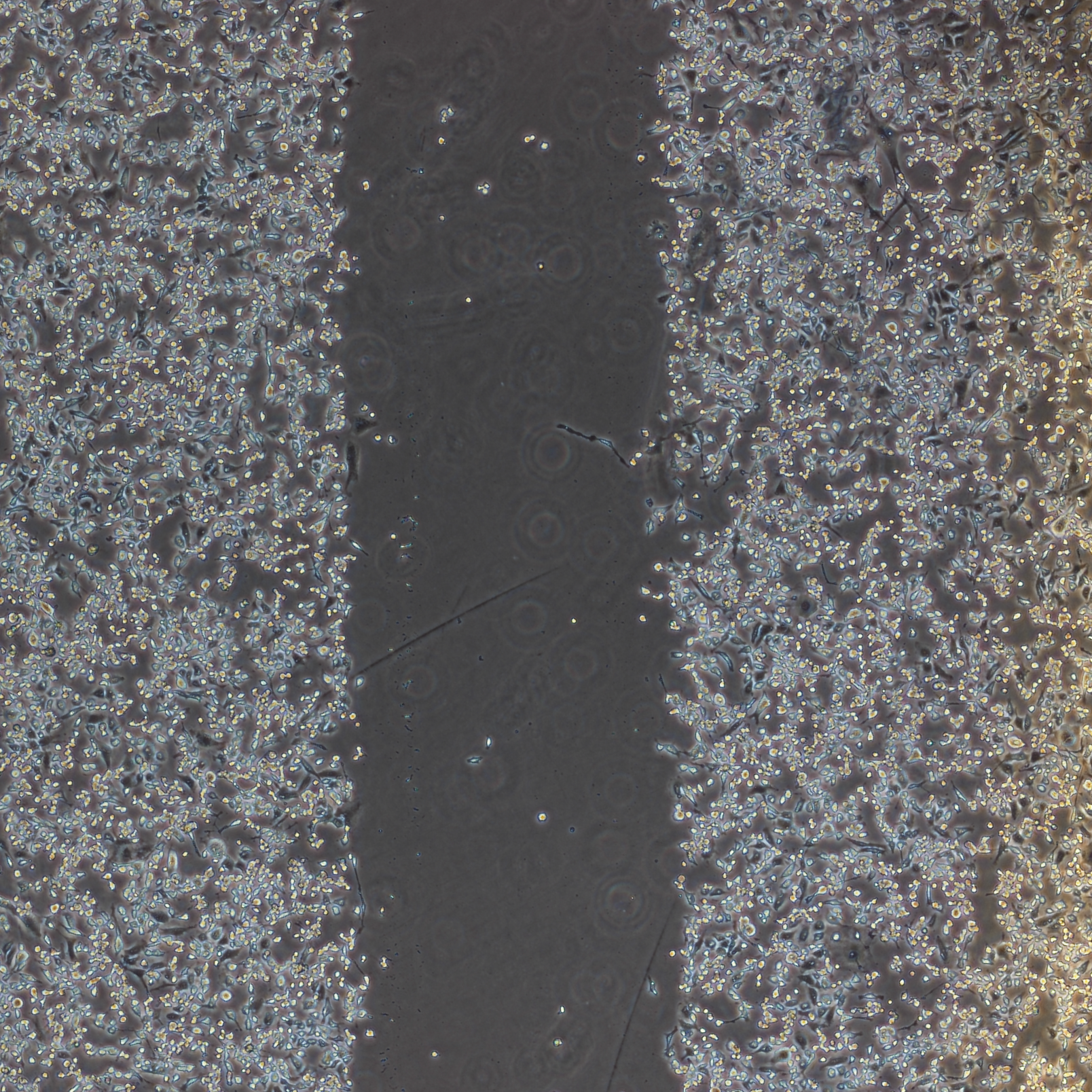


1975-MRPL13si-2-24


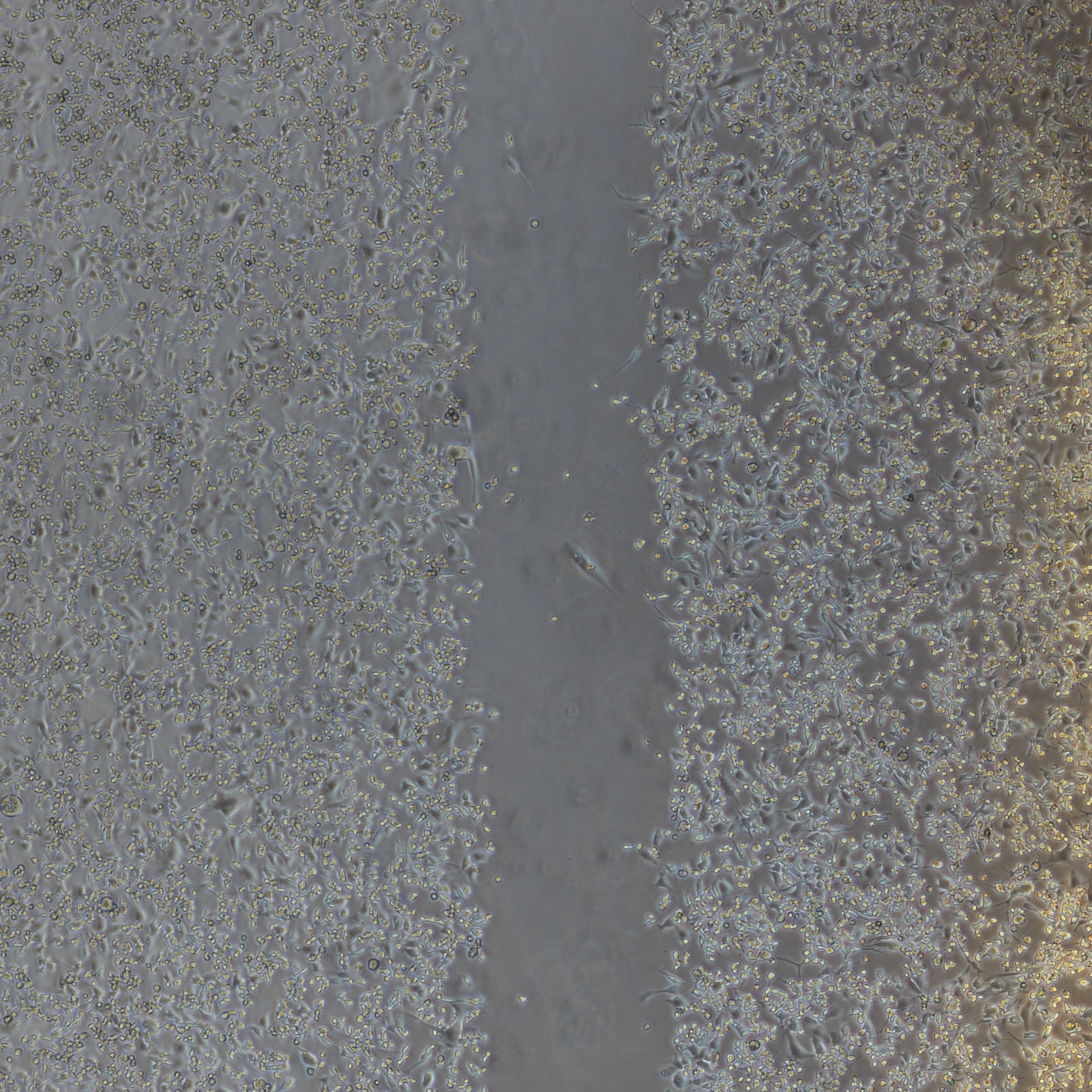


1975-MRPL13si-2-48

| group | time | frequency | IAMGE J AREA | Moving proportion |
| --- | --- | --- | --- | --- |
| A-549-MRPL13si-control | 0 | 1 | 1000229 | % |
|  | 24 | 1 | 580080 | 42.01 |
|  | 48 | 1 | 238798 | 76.13 |
|  |  |  |  |  |
| A-549-MRPL13si-1 | 0 | 1 | 864702 | % |
|  | 24 | 1 | 782932 | 9.46 |
|  | 48 | 1 | 633522 | 26.74 |
|  |  |  |  |  |
| A-549-MRPL13si-2 | 0 | 1 | 1062955 | % |
|  | 24 | 1 | 690450 | 35.04 |
|  | 48 | 1 | 538769 | 49.31 |
|  |  |  |  |  |
| 1975-MRPL13si-control | 0 | 1 | 933298 | % |
|  | 24 | 1 | 497786 | 46.66 |
|  | 48 | 1 | 226292 | 75.75 |
|  |  |  |  |  |
| 1975-MRPL13si-1 | 0 | 1 | 789243 | % |
|  | 24 | 1 | 448767 | 43.14 |
|  | 48 | 1 | 306163 | 61.21 |
|  |  |  |  |  |
| 1975-MRPL13si-2 | 0 | 1 | 897053 | % |
|  | 24 | 1 | 761776 | 15.08 |
|  | 48 | 1 | 442770 | 50.64 |
|  |  |  |  |  |
| A-549-MRPL13si-control | 0 | 2 | 1023236 | % |
|  | 24 | 2 | 562984 | 44.98 |
|  | 48 | 2 | 274022 | 73.22 |
|  |  |  |  |  |
| A-549-MRPL13si-1 | 0 | 2 | 855000 | % |
|  | 24 | 2 | 778135 | 8.99 |
|  | 48 | 2 | 607477 | 28.95 |
|  |  |  |  |  |
| A-549-MRPL13si-2 | 0 | 2 | 1006593 | % |
|  | 24 | 2 | 617544 | 38.65 |
|  | 48 | 2 | 540439 | 46.31 |
|  |  |  |  |  |
| 1975-MRPL13si-control | 0 | 2 | 900632 | % |
|  | 24 | 2 | 471480 | 47.65 |
|  | 48 | 2 | 183278 | 79.65 |
|  |  |  |  |  |
| 1975-MRPL13si-1 | 0 | 2 | 865321 | % |
|  | 24 | 2 | 496607 | 42.61 |
|  | 48 | 2 | 337215 | 61.03 |
|  |  |  |  |  |
| 1975-MRPL13si-2 | 0 | 2 | 1011233 | % |
|  | 24 | 2 | 842761 | 16.66 |
|  | 48 | 2 | 519167 | 48.66 |
|  |  |  |  |  |
| A-549-MRPL13si-control | 0 | 3 | 980065 | % |
|  | 24 | 3 | 525706 | 46.36 |
|  | 48 | 3 | 199443 | 79.65 |
|  |  |  |  |  |
| A-549-MRPL13si-1 | 0 | 3 | 900165 | % |
|  | 24 | 3 | 786294 | 12.65 |
|  | 48 | 3 | 657210 | 26.99 |
|  |  |  |  |  |
| A-549-MRPL13si-2 | 0 | 3 | 998654 | % |
|  | 24 | 3 | 632547 | 36.66 |
|  | 48 | 3 | 496131 | 50.32 |
|  |  |  |  |  |
| 1975-MRPL13si-control | 0 | 3 | 966541 | % |
|  | 24 | 3 | 534593 | 44.69 |
|  | 48 | 3 | 204133 | 78.88 |
|  |  |  |  |  |
| 1975-MRPL13si-1 | 0 | 3 | 1009632 | % |
|  | 24 | 3 | 589019 | 41.66 |
|  | 48 | 3 | 405064 | 59.88 |
|  |  |  |  |  |
| 1975-MRPL13si-2 | 0 | 3 | 988653 | % |
|  | 24 | 3 | 784595 | 20.64 |
|  | 48 | 3 | 497391 | 49.69 |
